# Cholinesterase Inhibitory and Anti-Inflammatory Activity of the Naphtho- and Thienobenzo-Triazole Photoproducts: Experimental and Computational Study

**DOI:** 10.3390/ijms241914676

**Published:** 2023-09-28

**Authors:** Milena Mlakić, Ivan Faraho, Ilijana Odak, Borislav Kovačević, Anamarija Raspudić, Ivana Šagud, Martina Bosnar, Irena Škorić, Danijela Barić

**Affiliations:** 1 Department of Organic Chemistry, Faculty of Chemical Engineering and Technology, University of Zagreb, Marulićev trg 19, HR-10000 Zagreb, Croatia; mdragojev@fkit.unizg.hr (M.M.); iskoric@fkit.unizg.hr (I.Š.); 2 Pharmacology in vitro, Selvita Ltd., Prilaz baruna Filipovića 29, HR-10000 Zagreb, Croatia; martina.bosnar@selvita.com; 3 Department of Chemistry, Faculty of Science and Education, University of Mostar, Matice Hrvatske bb, 88000 Mostar, Bosnia and Herzegovina; ilijana.odak@fpmoz.sum.ba (I.O.); anamarijaraspudic1@gmail.com (A.R.); 4 Group for Computational Life Sciences, Division of Physical Chemistry, Ruđer Bošković Institute, Bijenička Cesta 54, HR-10000 Zagreb, Croatia; borislav.kovacevic@irb.hr; 5 Croatian Agency for Medicinal Products and Medical Devices, Ksaverska Cesta 4, HR-10000 Zagreb, Croatia; ivana.sagud@halmed.hr

**Keywords:** anti-inflammatory activity, cholinesterase inhibition, molecular docking, molecular dynamics, naphtho/thienobenzo-triazoles, photochemical synthesis

## Abstract

New 1,2,3-triazolo(thieno)stilbenes were synthesized as mixtures of isomers and efficiently photochemically transformed to their corresponding substituted thienobenzo/naphtho-triazoles in high isolated yields. The resulting photoproducts were studied as acetyl- (AChE) and butyrylcholinesterase (BChE) inhibitors without or with interconnected inhibition potential of TNF-α cytokine production. The most promising anti-inflammatory activity was shown again by naphtho-triazoles, with a derivative featuring 4-pentenyl substituents exhibiting notable potential as a cholinesterase inhibitor. To identify interactions between ligands and the active site of cholinesterases, molecular docking was performed for the best potential inhibitors. Additionally, molecular dynamics simulations were employed to assess and validate the stability and flexibility of the protein–ligand complexes generated through docking.

## 1. Introduction

A small and simple triazole core is present in compounds that possess antimicrobial, antitumor, antitubercular, antidepressant, anti-inflammatory, and numerous other activities. Triazoles show a wide range of biological activities and are found in many powerful, biologically active compounds such as trazodone (antidepressant), hexaconazole (an antifungal drug), alprazolam (tranquilizer) and others [1]. So far, modifications of triazoles have proven to be very effective, and some of the newly prepared derivatives have been observed to have better activity and less toxicity. Drugs are also being developed for the treatment of neurodegenerative diseases such as Alzheimer’s disease (AD), with a characteristic 1,2,3-triazole core [2]. Triazolostilbenes have been synthesized in the context of nonlinear optical (NLO) materials [3,4]. In addition to the synthesis of new industrial materials, such as the triazole derivative of the natural pigment curcumin with significant biological and spectroscopic properties [5,6,7], functionalized *trans*-1,2,3-triazolostilbenes have been synthesized as suitable derivatives for further photochemical and photophysical research due to easily achievable change in their structures [8].

**Figure 1:**
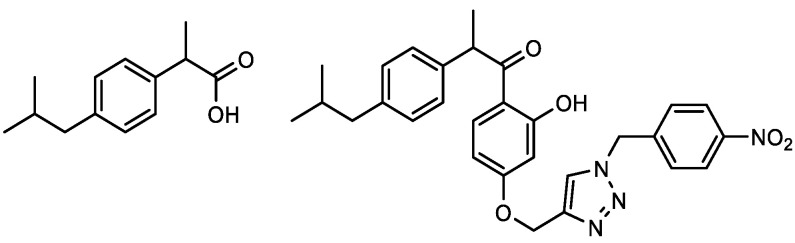
The structural formula of ibuprofen (**left**) and ibuprofen derivative (**right**) with 1,4-disubstituted 1,2,3-triazole nucleus.

**Figure 2:**
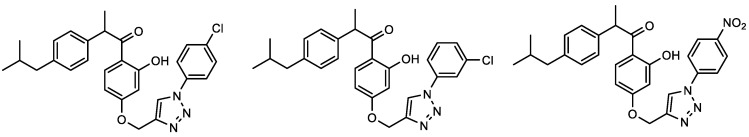
Ibuprofen derivatives with 1,4-disubstituted 1,2,3-triazole with significant anti-inflammatory activity.

Non-steroidal anti-inflammatory drugs have a wide therapeutic application in the treatment of various types of inflammatory conditions. The long-term use of these drugs has harmful effects on the gastrointestinal tract and subsequently leads to problems such as kidney damage, stomach ulcers, and hepatotoxicity [9]. This is exactly why there is an effort to synthesize new types of these compounds that should be highly effective and have improved safety so inflammations can be suppressed with as few unwanted effects as possible. Numerous studies related to such compounds have been conducted. Considering the biological and medical importance of ibuprofen (Figure 1) and 1,2,3-triazole, new chemical compounds were investigated based on these compounds’ previously known biological significance.

Ibuprofen-based compounds containing 1,4-disubstituted 1,2,3-triazole were synthesized and examined for in vivo anti-inflammatory activity, and one of the compounds (Figure 1) showed a strong effect as the reference anti-inflammatory drug ibuprofen at the same concentration (10 mg/kg body weight). The compounds in Figure 2 also showed significant anti-inflammatory activity. Their bactericidal profiles were also examined for all mentioned triazole analogs (including the ibuprofen derivative from Figure 1). All compounds showed significant bactericidal activity against Gram-positive and Gram-negative strains of bacteria. It is an interesting fact that the presence of an electron-withdrawing group or atom (NO_2_ or Cl) in the *meta*- or *para*-position (C3 or C4) of the benzyl or phenyl ring on triazole leads to a significant increase in anti-inflammatory and bactericidal activity [10].

**Figure 3:**
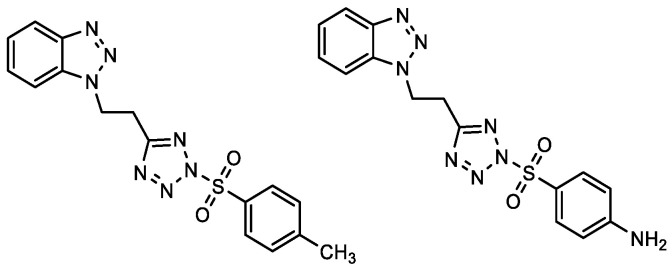
Structures of 1,2,3-triazole-tetrazole derivatives with confirmed anti-inflammatory activity.

Potential anti-inflammatory activity was also shown by compounds that contain both triazole and tetrazole rings in their structure (Figure 3). Their anti-inflammatory effect was evaluated by a test with carrageenan (a polysaccharide obtained from red seaweed), and they showed superior anti-inflammatory activity compared to other synthesized compounds [11,12].

On the other side, benzene-triazole derivatives (BTA) are active against a wide range of target molecules. Also, BTA is of biological and industrial importance [13,14]. Due to its pharmacological activities, 1*H*-benzo[*d*]-1,2,3-triazole is considered a favored structure. Used as a template for the design of new pharmacologically active compounds, BTA is undergoing rapid development in the synthesis of heterocycles. Research has shown that BTA has various pharmacological activities (anti-inflammatory, antimicrobial, antifungal, and anticarcinogenic). It is also known that molecules containing the benzotriazole core have CNS (Central Nervous System) activity [15].

The first confirmation of the strong connection between the cholinergic and immune systems appeared at the beginning of the last century by researching the effects of CNI-1493 on laboratory mouse models. It has been observed that the anti-inflammatory effects of the compound are indirect and occur due to the compound’s activity in the brain and not in the blood (immune cells) [16]. Further testing of the compound in animal models showed a dose-dependent reduction in the systemic production of tumor necrosis factor, TNF-α, after endotoxin (LPS) challenge [17]. It is known that the cholinergic anti-inflammatory system is a powerful instrument by which the body regulates the amount of inflammation. The key participant in this pathway is the neurotransmitter acetylcholine (ACh). Increased calcium levels in cells in that pathway lead to activation of the transcription factor NFαB and suppression of the immune response [18]. This mechanism can be interrupted by cholinesterase (ChE) enzymes that break down ACh, acetyl- (AChE), and butyrylcholinesterase (BChE) enzymes. Inhibitors of these enzymes could also have anti-inflammatory effects [19,20,21]. AChE and BChE also represent pharmacologically suitable targets in neurodegenerative disorders (Alzheimer’s disease, AD), given their physiological roles in the body. Treatment of neurodegenerative disorders currently includes common reversible cholinesterase enzyme inhibitors, such as galantamine, with proven efficacy in improving cognitive function [2,22,23].

**Figure 4:**
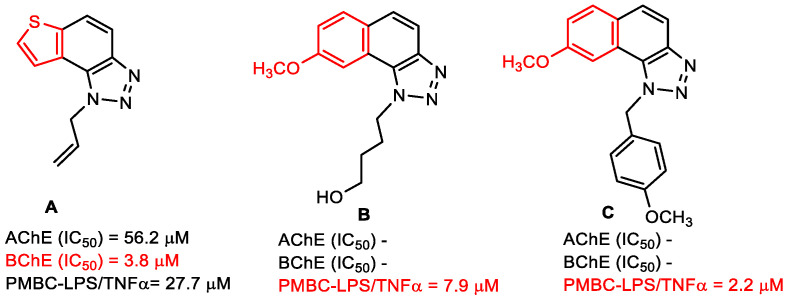
Thienobenzo/naphtho-triazole derivatives with improved cholinesterase inhibition and anti-inflammatory activity.

In our previous research, we have proven that some of the thienobenzo-triazoles obtained by photochemical cyclization have a dual activity. They showed very good cholinesterase enzyme inhibition interconnected with anti-inflammatory activity (Structure A, Figure 4) [24,25]. On the other hand, naphtho-triazoles (Structures B and C, Figure 4) showed a much more potent anti-inflammatory effect but not cholinesterase inhibition. This work evaluated a new series of naphtho/thienobenzo-triazoles to confirm previous experimental and computational data on biological activity. 

## 2. Results and Discussion

### 2.1. Synthesis of New Thienobenzo/Naphtho-Triazoles **1**–**13**

**Scheme 1:**
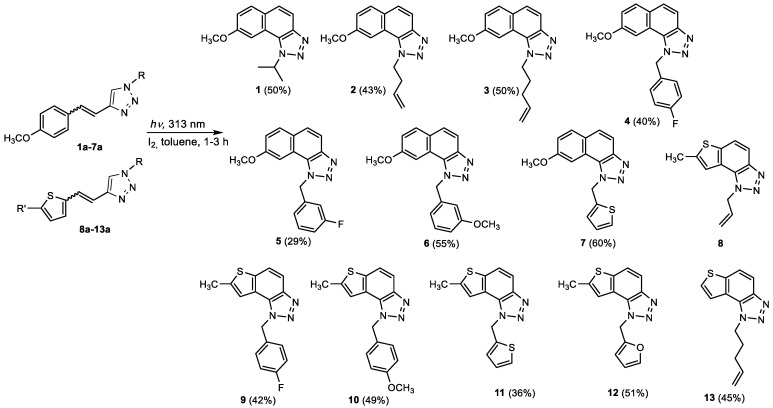
Synthetic pathway for the new thienobenzo/naphtho-triazoles **1**–**13**. Numbers given in parentheses represent isolated yields.

New triazolo-stilbenes **1a**–**7a** and triazolo-thienostilbenes **8a**–**13a** (Scheme 1) were prepared by the Wittig reaction from phosphonium bromide and the 1-substituted-1,2,3-triazole-4-carbaldehydes, according to the previously described procedure [26]. Wittig reaction provided the new triazolo-stilbenes **1a**–**7a** and triazolo-thienostilbenes **8a**–**13a** as mixtures of *cis*- and *trans*-isomers (isolated yields on the mixtures were 42–78%, Scheme 1) which were not separated in this research into pure geometrical isomers but immediately converted into photoproducts, naphtho-triazoles **1**–**7** and thienobenzo-triazoles **8**–**13** (Scheme 1) as new biological targets by an intramolecular electrocyclization reaction in high isolated yields (29–60%, Scheme 1). According to ^1^H NMR spectroscopy, the substituent on the triazole ring directed the ratio of geometric isomers in the Wittig reaction (see Materials and Methods). In the ^1^H NMR spectra of the mixture of geometrical isomers **1a**–**7a**, it can be seen the resolved patterns for ethylenic protons with the characteristic coupling constants, the signals for the protons on various substituents, and the singlets for the protons on the triazole rings.

In aerobic conditions, preparative irradiations of **1a**–**13a** at 313 nm in toluene solutions gave the naphthotriazoles **1**–**7** and thienobenzo-triazoles **8**–**13**; after that, they were successfully isolated and fully characterized by NMR spectroscopy and HRMS analyses (see Materials and Methods and Suppl. Material). The formation of the electrocyclization photoproducts **1**–**13** was generally accompanied by the formation of some high-molecular-weight products, which were not investigated. In their ^1^H NMR spectra, it can be seen the disappearance of the ethylenic protons and the singlets on the 1,2,3-triazole rings.

**Figure 5:**
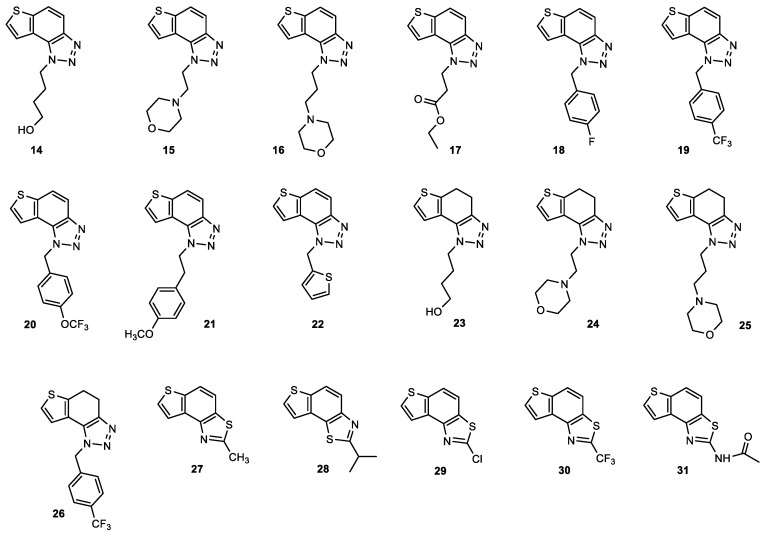
Tested thienobenzo-triazoles (**14**–**22**), their non-aromatic analogs (**23**–**26**), and thienobenzo-thiazoles (**27**–**31**) for cholinesterase inhibition [27] and anti-inflammatory activity.

As explained in previous research, the inhibitors of enzyme cholinesterases could also have anti-inflammatory effects [24]. For the stated reason, thienobenzo-triazoles **14**–**31** (Figure 5), which stood out as very good inhibitors of these enzymes, were also tested here for their possible interconnected inhibition potential of TNF-α cytokine production. Most of these molecules showed better inhibition of the enzyme butyrylcholinesterase (BChE) [27]. Also, the binding affinity of BChE for even seven new compounds was similar to that reported for common cholinesterase inhibitors.

### 2.2. Anti-Inflammatory Activity of 1,2,3-Triazole Derivatives 1–31

**Figure 6:**
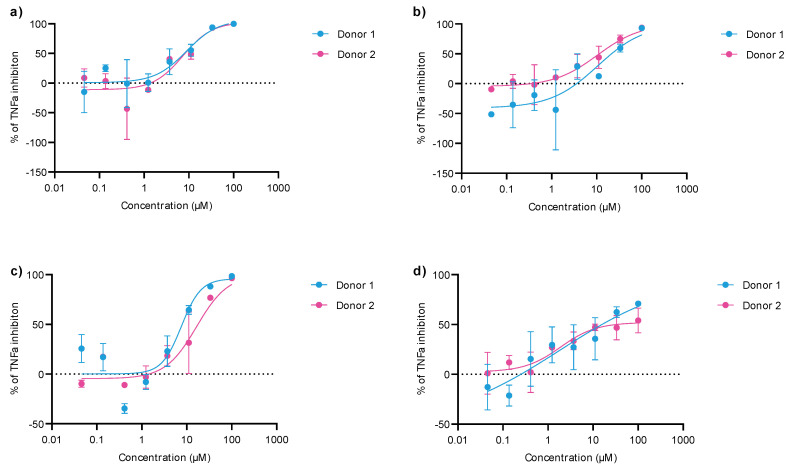
Naphthotriazoles **1** (**a**), **2** (**b**), **3** (**c**), and **6** (**d**) concentration-dependently inhibited TNF-α production in PBMCs stimulated with LPS, with average IC_50_ values in 2 donors 8.1 µM, 10.4 µM, 12.0 µM, and 1.9 µM, respectively.

The potential to act anti-inflammatory was evaluated for 1,2,3-triazole derivatives **1**–**31** in vitro in an assay where PBMCs from 2 healthy donors were stimulated with LPS to induce the immune response. As a measurement of response, the pro-inflammatory cytokine TNF-α was determined. The highest potency in TNF-α production inhibition was observed for naphtho-triazoles **1**–**4** and **6** (Figure 6) and thienobenzo-triazoles **18**–**22** and **26**–**27** (Table 1). However, cell viability was determined following incubation with compounds by measuring ATP levels inside the cells to ensure that the lower TNF-α production did not result from cytotoxicity. While the naphthotriazoles did not affect cell viability, for the most potent thienobenzo-triazoles **19**, **20,** and **26**, cell viability reduction was observed at the highest tested concentration.

### 2.3. Cholinesterase Inhibition of 1,2,3-Triazole Derivatives **1**–**13**

Since previously studied thienobenzo/naphtho-triazoles displayed activity toward cholinesterase inhibition, further functionalization and examination of these core units were imposed. The inhibition of AChE and BChE was assessed for seven naphtho-triazoles and six thienobenzo-triazole derivatives in a wide range of concentrations. If more than 50% inhibition is achieved, the IC_50_ value is determined (Table 2). Results were compared to a reference compound galantamine.

Naphtho-triazoles with methoxy substituent at aryl moiety and various substituents at triazole ring were studied previously [25] and showed no significant activity. Change in the type of substituent improved activity since almost all of the tested compounds achieved IC_50_ values. Derivatives **1**, **2**, **3,** and **7** showed activity with IC_50_ values in similar ranges of concentrations toward both enzymes. Among naphtho-triazoles, structure **3** with pentenyl substituent showed the most potent activity with IC_50_ values in a very good range (IC_50_ 51.3 µM for AChE and 53.5 µM for BChE), but still less than galantamine (IC_50_ 7.9 µM for BChE and 0.15 µM for AChE). Selective inhibition toward AChE was demonstrated by derivatives **5** and **6**, with *meta* substituents at phenyl moiety. The one with *m*-fluorophenyl substituent **5** achieved a very good IC_50_ value, while change of the fluorine atom to *para* position in derivative **4** diminished inhibitory activity. A similar structure to **1,** with a methyl group at aryl moiety, was studied earlier [24], and it can be concluded that the change to methoxy derivative improved activity toward AChE. At the same time, affinity toward BChE remained the same.

Previous results show that introducing a thiophene ring in the core of these structures improves their biological activity [25]. Numerous thienobenzo-triazoles with different substituents at the triazole ring were examined. They showed higher affinity toward BChE, where some of them, particularly derivative **A** (Figure 4), achieved better IC_50_ values than reference galantamine. Structure **13,** examined here, represents one in a series of such derivatives. The results are consistent with the previous ones, i.e., it showed selective inhibition for BChE with a very good IC_50_ value. The subsequent modification in the structure of the tested compounds refers to introducing a methyl substituent on the thiophene ring (compounds **8**–**12**). Derivatives with *p*-fluorophenyl substituent showed the best results among this group, while the same substituent had the opposite effect on naphtho–triazoles (derivative **4**). Generally, none of the compounds in this group displayed remarkable results since IC_50_ values, where achieved, are in the good to moderate range. A comparison of structure **8** with its analogs without methyl group (structure **A**) shows that introducing methyl at thiophene decreases the activity for BChE five times and completely reduces activity toward AChE. Reduction of activity toward BChE inhibition by introducing the methyl group was also observed for **9**, **10**, and **12** compared to their non-methylated analogs [27]. The presence of a furan ring at the triazole unit diminished enzyme activity, and 50% of inhibition was not achieved.

Among the tested compounds in this research, the two with pentenyl substituent, **3** and **13,** showed the best results, with one significant difference: naphtho derivative is active toward both enzymes while thienobenzo derivative is completely selective toward BChE. The introduction of methyl substituent at the thiophene ring is not a desirable structure modification since no improvement was observed for the inhibition of enzymes.

Concerning the evidence of a strong link between the cholinergic and immune systems [28], it is known that the cholinergic anti-inflammatory system, a part of the vagus nerve system, is a powerful tool by which the organism regulates the inflammation extent to ensure the elimination of the threat and prevent any potential tissue disruption or damage. The key player in this pathway is acetylcholine (ACh). As cholinesterases degrade ACh to acetic acid and choline, the inhibitors of these enzymes could also have anti-inflammatory effects.

As a result of experimental data in this research obtained by testing inhibition of cholinesterases and inhibition of TNF-α cytokine production, it can be concluded that naphtho-triazoles show better anti-inflammatory activity than thienobenzo-triazoles, especially compounds **1**, **2**, **3** and **6**. The best candidate showing the associated inhibition of cholinesterase and anti-inflammatory effect is naphtho-triazole **3**, for which the possibility of the described mechanism in which ACh is the link can be assumed.

### 2.4. Docking and Molecular Dynamics Study of 1,2,3-Triazole Derivatives 

**Figure 7:**
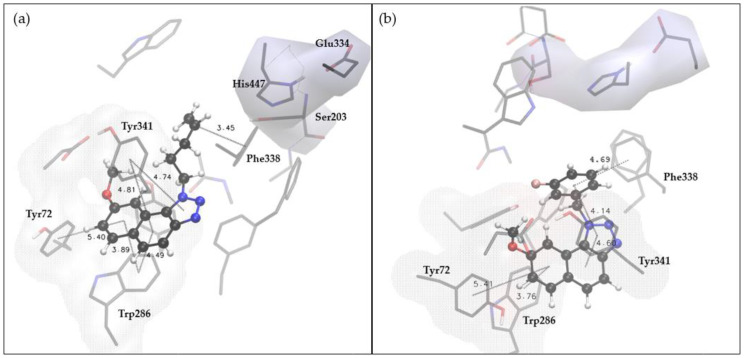
(**a**) Structure of the complex between compound **3** and the active site of AChE, obtained by molecular docking; (**b**) compound **5** docked into the active site of AChE. Distances are given in angstroms. Hydrogen atoms of the residues were omitted for clarity.

To elucidate the interactions underpinning the stability of complexes formed between the triazole derivatives and cholinesterases, we conducted molecular docking on the compounds that exhibited the most potent inhibitory activity in experimental assays. Notably, for AChE, the most promising outcomes were observed with compounds **3** and **5**. The structures of complexes obtained by docking **3** and **5** into the active site of AChE are shown in Figure 7a,b, respectively. 

These structural representations unveil the presence of non-covalent interactions between each ligand and residues within the active site, which are responsible for the complex’s stability. The placement of the most stable ligand pose into the active site of AChE for both molecules exhibits a striking similarity, which is to be expected given that their only structural divergence lies in the substituent at the triazole subunit. Both compounds **3** and **5** occupy the peripheral anionic site (PAS), thereby enabling the naphthalene subunit to engage in π-π stacking interactions with residues Trp286 and Tyr72, and the triazole ring to interact with Tyr341. The substituent at the triazole subunit in compound **3**, pentenyl, engages in a hydrophobic interaction with Phe338, while its counterpart in compound **5**, *m*-fluorobenzyl, interacts with the same residue through orthogonal π-π stacking. No hydrogen bonds are observed; this orientation places the triazole ring in a position where there are no residues that could act as hydrogen bond donors to nitrogen atoms of triazole. The lowest free energies of binding (Δ*G*_bind_) estimated by molecular docking of compounds **3** and **5** into AChE are −8.91 and −9.53 kcal mol^−1^, respectively (Table S1). Expectedly, these values are not as good as those estimated for the reference ligand galantamine, whose Δ*G*_bind_ of −10.11 kcal mol^−1^ was obtained using the same docking procedure.

**Figure 8:**
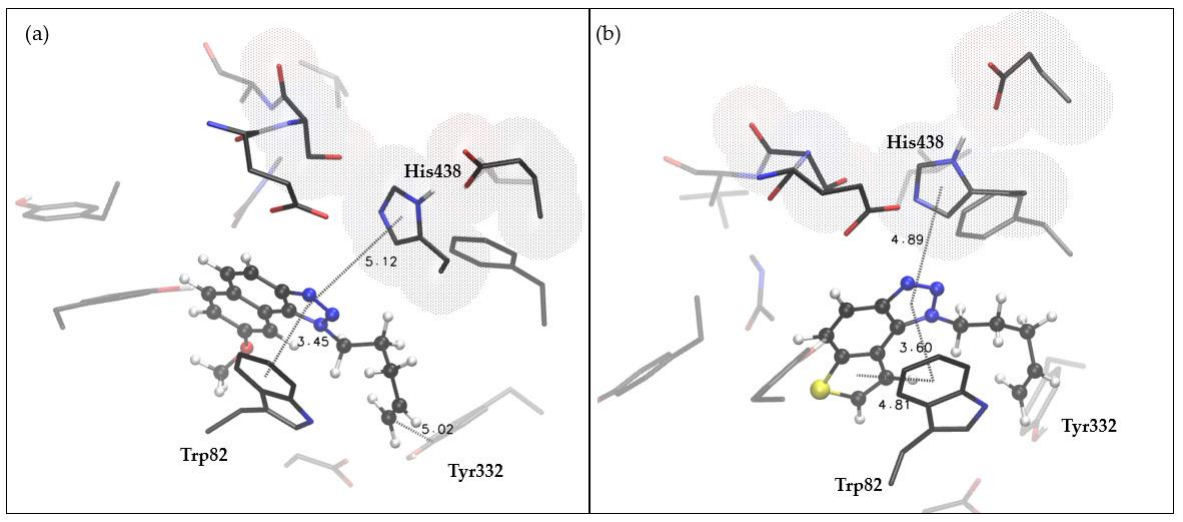
(**a**) Structure of the complex between compound **3** and the active site of BChE, obtained by molecular docking; (**b**) compound **13** docked into the active site of BChE. All distances in angstroms. Docked molecules are presented using a ball-and-stick model, with hydrogen atoms of residues omitted for clarity.

For BChE, the most potent inhibitory activity was observed with compounds **3** and **13**. The structures of the complexes formed by docking molecules **3** and **13** into the active site of BChE are depicted in Figure 8a,b. Despite the difference in the scaffold, where one of the phenyl rings in compound **3** is replaced by thiophene in compound **13**, both compounds exhibit similar placement within the active site.

This alignment enables π-π stacking interactions between the aromatic scaffold of both compounds and Trp82. Furthermore, on the opposing side of the ligand scaffold, π-π interaction occurs with His 438, residue belonging to the catalytic triad. In both of these ligands, the substituent at the triazole subunit is the same, 4-pentenyl, and its conformation allows for hydrophobic alkenyl-π interaction with Tyr332, a residue located within the PAS of BChE. Docking scores for these systems are presented in Table S2: the lowest free energies of binding estimated by molecular docking of compounds **3** and **13** into BChE are −7.56 and −6.81 kcal mol^−1^, respectively, while, for reference, the ligand galantamine Δ*G*_bind_ was estimated to be −7.49 kcal mol^−1^.

**Figure 9:**
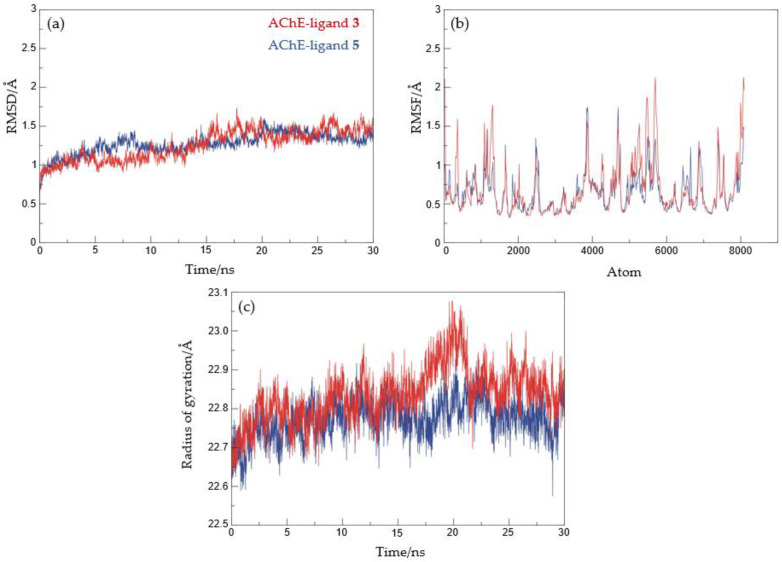
Molecular dynamics simulations of a protein–ligand complex of AChE with compounds **3** and **5**: (**a**) RMSD, (**b**) RMSF, and (**c**) radius of gyration plots.

Additionally, we conducted molecular dynamics (MD) simulations on studied systems to assess the stability of protein–ligand complexes suggested by molecular docking. For each investigated protein–ligand complex, the structure with the lowest estimated free energy of binding was chosen, and a 30 ns MD simulation was performed. Root-mean-square deviation values (RMSD) were computed for each system to gauge the structural alterations of the protein–ligand complex throughout the simulation time. RMSD analysis was conducted on all atoms within each protein–ligand complex, excluding hydrogen atoms. Additionally, RMS fluctuation values (RMSF) are calculated as average quadratic fluctuations of positions of α carbons over the trajectory. The RMSD quantifies how much a structure diverges from the initial one, and the RSMF values here show which parts of the protein backbone are the most mobile. Finally, the gyration radius (Rg) was computed to estimate the proteins’ compactness. Rg is calculated as a root-mean-square average of the distance of all atoms from the center of mass of the protein–ligand system. Systems where Rg does not increase significantly during the simulation can be described as compact. The results of MD simulations analysis for complexes of AChE with compounds **3** and **5**, respectively, are shown in Figure 9.

After an initial 5–10 ns period, both systems achieve convergence, with no significant fluctuations in subsequent RMSD values. Notably, the complex containing compound **3** (represented by the blue line in Figure 9a) exhibits slightly better performance. Across the entire blue trajectory, the average RMSD stands at 1.69 Å, with a maximum value of 2.01 Å, resulting in a 0.32 Å difference between the maximum and average RMSD values. An examination of the last 20 ns of this trajectory, where the system had converged, reveals an average RMSD of 1.34 Å and a maximum value of 1.58 Å, reducing the difference between them to 0.24 Å. For AChE with ligand **5** (illustrated by the red line in Figure 9a), the average RMSD value is 1.74 Å, accompanied by a maximum RMSD of 2.19 Å, resulting in a 0.45 Å difference. This difference decreases to 0.32 Å when the analysis is limited to the last 20 nanoseconds of the trajectory, where the average RMSD is 1.46 Å with a maximum value of 1.78 Å.

In both complexes, the RMSF values obtained during the entire simulation indicate that α-carbon positions in the protein backbone exhibit small fluctuations compared to their reference positions. Specifically, in the system containing ligand **3**, these fluctuations range from 0.34 to 1.75 Å, while in AChE complexed with ligand **5**, α-carbon positions vary between 0.32 and 2.13 Å. Similarly, the radius of gyration does not change significantly throughout the simulation; for a complex of AChE with **3**, this parameter varies within the range of 22.57 to 22.93 Å, while in the AChE with ligand **5**, the values span from 22.60 to 23.08 Å.

**Figure 10:**
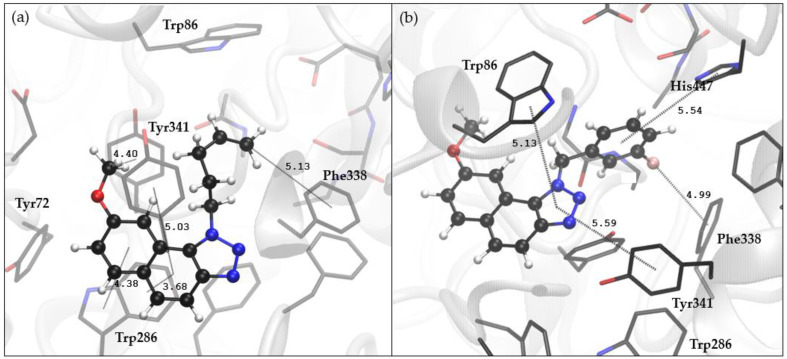
Protein–ligand complexes derived from MD simulation as structures with the least RMSD from the calculated average structures. (**a**) The active site of AChE with ligand **3**; (**b**) AChE active site with ligand **5**. Ligands are presented using a ball-and-stick model, with hydrogen atoms of residues omitted for clarity.

To gain a visual understanding of the complexes under study in molecular dynamics (MD), we analyzed the final 20 ns of each simulation to derive their average structure. Then, the structure with the least RMSD compared to the calculated average structure was selected for each of the two complexes. Figure 10 showcases detailed representations of the AChE active site with ligands **3** and **5** taken from these selected protein–ligand complex structures.

Similar to the complexes generated through docking, structures closest to the average ones obtained through MD resemble ligand placement within the peripheral anionic site of the AChE active site. The predominant stabilizing interaction observed is again π-π stacking. Ligand **3** maintains contact with Trp286 and Tyr341, whereas ligand **5** shifts away from Trp286 towards the His447 of the esteratic site and establishes contact with Trp86.

**Figure 11:**
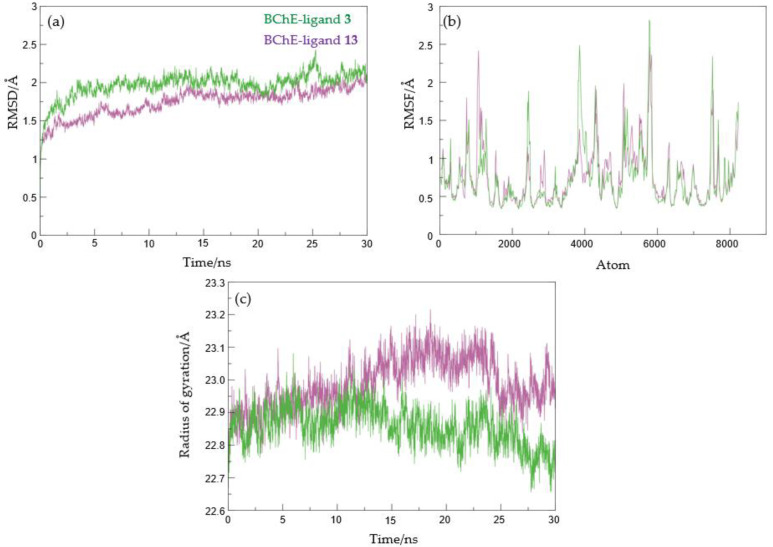
Molecular dynamics simulations of a protein–ligand complex of BChE with compounds **3** and **13**: (**a**) RMSD, (**b**) RMSF, and (**c**) radius of gyration plots.

The analysis of MD simulation results for BChE complexes with compounds **3** and **13** is shown in Figure 11. These complexes exhibit structural stability throughout the simulations, with average RMSD values of 1.97 Å and 1.74 Å, and maximum values of 2.43 Å and 2.17 Å, respectively. During the last 20 nanoseconds of the trajectories, the average RMSD decreases to 1.52 Å and 1.64 Å, with maxima at 1.90 Å and 2.16 Å. Ligand **3** induces more pronounced structural changes in the protein than molecule **13**, as evident from the RMSF values: RMS fluctuation reaches 2.82 Å in the complex with **3** and 2.47 Å for compound **13**. The compactness of the protein remains stable during the simulations, with gyration radius values similar to those observed in the complexes of AChE.

**Figure 12:**
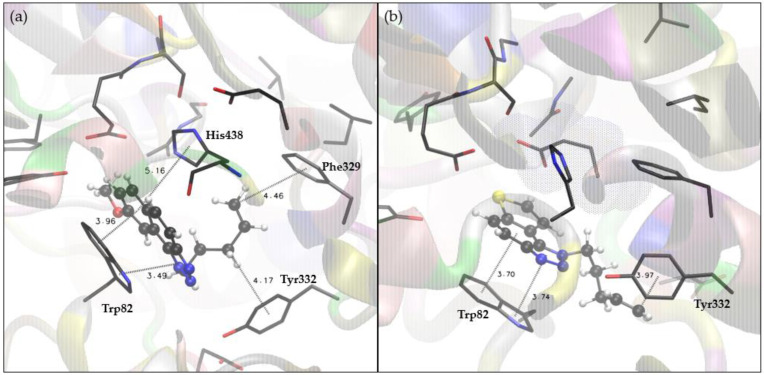
Protein–ligand complexes derived from MD simulation as structures with the least RMSD from the calculated average structures. (**a**) The active site of BChE with ligand **3**; (**b**) BChE active site with ligand **13**. Ligands are presented using a ball-and-stick model, with hydrogen atoms of residues omitted for clarity.

The active site of BChE with ligands **3** and **13** that are structurally closest to the calculated average structures obtained by MD is presented in Figure 12. 

Despite the displacements and structural fluctuations during the simulation, the dominant structure in both BChE–ligand complexes derived by the docking study remained similar to the initial one, thus preserving all relevant stabilizing interactions observed earlier: the π-π stacking between residue Trp82 and ligand, the distance from His438 in the esteratic subsite, and, finally, the contact between the 4-pentenyl, substituent attached to the triazole ring, andTyr332 of PAS.

### 2.5. Genotoxicity Testing on 1,2,3-Triazole Derivatives **1**–**31**

Pharmaceutical development of new drugs requires a thorough investigation into all impurities present in the active pharmaceutical compound (API) and the finished drug product. Impurities that are or can be theoretically present in the API, as well as in each of the intermediates in every step of the manufacturing process of the API, as well as the finished drug, have to be evaluated for their genotoxic potential. The primary evaluations are always done with the in silico models. So, within the scope of impurities of drug substances and drug products, this special subcategory of mutagenic/cancerogenic impurities is extremely important, and all possible risks must be evaluated. These compounds are more strictly regulated and controlled at much lower levels than other impurities. The regulation that is followed for them is the ICH M7 Guideline, and the levels that can be present in the drug substance or the drug product have to be calculated individually for each identified PMI (possible mutagenic compound) based on their determined acceptable daily intake (AI) and the maximum daily dose (MDD) of the final dosage form in question. Suppose toxicological studies on animals have not determined the AI. In that case, the most conservative approach must be taken with the most strict presumed AI described in the guideline itself.

When developing new API and finished drugs, it is expected that the impurities will also be new compounds and that no experimental data will be available for them in the genotoxicity databases. In these cases, the Q(SAR) approach is of vital importance. (Q)SAR models predict biological activity based on structural components [29]. This approach used to determine the mutagenic potential of impurities can also be used during the early stages of searching for potentially active drug substances. The elimination of all compounds that, even if they have biological activity, can also have mutagenic potential can be made fast and easy. The most commonly used tool is the *Lhasa Nexus v.2.5.2* software because it uses two complementary models, and their predictions are then reviewed one more time by an expert.

In the case of compounds **1**–**31** investigated in this paper, several compounds had the *Sarah Nexus v.3.2.1* software predictions positive (Table 3). Still, only one structure was found to have a very high risk of being genotoxic. Structures **5** and **6** had very low probability, according to Sarah’s prediction, and all of the others (**7**, **9**–**11**, **15**, **18**, **21**, **22**, **24**, **27**, **29**, and **30**) had the model rely on the data for the thiophene ring, with also a relatively low percentage of certainty.

As for structure **31**, here the probability is very high, and this structure also has a Derek (*Derek Nexus v.6.2.1 software*); positive prediction, with a much higher certainty. Here, the Q(SAR) found a very similar compound (condensed aromatic system with the amide functionality linked directly) with a positive AMES test for mutagenicity in vitro. This can be taken as a compound that can be eliminated from further research because the probability of its genotoxic potential is high.

## 3. Materials and Methods

### 3.1. General Remarks

Nuclear magnetic resonance (NMR) spectroscopic data for ^1^H and ^13^C nuclei were recorded at room temperature on Bruker Avance 300 and 600 MHz (Coventry, UK) spectrometers. Deuterated chloroform, CDCl_3_, with TMS as a standard, was used to record NMR spectra. All solvents used are commercially available and purified by distillation. Anhydrous MgSO_4_ was used to dry the organic layers after extraction. Column chromatography was performed on silica gel columns (60 Å, technical grade). Abbreviations used in this experimental procedure were NMR—nuclear magnetic resonance; EtOAc—ethyl acetate; PE—petroleum ether; E—diethyl ether; EtOH—ethanol; MeOH—methanol; and DCM—dichloromethane. Preparative photochemical reactions were carried out in a closed quartz cuvette in a Rayonet photochemical reactor equipped with 313 nm UV lamps. HRMS analyses were performed with a mass spectrometer (MALDI TOF/TOF analyzer) (Agilent Technologies, Santa Clara, CA, USA) equipped with a Nd:YAG laser operating at 355 nm with a firing rate of 200 Hz in a positive (H^+^) or negative (H^–^) ion reflector. All solvents were removed from the solutions using a rotary evaporator under reduced pressure.

### 3.2. Synthesis of Starting Thieno-Triazole Stilbenes **1a**–**13a**

Starting compounds **1a**–**13a** were obtained as mixtures of *cis*- and *trans*-isomers of heterostilbene synthesized by the Wittig reaction. The reaction apparatus was purged with nitrogen for 15 min before adding the reagents. Solutions of 2-thienyl-phosphonium salt (11 mmol) were dissolved in 50 mL of absolute EtOH (dried on a 3 Å sieve) in three-necked round-bottomed flasks (100 mL). Solutions of sodium ethoxide (11 mmol, 1.1 equiv Na dissolved in 10 mL absolute ethanol) were added dropwise under strictly anhydrous conditions under a nitrogen atmosphere. Various triazole aldehydes (11 mmol) were added directly to the mixed solutions. The reaction mixtures were allowed to stir for 24 h at room temperature under a nitrogen bubble. After removing the solvent with a rotary evaporator under reduced pressure, the solid reaction mixtures were extracted with toluene p.a. (3 × 25 mL). The organic layers were dried over anhydrous MgSO_4_. The final products, a mixture of *cis*- and *trans*-isomers 1a–13a, were isolated by silica gel column chromatography using PE/E as eluent and confirmed via ^1^H NMR and ^13^C NMR spectroscopy and HRMS analyses.

### 3.3. Synthesis of Cyclization Photoproducts **1**–**13**

Mixtures of previously synthesized compounds **1a**–**13a** were dissolved in toluene p.a. (~2.5 × 10^−3^ M) and transferred to a quartz cuvette (50 mL) with the addition of a catalytic amount of iodine and illuminated with 10 UV lamps at 313 nm in a Rayonet photochemical reactor (The Southern New England Ultraviolet Co., Branford, USA) for 1–3 h to achieve almost complete conversion. After removing the solvent with a rotary evaporator under reduced pressure, photoproducts **1**–**13** were purified by column chromatography using PE/E (60%) as eluent and obtained in the first fractions.


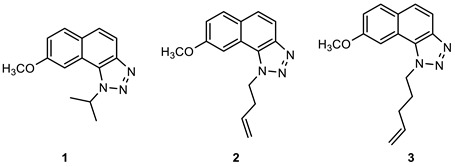


1-isopropyl-8-methoxy-1*H*-naphtho[1,2-*d*][1–3]triazole (1): 20 mg, 50% isolated yield; yellow oil; *R_f_* (PE/E (70%)) = 0.83; ^1^H NMR (CDCl_3_, 600 MHz) *δ*/ppm: 7.95 (d, *J* = 9.3 Hz, 1H), 7.86 (d, *J* = 8.8 Hz, 1H), 7.74 (d, *J* = 2.7 Hz, 1H), 7.64 (d, *J* = 9.0 Hz, 1H), 7.30 (dd, *J* = 8.8, 2.4 Hz, 1H), 5.50–5.44 (m, 1H), 4.00 (s, 3H), 1.91 (s, 3H), 1.90 (s, 3H); ^13^C NMR (CDCl_3_, 150 MHz) *δ*/ppm: 158.6, 144.9, 131.1, 128.5, 127.8, 125.9, 121.3, 116.0, 115.8, 104.0, 55.6, 53.4, and 22.7. MS (ESI) *m*/*z* (%, fragment): 242 (100); 200 (30); HRMS (*m*/*z*) for C_14_H_16_N_3_O: [M + H]+calcd = 241.1215, and [M + H] + measured = 241.1221.

1-(but-3-en-1-yl)-8-methoxy-1*H*-naphtho[1,2-*d*][1–3]triazole (2): 9 mg, 43% isolated yield; yellow oil; *R_f_* (PE/E (80%)) = 0.90; ^1^H NMR (CDCl_3_, 600 MHz) *δ*/ppm: 7.94 (d, *J* = 9.1 Hz, 1H), 7.85 (d, *J* = 8.4 Hz, 1H), 7.64 (d, *J* = 8.8 Hz, 2H), 7.30 (dd, *J* = 8.8, 2.9 Hz, 1H), 5.96–5.89 (m, 1H), 5.20–5.14 (m, 2H), 5.09 (t, *J* = 7.7 Hz, 2H), 3.98 (s, 3H), 2.89–2.85 (m, 2H); ^13^C NMR (CDCl_3_, 150 MHz) *δ*/ppm: 158.7, 145.0, 133.0, 131.1, 128.7, 127.8, 125.9, 121.0, 118.3, 116.8, 115.7, 103.3, 55.5, 49.9, and 34.2. MS (ESI) *m*/*z* (%, fragment): 254 (10); 167 (100); HRMS (*m*/*z*) for C_15_H_16_N_3_O: [M + H] + calcd = 253.1215, and [M + H]+measured = 253.1220.

8-methoxy-1-(pent-4-en-1-yl)-1*H*-naphtho[1,2-*d*][1–3]triazole (3): 9 mg, 50% isolated yield; yellow oil; R*_f_* (PE/E (80%)) = 0.71; ^1^H NMR (CDCl_3_, 600 MHz) *δ*/ppm: 7.94 (d, *J* = 9.3 Hz, 1H), 7.85 (d, *J* = 8.5 Hz, 1H), 7.64–7.62 (m, 2H), 7.29 (dd, *J* = 8.9, 2.5 Hz, 1H), 5.90–5.84 (m, 1H), 5.15–5.07 (m, 2H), 5.03 (t, *J* = 7.4 Hz, 2H), 3.99 (s, 3H), 2.28–2.19 (m, 4H); ^13^C NMR (CDCl_3_, 150 MHz) *δ*/ppm: 158.6, 145.0, 136.1, 131.1, 128.7, 127.8, 125.9, 121.1, 116.6, 116.5, 115.7, 103.5, 55.5, 50.1, 30.6, and 28.9. MS (ESI) *m*/*z* (%, fragment): 268 (100); 167 (15); HRMS (*m*/*z*) for C_16_H_18_N_3_O: [M + H]+calcd = 267.1371, and [M + H] + measured = 267.1376.


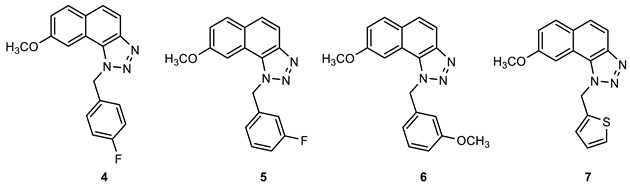


1-(4-fluorobenzyl)-8-methoxy-1*H*-naphtho[1,2-*d*][1–3]triazole (4): 12 mg, 40% isolated yield; yellow oil; *R_f_* (PE/E (70%)) = 0.85; ^1^H NMR (CDCl_3_, 600 MHz) *δ*/ppm: 7.90 (d, *J* = 5.3 Hz, 1H), 7.88 (d, *J* = 4.5 Hz, 1H), 7.66 (d, *J* = 8.8 Hz, 1H), 7.36 (d, *J* = 2.6 Hz, 1H), 7.21 (dd, *J* = 8.8, 2.3 Hz, 1H), 7.16–7.14 (m, 2H), 7.03 (t, *J* = 8.5 Hz, 2H), 6.24 (s, 2H), 3.75 (s, 3H); ^13^C NMR (CDCl_3_, 150 MHz) *δ*/ppm: 158.5, 145.3, 131.1, 130.8, 129.1, 127.9, 127.8, 126.3, 120.7, 117.6, 116.5, 116.2, 115.6, 103.4, 55.4, and 53.4. MS (ESI) *m*/*z* (%, fragment): 308 (100); HRMS (*m*/*z*) for C_18_H_15_N_3_OF: [M + H]+calcd = 307.1120, and [M + H] + measured = 307.1124.

1-(3-fluorobenzyl)-8-methoxy-1*H*-naphtho[1,2-*d*][1–3]triazole (5): 10 mg, 29% isolated yield; yellow oil; *R_f_* (PE/E (70%)) = 0.80; ^1^H NMR (CDCl_3_, 600 MHz) *δ*/ppm: 7.89 (d, *J* = 8.9 Hz, 1H), 7.88 (d, *J* = 9.1 Hz, 1H), 7.66 (d, *J* = 8.8 Hz, 1H), 7.33–7.29 (m, 2H), 7.20 (dd, *J* = 8.7, 2.3 Hz, 1H), 6.99 (ddt, *J* = 8.6, 7.3, 2.4 Hz, 1H), 6.96 (d, *J* = 7.9 Hz, 1H), 6.85 (d, *J* = 9.6 Hz, 1H), 6.26 (s, 2H), 3.74 (s, 3H); ^13^C NMR (CDCl_3_, 150 MHz) *δ*/ppm: 164.1, 162.5, 158.6, 145.3, 137.8, 131.9, 130.7, 129.1, 127.8, 126.3, 121.7, 120.6, 117.8, 115.3, 113.3, 103.2, 55.5, and 53.4. MS (ESI) *m*/*z* (%, fragment): 308 (100); HRMS (*m*/*z*) for C_18_H_15_N_3_OF: [M + H]+calcd = 307.1120, and [M + H] + measured = 307.1125.

8-methoxy-1-(3-methoxybenzyl)-1*H*-naphtho[1,2-*d*][1–3]triazole (6): 19 mg, 55% isolated yield; yellow oil; *R_f_* (PE/E (70%)) = 0.82; ^1^H NMR (CDCl_3_, 600 MHz) *δ*/ppm: 7.88 (d, *J* = 8.1 Hz, 1H), 7.85 (d, *J* = 9.7 Hz, 1H), 7.64 (d, *J* = 8.9 Hz, 1H), 7.38 (d, *J* = 2.5 Hz, 1H), 7.24 (d, *J* = 8.4 Hz, 1H), 7.18 (dd, *J* = 8.9, 2.5 Hz, 1H), 6.82 (dd, *J* = 8.1, 2.3 Hz, 1H), 6.78 (dd, *J* = 7.7, 1.1 Hz, 1H), 6.67 (s, 1H), 6.22 (s, 2H), 3.72 (s, 3H), 3.68 (s, 3H); ^13^C NMR (CDCl_3_, 150 MHz) *δ*/ppm: 160.4, 158.5, 145.3, 136.9, 130.6, 130.3, 129.2, 127.7, 126.2, 120.8, 118.4, 117.9, 115.5, 113.5, 112.2, 103.3, 55.5, 55.2, and 53.9. MS (ESI) *m*/*z* (%, fragment): 320 (100), 250 (45); HRMS (*m*/*z*) for C_19_H_17_N_3_O_2_: [M + H]+calcd = 319.1321, and [M + H] + measured = 319.1323.

8-methoxy-1-(thiophen-2-ylmethyl)-1*H*-naphtho[1,2-*d*][1–3]triazole (7): 20 mg, 60% isolated yield; yellow oil; *R_f_* (PE/E (70%)) = 0.91; ^1^H NMR (CDCl_3_, 600 MHz) *δ*/ppm: 7.88 (d, *J* = 7.7 Hz, 1H), 7.87 (d, *J* = 7.7 Hz, 1H), 7.65 (d, *J* = 8.9 Hz, 1H), 7.55 (d, *J* = 2.4 Hz, 1H), 7.26–7.25 (m, 1H), 7.23 (dd, *J* = 9.1, 2.4 Hz, 1H), 6.95–6.93 (m, 2H), 6.39 (s, 2H), 3.85 (s, 3H); ^13^C NMR (CDCl_3_, 150 MHz) *δ*/ppm: 158.6, 145.2, 137.5, 130.8, 128.9, 127.8, 127.4, 126.2, 125.9, 120.8, 117.7, 115.5, 103.4, 55.6, and 49.7. MS (ESI) *m*/*z* (%, fragment): 296 (100); 167 (20); HRMS (*m*/*z*) for C_16_H_14_N_3_OS: [M + H]+calcd = 295.0779, and [M + H] + measured = 295.0781.


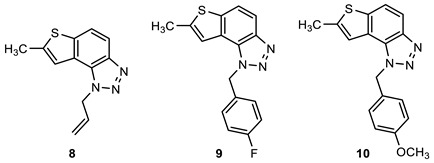


1-allyl-7-methyl-1*H*-thieno[3′,2′:3,4]benzo[1,2-*d*][1–3]triazole (8): 10 mg, 50% isolated yield; yellow oil; *R_f_* (PE/E (80%)) = 0.55; ^1^H NMR (CDCl_3_, 600 MHz) *δ*/ppm: 7.89 (d, *J* = 9.2 Hz, 1H), 7.89 (d, *J* = 8.5 Hz, 1H), 7.33 (s, 1H), 6.19–6.13 (m, 1H), 5.50–5.49 (m, 2H), 5.31 (d, *J* = 9.9 Hz, 1H), 5.09 (d, *J* = 16.7 Hz, 1H), 2.68 (s, 3H); ^13^C NMR (CDCl_3_, 150 MHz) *δ*/ppm: 144.5, 142.7, 139.2, 131.5, 128.2, 122.9, 118.8, 118.4, 118.2, 115.1, 51.5, and 16.3. MS (ESI) *m*/*z* (%, fragment): 232 (100); 230 (90); HRMS (*m*/*z*) for C_12_H_12_N_3_S: [M + H]+calcd = 229.0674, and [M + H] + measured = 229.0679.

1-(4-fluorobenzyl)-7-methyl-1*H*-thieno[3′,2′:3,4]benzo[1,2-*d*][1–3]triazole (9): 13 mg, 42% isolated yield; yellow oil; *R_f_* (PE/E (70%)) = 0.65; ^1^H NMR (CDCl_3_, 600 MHz) *δ*/ppm: 7.91 (d, *J* = 8.1 Hz, 1H), 7.69 (d, *J* = 8.8 Hz, 1H), 7.17 (dd, *J* = 8.7, 5.0 Hz, 2H), 7.14 (s, 1H), 7.03 (t, *J* = 9.1 Hz, 2H), 6.05 (s, 2H), 2.61 (s, 3H); ^13^C NMR (CDCl_3_, 150 MHz) *δ*/ppm: 163.4 (d, *J_C-F_* = 244.1 Hz), 161.7, 144.7, 142.9, 139.4, 131.1, 128.4, 128.1, 122.8, 119.1, 117.8, 116.1, 115.1, 52.3, and 16.3. MS (ESI) *m*/*z* (%, fragment): 298 (100); 227 (10); HRMS (*m*/*z*) for C_16_H_13_N_3_FS: [M + H]+calcd = 297.0735, and [M + H] + measured = 297.0736.

1-(4-methoxybenzyl)-7-methyl-1*H*-thieno[3′,2′:3,4]benzo[1,2-*d*][1–3]triazole (10): 14 mg, 49% isolated yield; yellow oil; *R_f_* (PE/E (80%)) = 0.62; ^1^H NMR (CDCl_3_, 600 MHz) *δ*/ppm: 7.69 (d, *J* = 8.7 Hz, 1H), 7.68 (d, *J* = 8.9 Hz, 1H), 7.20 (s, 1H), 7.14 (d, *J* = 8.6 Hz, 2H), 6.84 (d, *J* = 8.8 Hz, 2H), 7.02 (s, 2H), 3.76 (s, 3H), 2.62 (s, 3H); ^13^C NMR (CDCl_3_, 150 MHz) *δ*/ppm: 159.5, 144.7, 142.6, 139.3, 128.0, 127.4, 112.9, 118.9, 118.1, 115.0, 114.4, 55.1, 52.6, and 16.4. MS (ESI) *m*/*z* (%, fragment): 310 (80); 121 (100); HRMS (*m*/*z*) for C_17_H_16_N_3_OS: [M + H]+calcd = 309.0935, and [M + H] + measured = 309.0940.


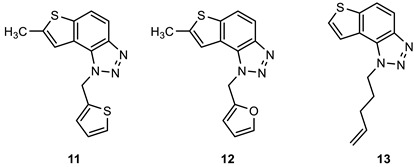


7-methyl-1-(thiophen-2-ylmethyl)-1*H*-thieno[3′,2′:3,4]benzo[1,2-*d*][1–3]triazole (11): 8 mg, 36% isolated yield; yellow oil; *R_f_* (PE/E (80%)) = 0.60; ^1^H NMR (CDCl_3_, 600 MHz) *δ*/ppm: 7.89 (d, *J* = 9.0 Hz, 1H), 7.68 (d, *J* = 8.7 Hz, 1H), 7.34 (s, 1H), 7.24 (d, *J* = 4.4 Hz, 1H), 7.00 (d, *J* = 3.7 Hz, 1H), 6.93 (d, *J* = 4.8, 3.8 Hz, 1H), 6.22 (s, 2H), 2.66 (s, 3H); ^13^C NMR (CDCl_3_, 150 MHz) *δ*/ppm: 144.6, 142.8, 139.5, 137.4, 127.8, 127.3, 126.4, 126.1, 122.9, 119.0, 117.9, 115.1, 46.2, and 16.4. MS (ESI) *m*/*z* (%, fragment): 286 (100); 97 (30); HRMS (*m*/*z*) for C_14_H_12_N_3_S_2_: [M + H]+calcd = 285.0394, and [M + H] + measured = 285.0392.

1-(furan-2-ylmethyl)-7-methyl-1*H*-thieno[3′,2′:3,4]benzo[1,2-*d*][1–3]triazole (12): 15 mg, 51% isolated yield; yellow oil; *R_f_* (PE/E (80%)) = 0.58; ^1^H NMR (CDCl_3_, 600 MHz) *δ*/ppm: 7.88 (d, *J* = 8.7 Hz, 1H), 7.69 (d, *J* = 8.7 Hz, 1H), 7.43 (s, 1H), 7.37 (s, 1H), 6.32 (s, 2H), 6.04 (s, 2H), 2.69 (s, 3H); ^13^C NMR (CDCl_3_, 150 MHz) *δ*/ppm: 148.3, 144.5, 143.0, 142.6, 139.5, 128.1, 123.1, 118.9, 118.2, 115.0, 110.9, 109.1, 46.3, and 16.3. MS (ESI) *m*/*z* (%, fragment): 270 (100); HRMS (*m*/*z*) for C_14_H_12_N_3_OS: [M + H] + calcd = 269.0623, and [M + H] + measured = 269.0627.

1-(pent-4-en-1-yl)-1*H*-thieno[3′,2′:3,4]benzo[1,2-*d*][1–3]triazole (13): 8 mg, 45% isolated yield; yellow oil; *R_f_* (PE/E (60%)) = 0.78; ^1^H NMR (CDCl_3_, 600 MHz) *δ*/ppm: 7.97 (d, *J* = 9.1 Hz, 1H), 7.80 (d, *J* = 9.4 Hz, 1H), 7.73 (d, *J* = 5.4 Hz, 1H), 7.68 (d, *J* = 5.7 Hz, 1H), 5.88–5.81 (m, 1H), 5.10 (d, *J* = 16.7 Hz, 1H), 5.07 (d, *J* = 9.2 Hz, 1H, 4.91 (t, *J* = 7.9 Hz, 2H), 2.23–2.15 (m, 4H); ^13^C NMR (CDCl_3_, 150 MHz) *δ*/ppm: 144.4, 139.8, 136.5, 128.5, 127.9, 122.6, 119.7, 118.9, 116.4, 116.1, 48.9, 30.5, and 29.0. MS (ESI) *m*/*z* (%, fragment): 244 (100); HRMS (*m*/*z*) for C_13_H_14_N_3_S: [M + H]+calcd = 243.0830, and [M + H] + measured = 243.0835.

### 3.4. In Vitro Biological Activity

**PBMC isolation.** Human peripheral blood mononuclear cells (PBMCs) were isolated from buffy coats obtained from healthy adult volunteers. The buffy coats were diluted with sterile PBS and layered over Lymphoprep (Axis-Shield Diagnostics, Dundee, UK). Tubes were centrifuged for 35 min at 400× *g* (RT; acceleration and break turned off). After centrifugation, the mononuclear ring was collected, transferred into a new 50 mL tube, and washed thrice with sterile PBS. The remaining erythrocytes were lysed with an isotonic solution of ammonium chloride (150 mM; Kemika, Zagreb, Croatia). The cell pellet was resuspended in Roswell Park Memorial Institute (RPMI) 1640 medium (Lonza, Basel, Switzerland) supplemented with 10% FBS (Biowest, Nuaillé France).

**Treatment and stimulation of human PBMCs.** Immediately after isolation, 200,000 PBMCs were seeded per well of a 96-well plate. Compounds were dissolved in 100% dimethyl sulfoxide (DMSO, Sigma, Darmstadt, Germany), and serial dilutions in DMSO were prepared and added to cells, with starting concentration for test compounds 100 µM or 1 µM for reference dexamethasone. After pre-incubation for 1h with the compounds, lipopolysaccharide (LPS) from E. coli 0111:B4 (Sigma, Darmstadt, Germany) was added to cells at 1 ng/mL final concentration. Plates were incubated for 24 h at 37 °C, 5% CO_2_, followed by the collection of supernatants for measurement of TNF-α and cell viability assessment.

**Enzyme-linked immunosorbent assay (ELISA) and cytotoxicity analysis.** TNFα concentration in PBMC supernatants was measured using ELISA assay, using antibodies obtained from BD Pharmingen (San Diego, CA, USA) and recombinant human TNFα protein (standard) from R&D Systems (Minneapolis, MN, USA). Lumitrac 600 384-well plates (Greiner Bio-One, Kremsmünster, Austria) were coated overnight with 1 µg/mL of TNFα capture antibody diluted in phosphate-buffered saline (PBS, Thermo Fisher Scientific, Waltham, MA, USA), followed by a washing step and blocking with 5% sucrose in assay diluent (1% bovine serum albumin (BSA; Sigma, Darmstadt, Germany) in PBS) for 4 h at RT. After the blocking step, the plates were washed, and samples/standards were added to the plates. After overnight incubation at 4 °C, detection antibodies diluted in assay diluent to a final concentration of 250 ng/mL were added. Following incubation for 2 h at RT, plates were washed, and streptavidin-HRP (Thermo Fisher Scientific, Waltham, MA, USA) solution was added (final concentration 500 ng/mL, prepared in assay diluent). Plates were incubated for 20 min at RT in the dark and washed, followed by adding Luminol substrate (Sigma, Darmstadt, Germany). Luminescence was measured using EnVision 2104 Multilabel Plate Reader (PerkinElmer, MA, USA), with an exposition time of 0.1 s. To evaluate cell viability, Cell Titer Glo reagent was used (Promega, Madison, WI, USA) according to the manufacturer’s instructions.

**Data analysis.** For ELISA, the blank was subtracted from measured RLU values, and TNFα concentrations in samples were interpolated from the standard curve. QC parameters for each plate were calculated from raw data. Percentages of inhibition of each compound were calculated from obtained cytokine concentrations using the formula: PIN = 100 − (((compound − no trigger)/(trigger − no trigger)) ∗ 100). For viability assessment, the average luminescence value was calculated from all trigger vehicle samples, and the percentage of vehicle value was calculated for all samples. The compound effect was considered cytotoxic if reduction from a vehicle was ≥20%, and these concentrations were excluded from further analysis. IC_50_ values of tested compounds were determined in GraphPad Prism 9 software using nonlinear regression curve fit (four parameters, with variable slope).

### 3.5. In Vitro ChE Activity Assay

The inhibition of acetylcholinesterase (AChE) and butyrylcholinesterase (BChE) was assessed using a modified spectrophotometric method based on Ellman’s approach [30]. AChE (EC from electric eel) and BChE (EC from equine serum), along with Trizma base, acetylthiocholine iodide (ATChI), S-butyrylthiocholine iodide (BTChI), and Galantamine were procured from Sigma-Aldrich (Darmstadt, Germany). Additionally, 5,50-dithiobis-(2-nitrobenzoic acid) (DTNB), known as Ellman’s reagent, was obtained from Zwijndrecht (Antwerpen, Belgium). Galantamine served as a reference standard in the experiment. The assessment of AChE/BChE activity was performed using a 96-well microplate reader (IRE 96, SFRI Medical Diagnostics, Saint Jean d’Illac, France) at 405 nm. The measurement lasted for 6 min at room temperature after the initiation of the enzymatic reaction. In each well of the microplate, the following components were present: 180 µL of Tris-HCl buffer (50 mM, pH 8.0), 10 µL of the respective enzyme (final concentration 0.03 U/mL), 10 µL of the tested solution with varying concentrations (final concentrations ranging from 10 to 350 μM, depending on solubility), and 10 μL of DTNB (final concentration 0.3 mM). The enzymatic reaction was initiated by adding 10 μL of ATChI/BTChI (final concentration of 0.5 mM prepared in Tris buffer) to each well. For the control measurement, a buffer solution was used in place of the tested compound. Additionally, for non-enzymatic hydrolysis, each measurement included a blank with the enzyme substituted by an equivalent buffer amount. To ensure accuracy, the experiment was conducted in triplicate. The solvent used in the experiment was ethanol. The percentage of enzyme inhibition was calculated using the measured data based on the equation: inhibition (%) = [(Ac − AT)/Ac] × 100, where Ac represents the enzyme activity without the test sample, and AT represents the enzyme activity with the test sample, calculated as mean values ± standard deviation. The IC_50_ value was determined through a nonlinear fit of compound concentration values using the mean inhibition data.

### 3.6. Computational Details

The geometry optimizations of the chosen ligands were performed using the Gaussian16 program package [31] at the M06-2X/6-31G(d) level of theory and then served as inputs for subsequent molecular docking. The molecular docking studies were conducted employing the Autodock program package [32]. The crystal structure 4EY7.pdb [33] from the Protein Data Bank was utilized for AChE, while 1P0I.pdb [34] was employed for BChE. Docking simulations were executed utilizing the Lamarckian Genetic Algorithm, generating 25 genetic algorithm dockings with 25 binding poses for each ligand. The residues of the enzymes were kept rigid during the docking. The most stable complexes between protein and ligand obtained by docking were used as starting structures for molecular dynamics study. Protein–ligand complexes were solvated with a truncated octahedron of the OPC water box and neutralized with Na+ ions using the Amber16 suite of programs [35]. The ff14SB force field [36] was employed for the protein part of the enzyme, and the GAFF force field [37] was used for ligands. Partial charges for ligands were derived using the RESP procedure. Equilibrations of all four systems involved energy minimizations and short (20 ps) MD simulations with systematic decreases to zero of the harmonic restraints and relaxation of the volume and temperature with target values of the temperature and pressure set to 300 K and 1 atm, respectively. Production MD simulation with no constraints was performed in the duration of 30,000 ps, under NPT conditions (300 K and 1 atm).

## 4. Concluding Remarks

New naphtho- and thienobenzo-triazoles were synthesized through irradiation as photoproducts derived from triazolo-stilbenes and triazolo-thienostilbenes synthesized using the Wittig reaction. In vitro evaluation of their anti-inflammatory potential demonstrated promising outcomes, with five naphtho-triazoles and seven thienobenzo-triazoles displaying effective inhibition of the proinflammatory cytokine TNF-*α*. Among the compounds assessed for their inhibitory activity against acetylcholinesterase and butyrylcholinesterase, three emerged as particularly promising: naphtho-triazoles **3** and **5**, and thieno-triazole **13**. Interestingly, compounds **3** and **13** share the same 4-pentenyl substituent on the triazole ring, yet only compound **3** exhibited inhibitory action on both cholinesterases, while **13** displayed selectivity towards BChE. Molecular docking studies of non-covalent complexes formed between these ligands and cholinesterases unveiled that the primary stabilizing interaction involved π-π stacking between the aromatic rings of the ligands, accompanied by a hydrophobic alkenyl-π interaction when the pentenyl substituent was present. Molecular dynamics simulations further confirmed the stability of these complexes. 

## Figures and Tables

**Table 1:** *In vitro* activity of novel 1,2,3-triazoles on inhibition of TNF-α production in PBMC stimulated with LPS. The IC_50_ values shown are average values from testing in 2 independent experiments (donors).

Compound	IC_50_/μM (av.) PMBC-LPS/TNF-α	Compound	IC_50_/μM (av.) PMBC-LPS/TNF-α
**1**	8.1	**17**	>100
**2**	10.4	**18**	27.7
**3**	12.0	**19**	10.2 **
**4**	19.1	**20**	8.9 **
**5**	>100	**21**	17.0
**6**	1.9	**22**	16.1
**7**	>100	**23**	>100
**8**	>100	**24**	>100
**9**	>100	**25**	>100
**10**	19.2	**26**	9.4 **
**11**	38.7	**27**	12.2
**12**	40.6	**28**	>100
**13**	21.3	**29**	>100
**14**	38.8	**30**	>100
**15**	n/a *	**31**	>100
**16**	>100	**dexamethasone**	0.007

* could not be calculated due to high variations. ** cell viability reduction at 100 µM.

**Table 2:** Inhibition of AChE and BChE and calculated IC_50_ values by compounds **1**–**13**.

Compound	AChE		BChE	
	IC_50_/μM	% Inhibition *	IC_50_/μM	% Inhibition *
**1**	329.0	58.1 ± 1.6 (500)	261.1	65.3 ± 0.7 (500)
**2**	91.8	77.9 ± 3.6 (500)	109.6	80.2 ± 0.7 (500)
**3**	51.3	64.8 ± 5.3 (250)	53.5	76.6 ± 1.8 (250)
**4**	-	35.1 ± 2.0 (500)	-	25.0 ± 0.5 (500)
**5**	55.5	80.8 ± 1.8 (250)	-	43.8 ± 1.8 (250)
**6**	176.7	78.6 ± 3.8 (250)	-	47.8 ± 0.5 (250)
**7**	109.5	81.4 ± 0.8 (500)	294.2	60.9 ± 0.4 (500)
**8**	-	43.5 ± 2.1 (350)	206.5	62.6 ± 0.2 (350)
**9**	83.0	78.8 ± 1.9 (350)	69.8	76.1 ± 0.8 (350)
**10**	66.6	83.6 ± 3.7 (350)	99.7	65.2 ± 0.3 (350)
**11**	217.7	71.3 ± 0.3 (350)	122.7	72.3 ± 5.6 (350)
**12**	-	41.8 ± 1.0 (350)	-	46.1 ± 4.(350)
**13**	-	46.4 ± 2.5 (250)	40.7	86.5 ± 5.0 (250)
**Galantamine**	0.15	90.0 ± 1.5 (60)	7.9	90.1 ± 3.4 (4.5)

* Numbers given in parentheses represent maximal concentrations tested in μM.

**Table 3:** Compounds with a positive mutagenic potential by Lhasa M7 evaluation.

Structure	ICH M7 Class	Derek Prediction	Sarah Prediction	Experimental Data	Overall In Silico
**5**	Class 5	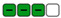	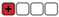	Carc: UnspecifiedAmes: Unspecified	Negative
**6**	Class 5	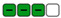	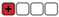	Carc: UnspecifiedAmes: Unspecified	Negative
**7**	Class 5	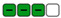	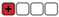	Carc: UnspecifiedAmes: Unspecified	Negative
**9**	Class 5	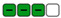	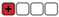	Carc: UnspecifiedAmes: Unspecified	Negative
**10**	Class 5	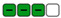	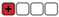	Carc: UnspecifiedAmes: Unspecified	Negative
**11**	Class 5	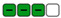	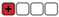	Carc: UnspecifiedAmes: Unspecified	Negative
**15**	Class 5	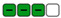	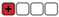	Carc: UnspecifiedAmes: Unspecified	Negative
**18**	Class 5	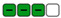	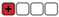	Carc: UnspecifiedAmes: Unspecified	Negative
**21**	Class 5	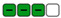	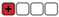	Carc: UnspecifiedAmes: Unspecified	Negative
**22**	Class 5	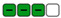	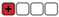	Carc: UnspecifiedAmes: Unspecified	Negative
**24**	Class 5	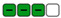	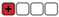	Carc: UnspecifiedAmes: Unspecified	Negative
**27**	Class 5	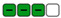	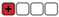	Carc: UnspecifiedAmes: Unspecified	Negative
**29**	Class 5	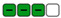	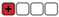	Carc: UnspecifiedAmes: Unspecified	Negative
**30**	Class 5	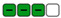	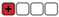	Carc: UnspecifiedAmes: Unspecified	Negative
**31**	Class 3	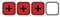	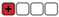	Carc: UnspecifiedAmes: Unspecified	Positive

## Data Availability

Additional data are available on request.

## Supplementary Materials

The following supporting information can be downloaded at: https://www.mdpi.com/article/10.3390/ijms241914676/s1, ^1^H and ^13^C NMR spectra of synthesized compounds **1**–**13** (Figures S1–S78); MS spectra and HRMS analyses of synthesized compounds **1**–**13** (Figures S79–S91); free energies of binding obtained by docking (Tables S1 and S2); RMSD, RMSF, and Rg for protein–ligand complexes derived by MD simulation (Tables S3 and S4).
