# Supplementary material for: Cholinesterase Inhibitory and Anti-Inflammatory Activity of the Naphtho- and Thienobenzo-Triazole Photoproducts: Experimental and Computational Study
Source: Int J Mol Sci. 2023 Sep 28;24(19):14676. doi: 10.3390/ijms241914676 (PMC10572743; doi:10.3390/ijms241914676)
Supplement: Supplementary file 1 [file ijms-24-14676-s001.zip › ijms-2622827-supplementary.pdf]

Supplementary material

Cholinesterase Inhibitory and Anti-Inflammatory Activity of the Naphtho- and Thienobenzo-Triazole Photoproducts: Experimental and Computational Study

Milena Mlakić [1], Ivan Faraho [2,*], Ilijana Odak [3], Borislav Kovačević [4], Anamarija Raspudić [3], Ivana Šagud [5], Martina Bosnar [2], Irena Škorić [1] and Danijela Barić [4,*]

[1] Department of Organic Chemistry, Faculty of Chemical Engineering and Technology, University of Zagreb, Marulićev trg 19, HR-10000 Zagreb, Croatia; mdragojev@fkit.unizg.hr (M.M.); iskoric@fkit.unizg.hr (I.Š.)
[2] Pharmacology in vitro, Selvita Ltd., Prilaz baruna Filipovića 29, HR-10000 Zagreb, Croatia; martina.bosnar@selvita.com
[3] Department of Chemistry, Faculty of Science and Education, University of Mostar, Matice Hrvatske bb, 88000 Mostar, Bosnia and Herzegovina; ilijana.odak@fpmoz.sum.ba (I.O.); anamarijaraspudic1@gmail.com (A.R.)
[4] Group for Computational Life Sciences, Division of Physical Chemistry, Ruđer Bošković Institute, Bijenička Cesta 54, HR-10000 Zagreb, Croatia; borislav.kovacevic@irb.hr
[5] Croatian Agency for Medicinal Products and Medical Devices, Ksaverska Cesta 4, HR-10000 Zagreb, Croatia; ivana.sagud@halmed.hr
* Correspondence: ivan.faraho@selvita.com (I.F.); dbaric@irb.hr (D.B.)

Contents

1. NMR spectra of naphtho- and thienobenzo-triazoles (S1-S78)........2

2. MS spectra and HRMS analyses of naphtho- and thienobenzo-triazoles (S79-S91)........41

3. Tables S1 and S2, free energies of binding obtained by docking........48

4. Tables S3 and S4, RMSD, RMSF, and Rg for protein-ligand complexes from MD simulations........49

1. NMR spectra of naphtho- and thienobenzo-triazoles

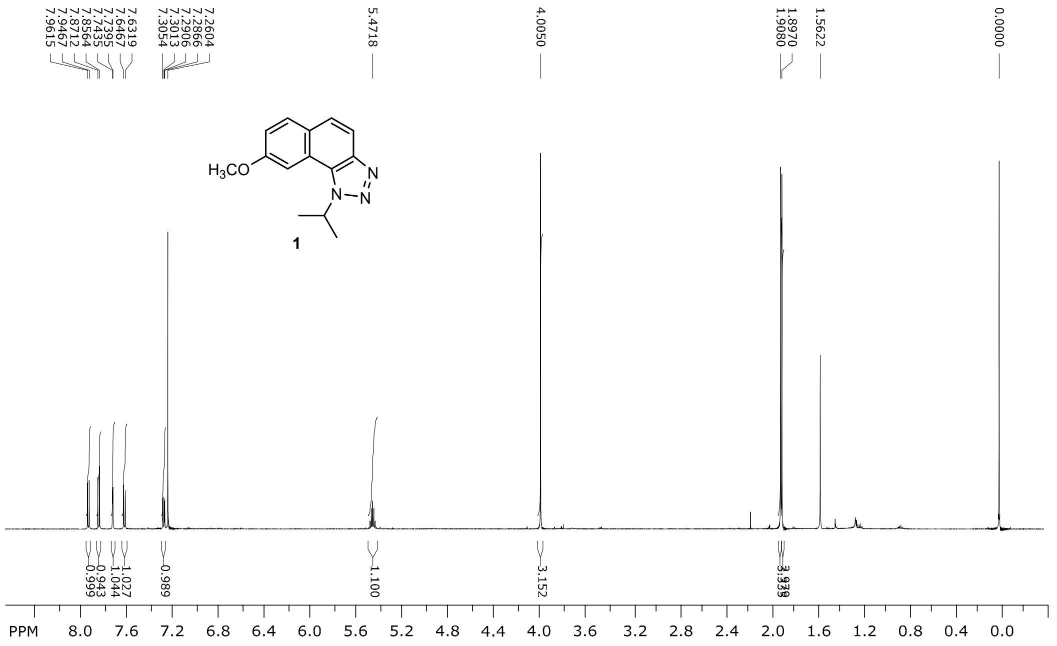

file: ...ža 2022-2023\NMR KK\KK-16(4-5)\fid expt: <zg30>
transmitter freq.: 600.135401 MHz
time domain size: 32768 points
width: 12019.23 Hz = 20.0275 ppm = 0.366798 Hz/pt
number of scans: 64

freq. of 0 ppm: 600.130011 MHz
processed size: 32768 complex points
LB: 0.000 GF: 0.0000
Hz/cm: 217.737 ppm/cm: 0.36281

Figure S1. ^{1}H NMR ($CDCl_3$) spectrum of **1**.

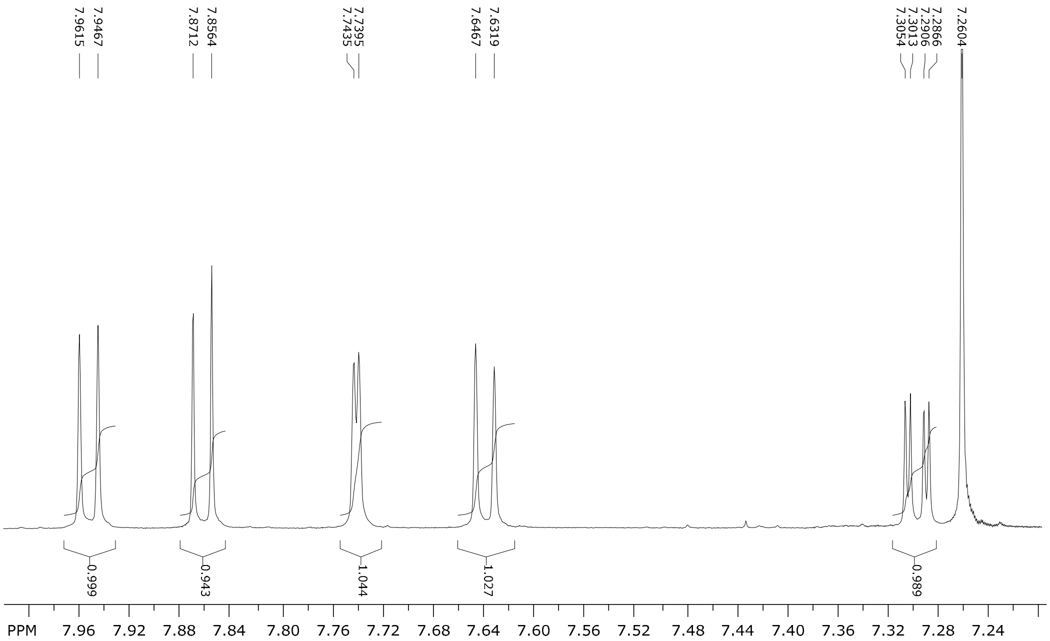

Figure S2. ^{1}H NMR ($CDCl_3$) spectrum of aromatic part of **1**.

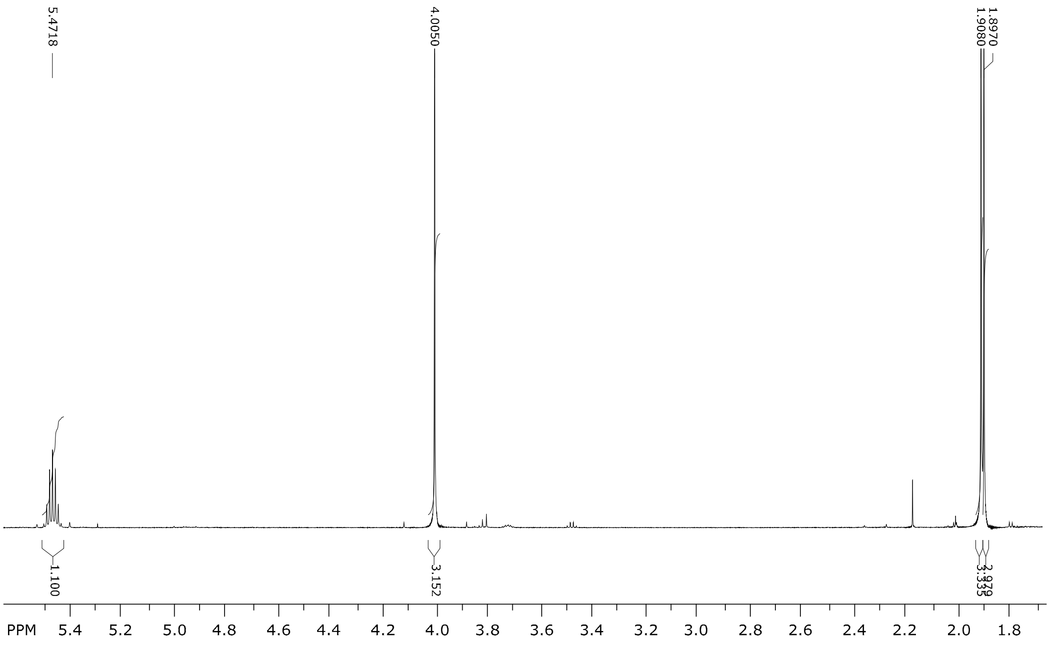

Figure S3. ^{1}H NMR ($CDCl_3$) spectrum of aliphatic part of **1**.

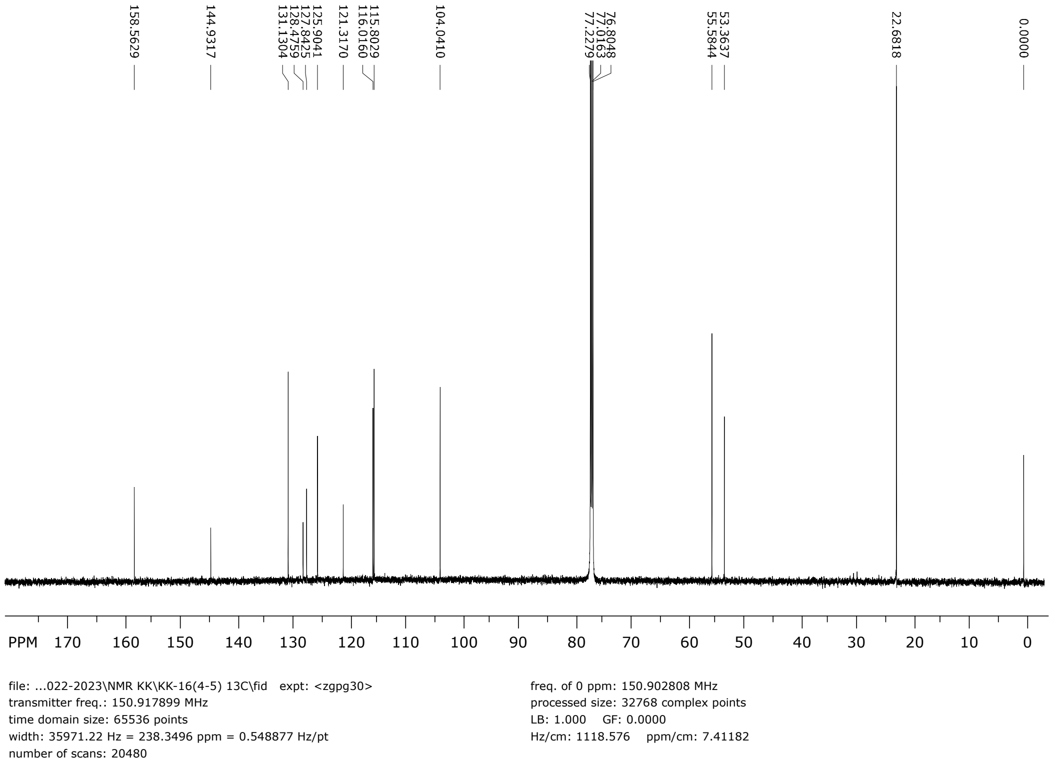

Figure S4. ^{13}C NMR (CDCl$_3$) spectrum of **1**.

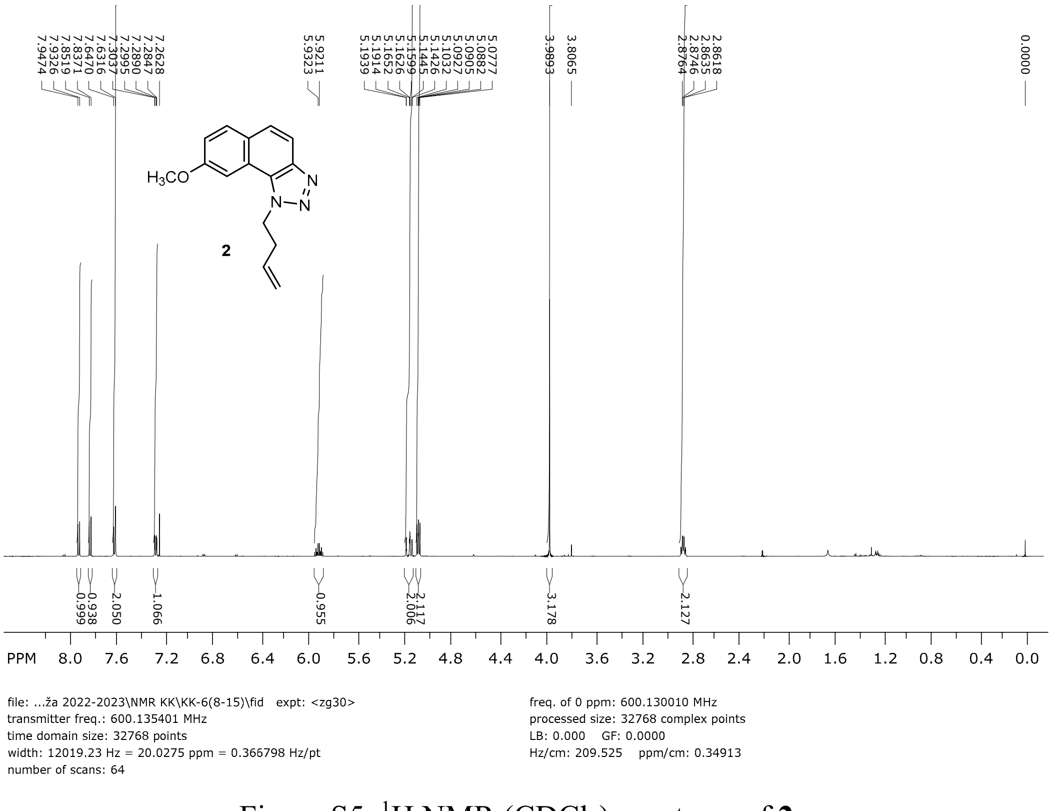

Figure S5. ^{1}H NMR (CDCl$_3$) spectrum of **2**.

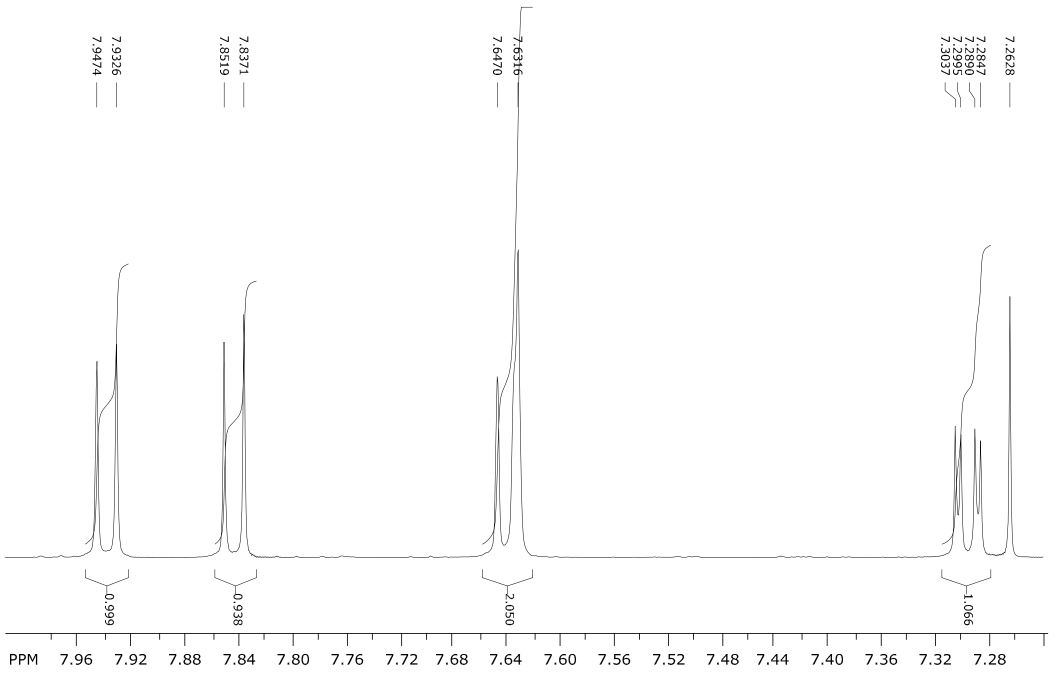

file: ...ža 2022-2023\NMR KK\KK-6(8-15)\fid expt: <zg30>
transmitter freq.: 600.135401 MHz
time domain size: 32768 points
width: 12019.23 Hz = 20.0275 ppm = 0.366798 Hz/pt
number of scans: 64

freq. of 0 ppm: 600.130010 MHz
processed size: 32768 complex points
LB: 0.000 GF: 0.0000
Hz/cm: 18.694 ppm/cm: 0.03115

Figure S6. ^{1}H NMR ($CDCl_3$) spectrum of aromatic part of **2**.

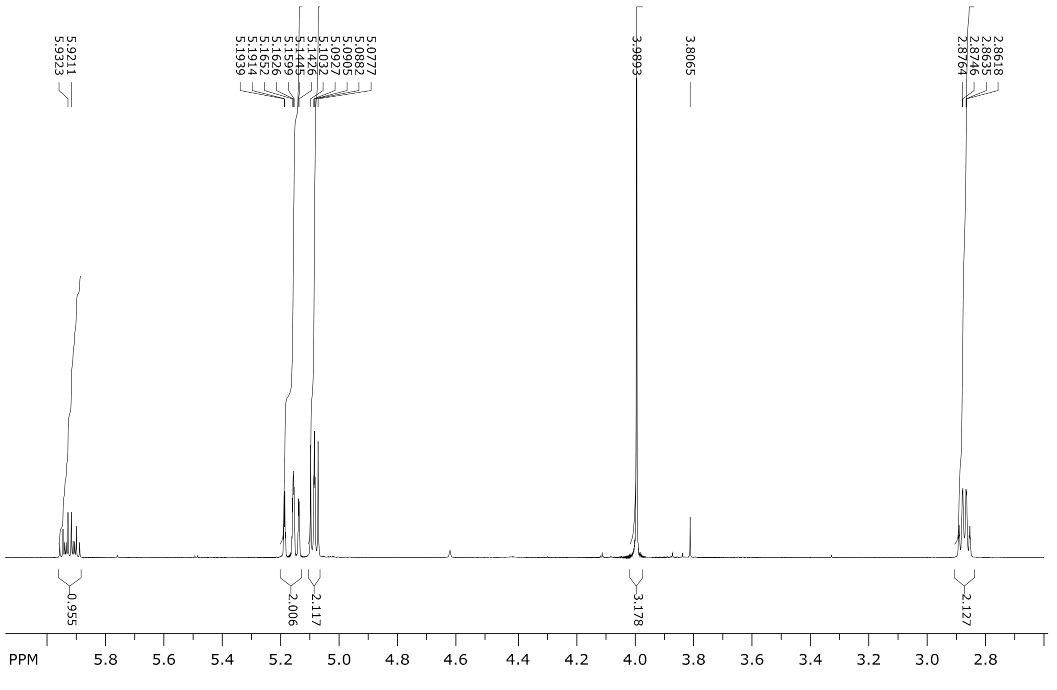

file: ...ža 2022-2023\NMR KK\KK-6(8-15)\fid expt: <zg30>
transmitter freq.: 600.135401 MHz
time domain size: 32768 points
width: 12019.23 Hz = 20.0275 ppm = 0.366798 Hz/pt
number of scans: 64

freq. of 0 ppm: 600.130010 MHz
processed size: 32768 complex points
LB: 0.000 GF: 0.0000
Hz/cm: 85.195 ppm/cm: 0.14196

Figure S7. ^{1}H NMR ($CDCl_3$) spectrum of aliphatic part of **2**.

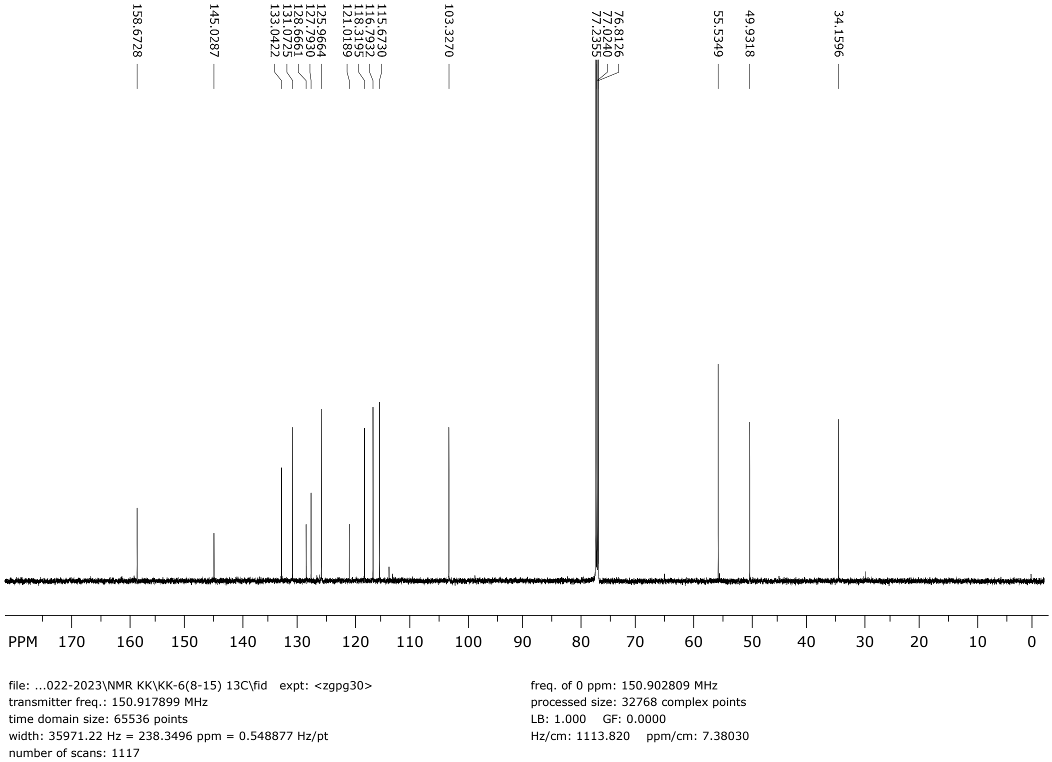

Figure S8. ^{13}C NMR (CDCl$_3$) spectrum of **2**.

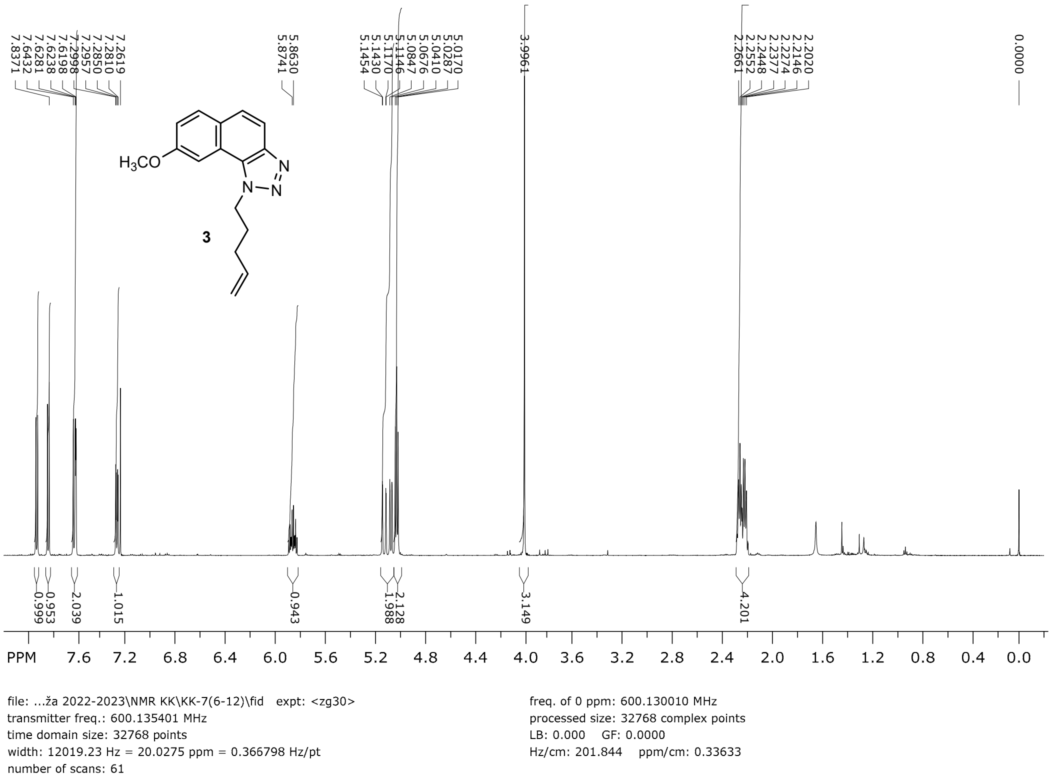

Figure S9. ^{1}H NMR (CDCl$_3$) spectrum of **3**.

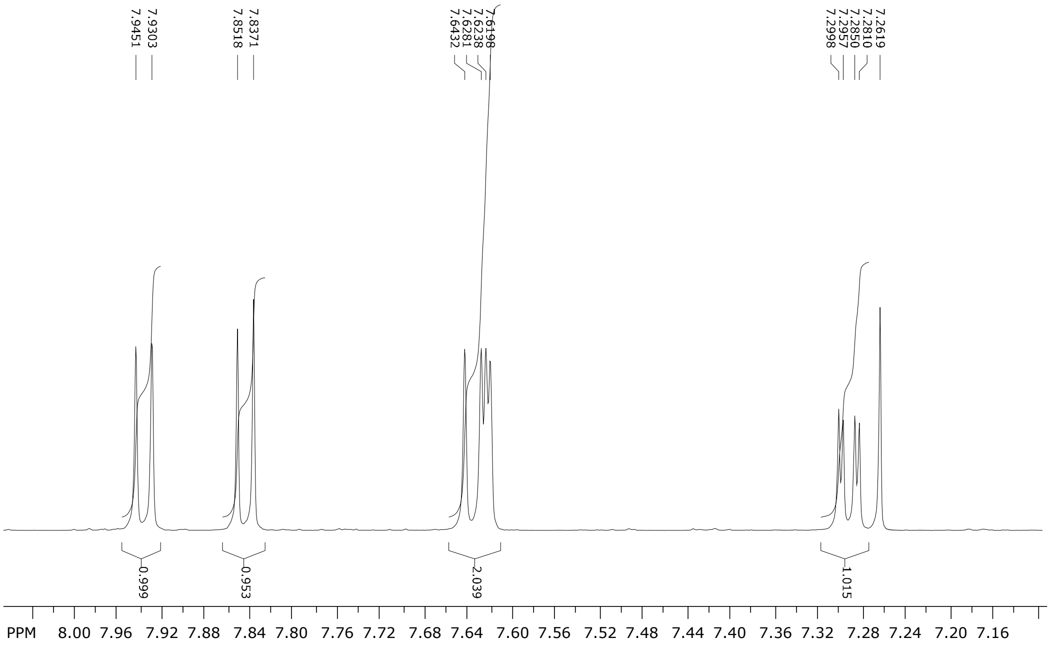

file: ...ža 2022-2023\NMR KK\KK-7(6-12)\fid expt: <zg30>
transmitter freq.: 600.135401 MHz
time domain size: 32768 points
width: 12019.23 Hz = 20.0275 ppm = 0.366798 Hz/pt
number of scans: 61

freq. of 0 ppm: 600.130010 MHz
processed size: 32768 complex points
LB: 0.000 GF: 0.0000
Hz/cm: 22.909 ppm/cm: 0.03817

Figure S10. ^{1}H NMR ($CDCl_3$) spectrum of aromatic part of **3**.

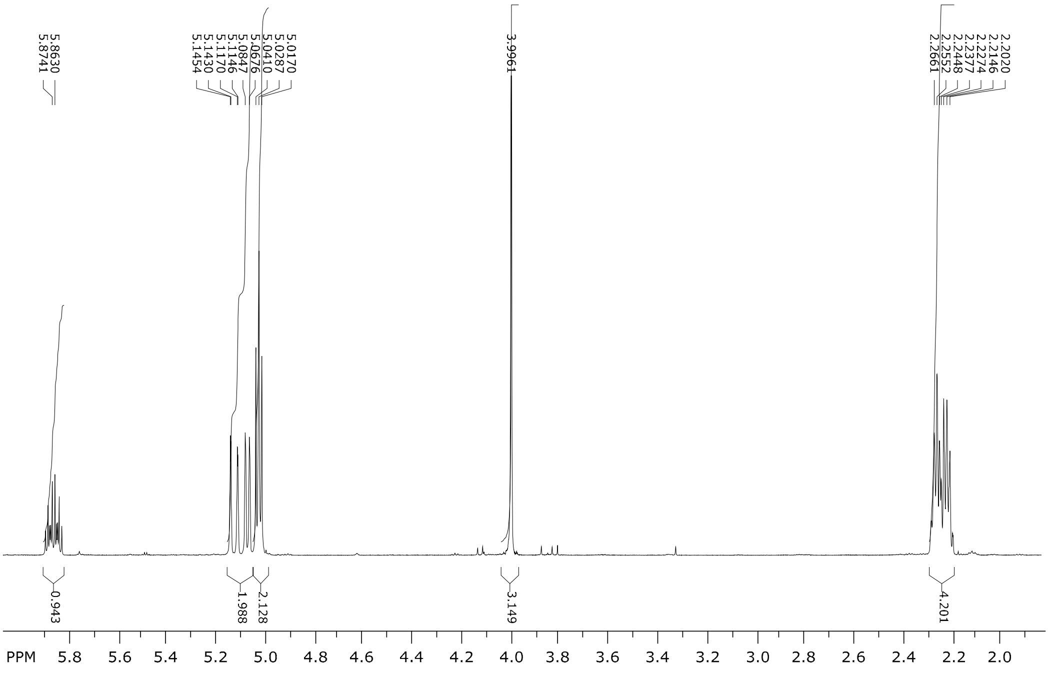

file: ...ža 2022-2023\NMR KK\KK-7(6-12)\fid expt: <zg30>
transmitter freq.: 600.135401 MHz
time domain size: 32768 points
width: 12019.23 Hz = 20.0275 ppm = 0.366798 Hz/pt
number of scans: 61

freq. of 0 ppm: 600.130010 MHz
processed size: 32768 complex points
LB: 0.000 GF: 0.0000
Hz/cm: 101.981 ppm/cm: 0.16993

Figure S11. ^{1}H NMR ($CDCl_3$) spectrum of aliphatic part of **3**.

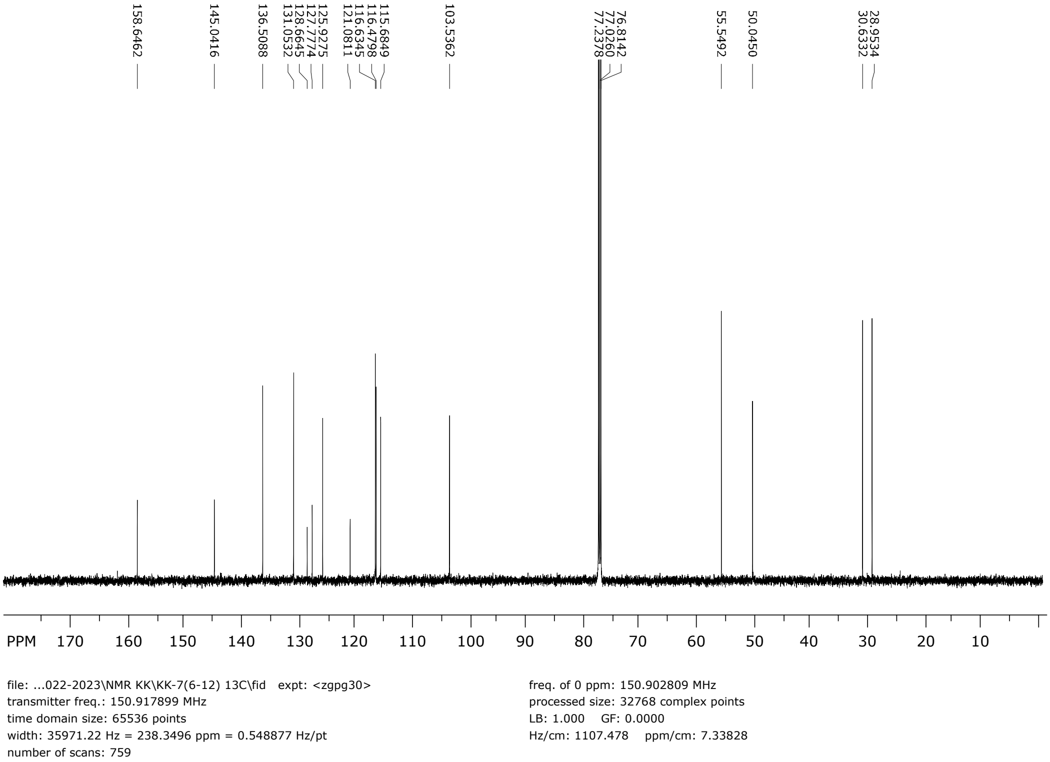

Figure S12. ^{13}C NMR ($CDCl_3$) spectrum of **3**.

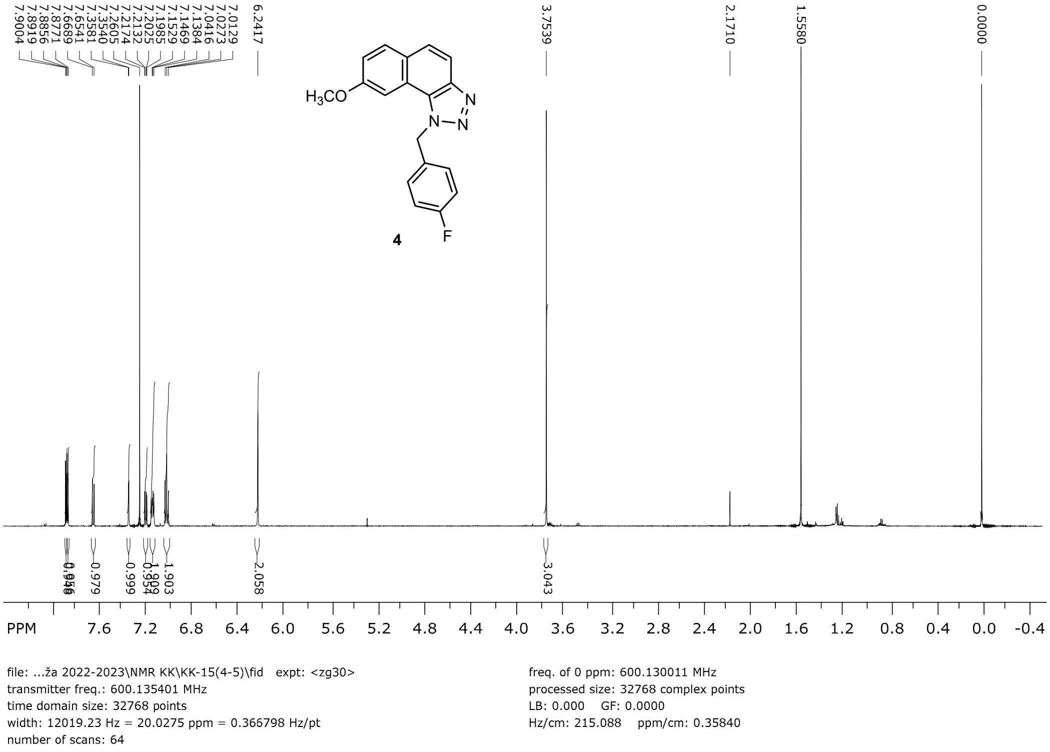

Figure S13. ^{1}H NMR ($CDCl_3$) spectrum of **4**.

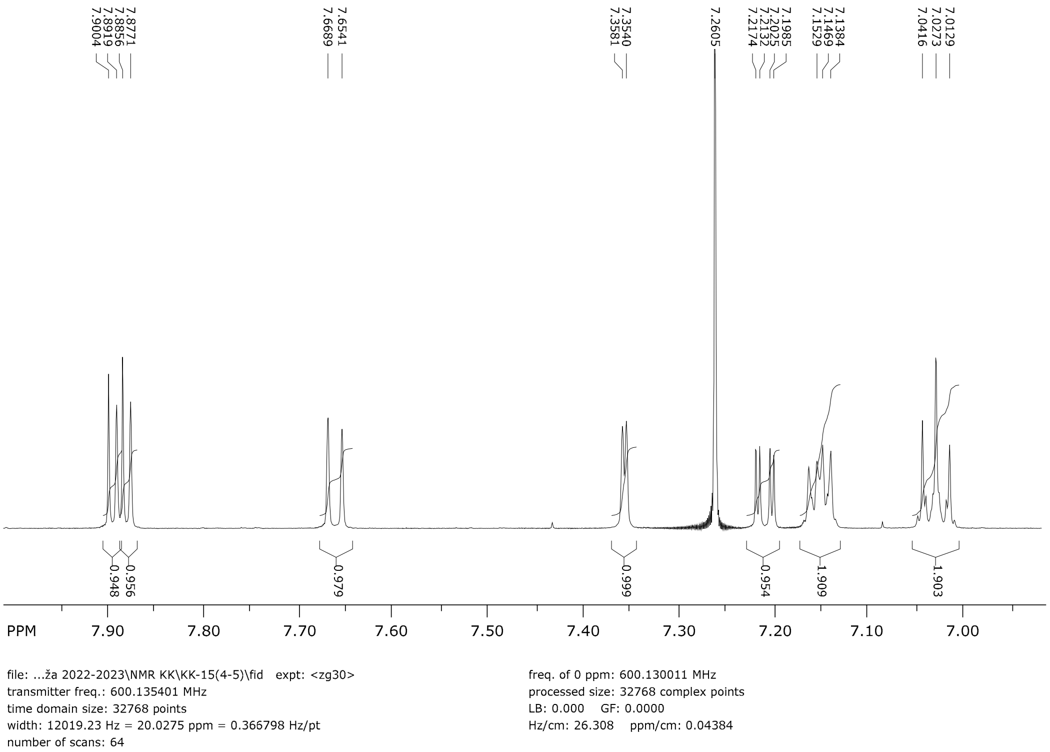

Figure S14. ^{1}H NMR ($CDCl_3$) spectrum of aromatic part of **4**.

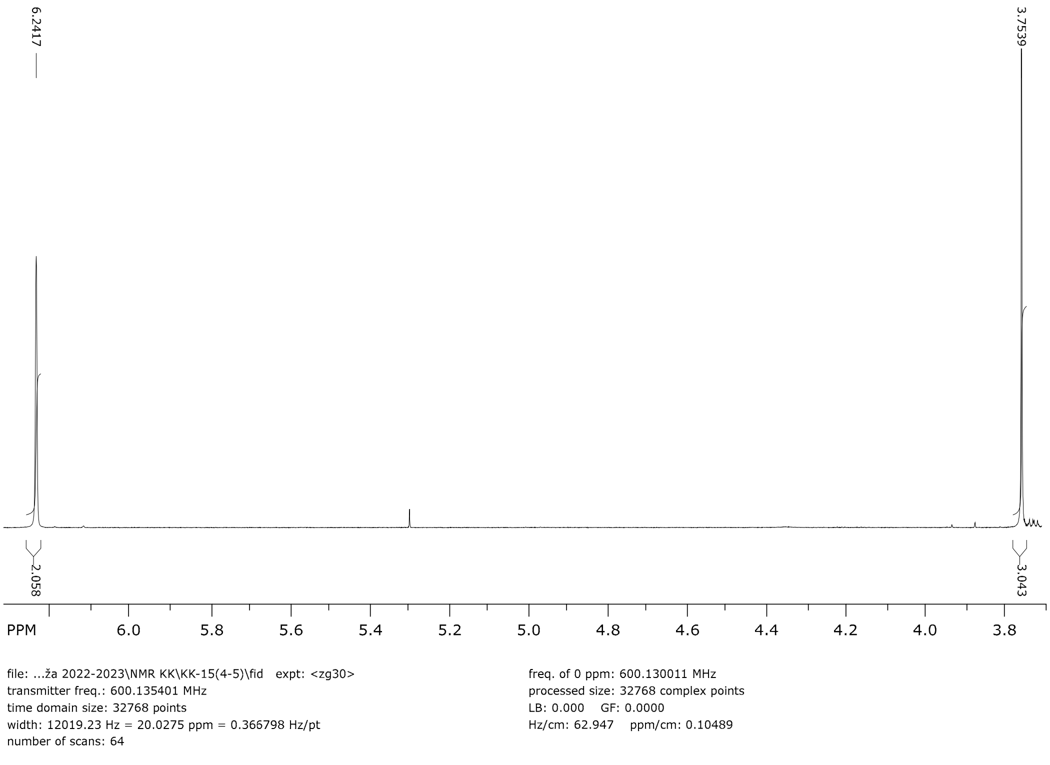

Figure S15. ^{1}H NMR ($CDCl_3$) spectrum of aliphatic part of **4**.

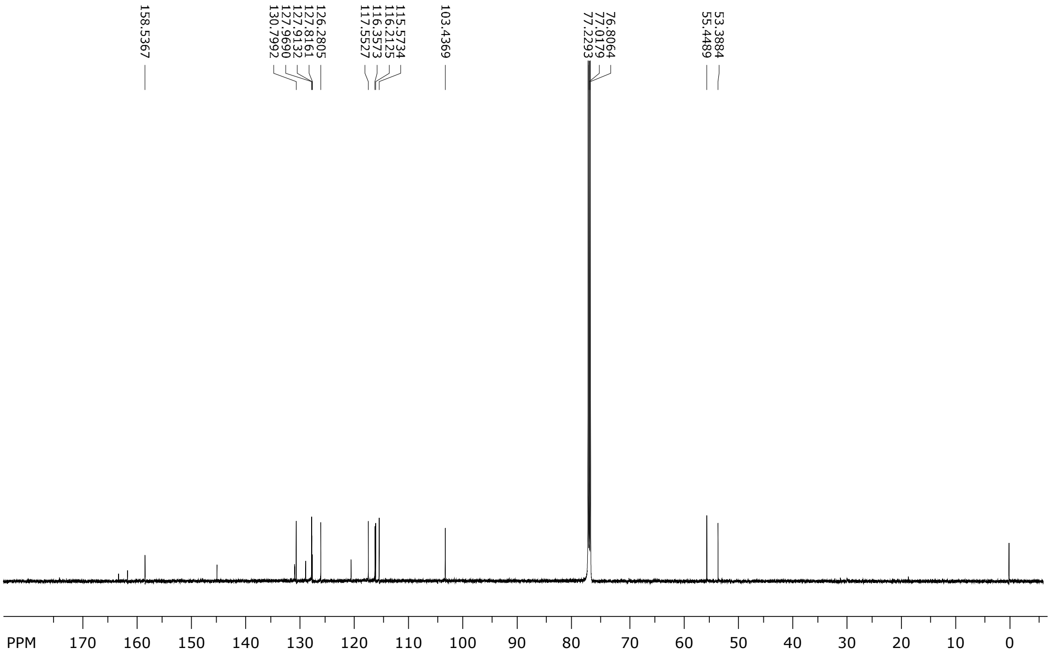

file: ...022-2023\NMR KK\KK-15(4-5) 13C\fid expt: <zgpg30>
transmitter freq.: 150.917899 MHz
time domain size: 65536 points
width: 35971.22 Hz = 238.3496 ppm = 0.548877 Hz/pt
number of scans: 20480

freq. of 0 ppm: 150.902808 MHz
processed size: 32768 complex points
LB: 1.000 GF: 0.0000
Hz/cm: 1151.872 ppm/cm: 7.63244

Figure S16. ^{13}C NMR ($CDCl_3$) spectrum of **4**.

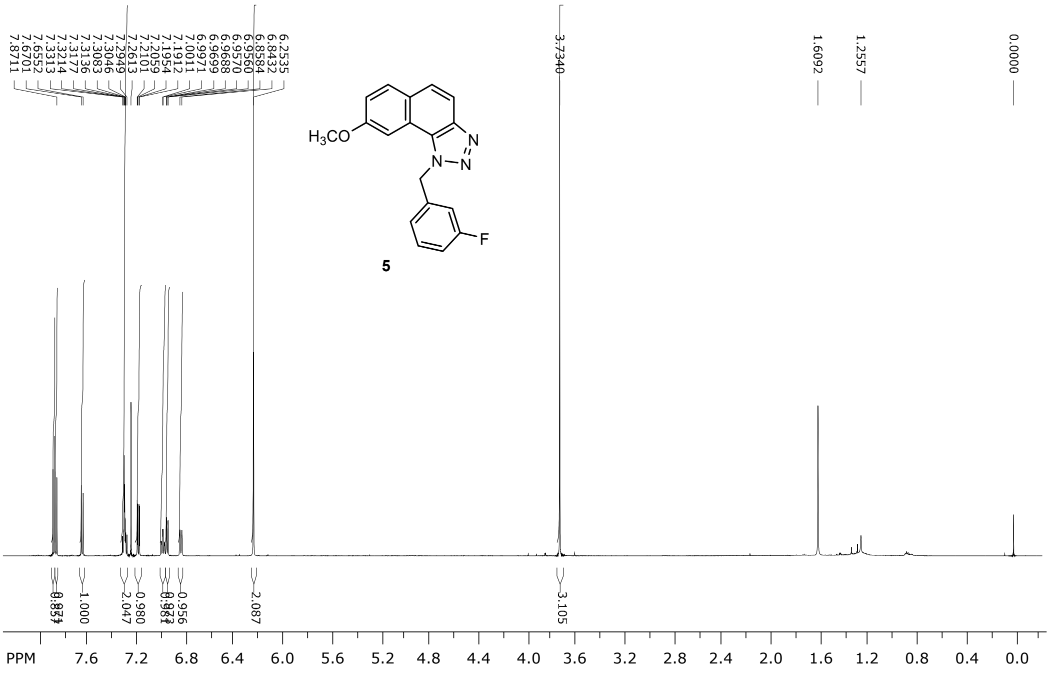

file: ...dža 2022-2023\NMR KK\MD-390(1)\fid expt: <zg30>
transmitter freq.: 600.135401 MHz
time domain size: 32768 points
width: 12019.23 Hz = 20.0275 ppm = 0.366798 Hz/pt
number of scans: 64

freq. of 0 ppm: 600.130010 MHz
processed size: 32768 complex points
LB: 0.000 GF: 0.0000
Hz/cm: 205.287 ppm/cm: 0.34207

Figure S17. ^{1}H NMR ($CDCl_3$) spectrum of **5**.

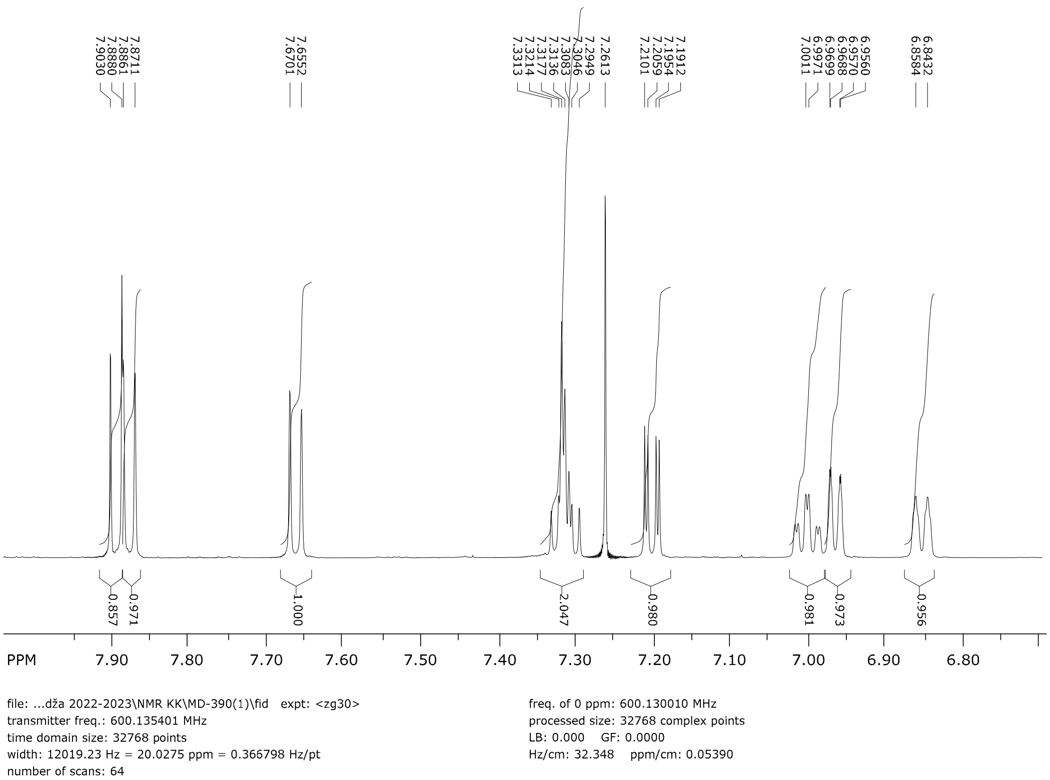

Figure S18. ^{1}H NMR ($CDCl_3$) spectrum of aromatic part of **5**.

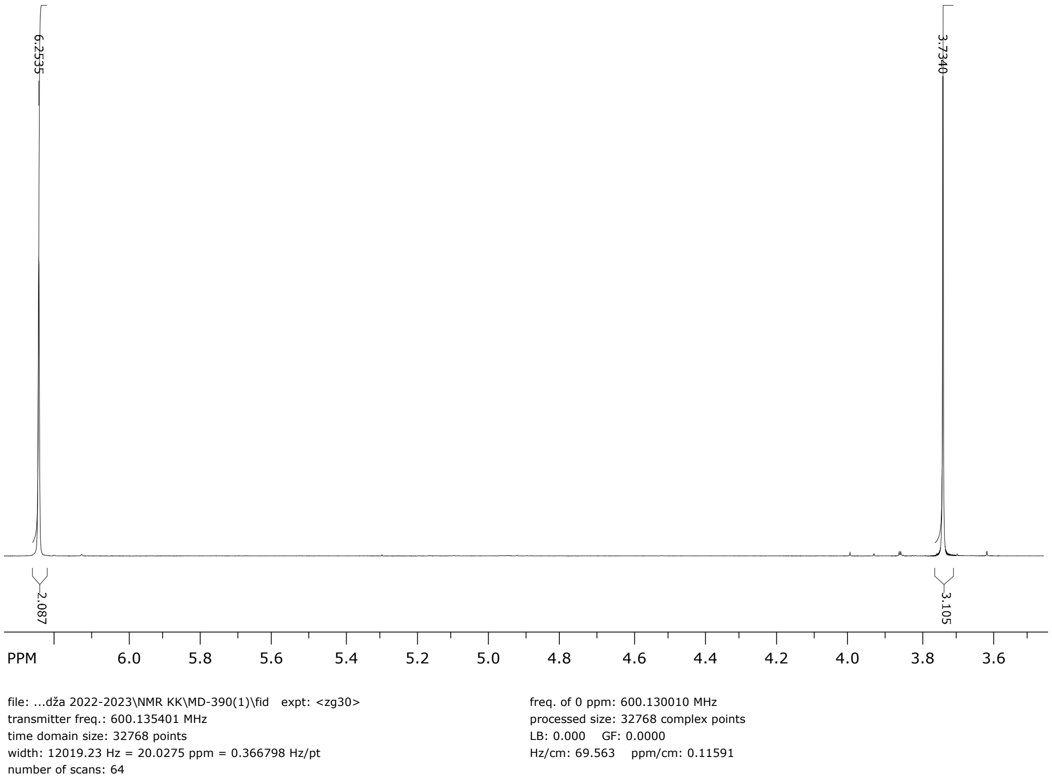

Figure S19. ^{1}H NMR ($CDCl_3$) spectrum of aliphatic part of **5**.

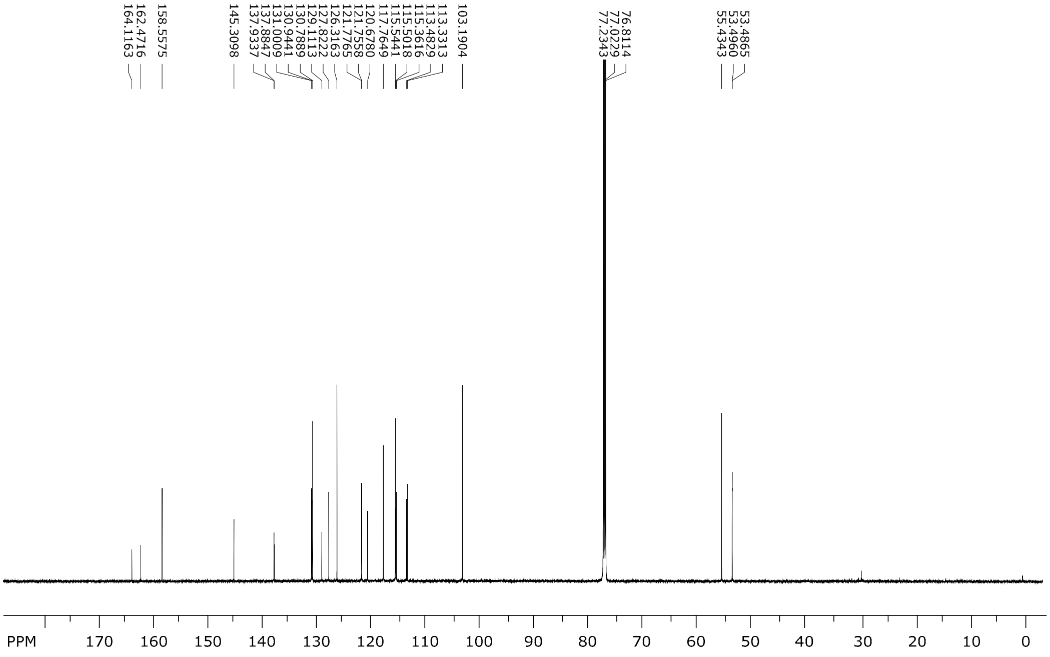

file: ...2022-2023\NMR KK\MD-390(1) 13C\fid expt: <zgpg30>
transmitter freq.: 150.917899 MHz
time domain size: 65536 points
width: 35971.22 Hz = 238.3496 ppm = 0.548877 Hz/pt
number of scans: 20480

freq. of 0 ppm: 150.902809 MHz
processed size: 32768 complex points
LB: 1.000 GF: 0.0000
Hz/cm: 1155.836 ppm/cm: 7.65870

Figure S20. ^{13}C NMR (CDCl$_3$) spectrum of **5**.

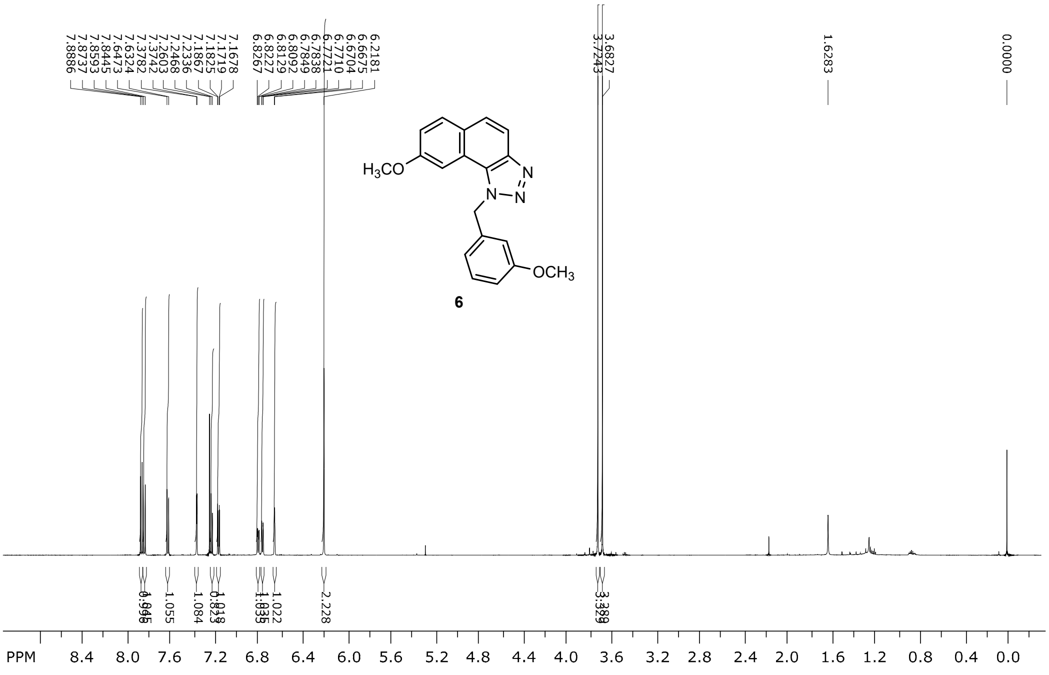

file: ...a 2022-2023\NMR KK\MD-385(1-4)\fid expt: <zg30>
transmitter freq.: 600.135401 MHz
time domain size: 32768 points
width: 12019.23 Hz = 20.0275 ppm = 0.366798 Hz/pt
number of scans: 64

freq. of 0 ppm: 600.130011 MHz
processed size: 32768 complex points
LB: 0.000 GF: 0.0000
Hz/cm: 227.008 ppm/cm: 0.37826

Figure S21. ^{1}H NMR (CDCl$_3$) spectrum of **6**.

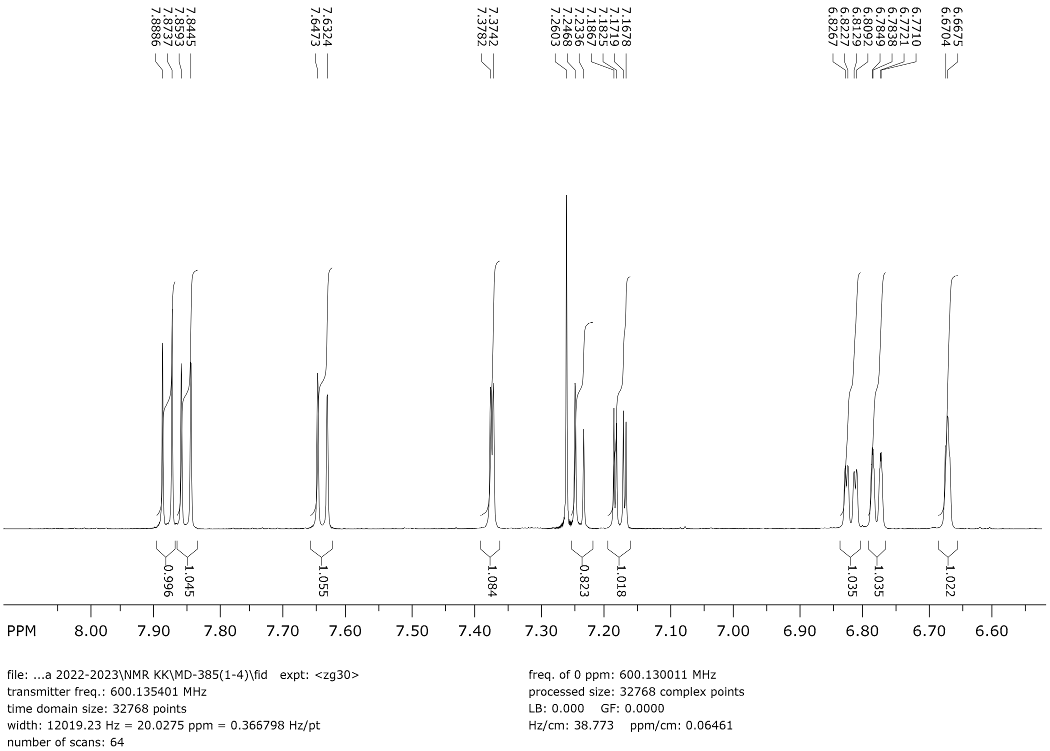

Figure S22. ^{1}H NMR ($CDCl_3$) spectrum of aromatic part of **6**.

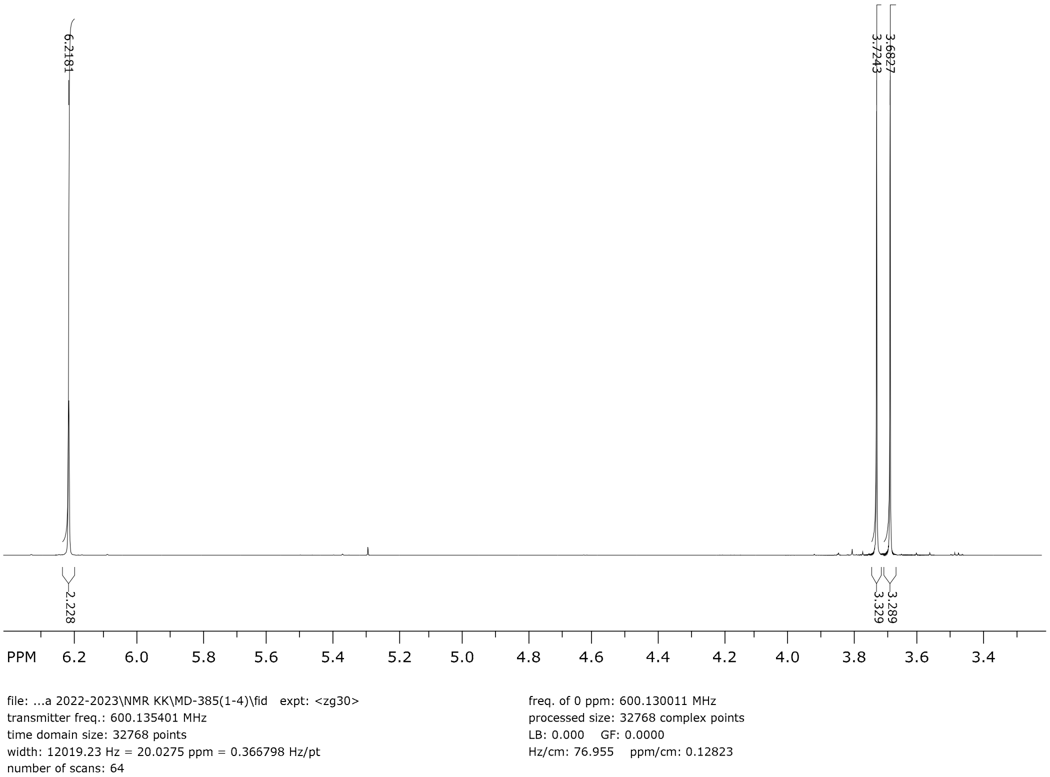

Figure S23. ^{1}H NMR ($CDCl_3$) spectrum of aliphatic part of **6**.

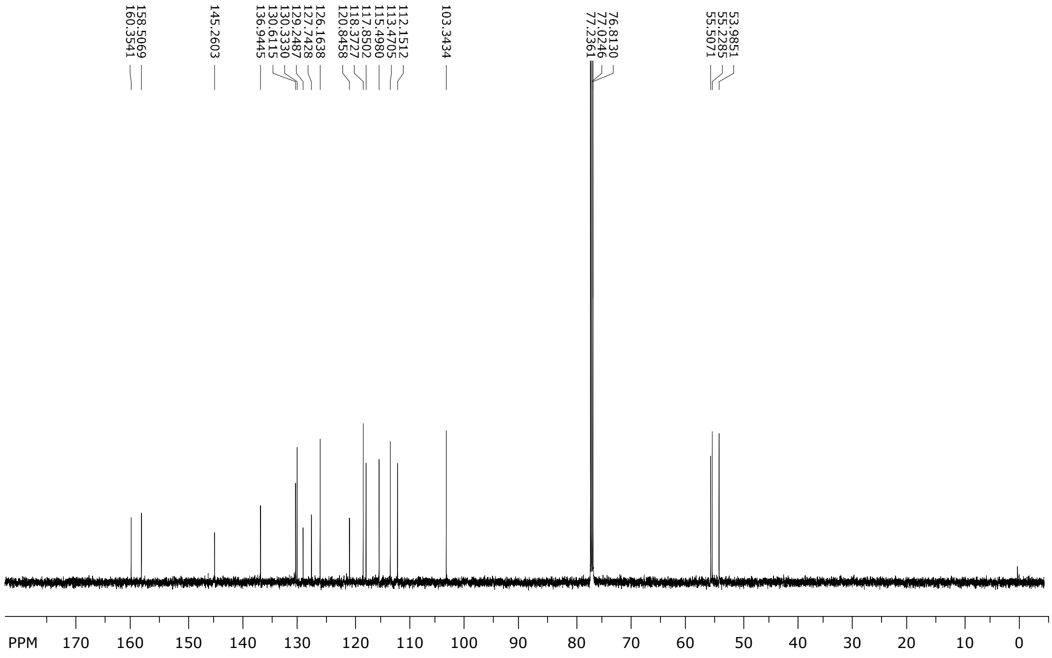

file: ...22-2023\NMR KK\MD-385(1-4) 13C\fid expt: <zgpg30>
transmitter freq.: 150.917899 MHz
time domain size: 65536 points
width: 35971.22 Hz = 238.3496 ppm = 0.548877 Hz/pt
number of scans: 321

freq. of 0 ppm: 150.902809 MHz
processed size: 32768 complex points
LB: 1.000 GF: 0.0000
Hz/cm: 1135.224 ppm/cm: 7.52213

Figure S24. ^{13}C NMR ($CDCl_3$) spectrum of **6**.

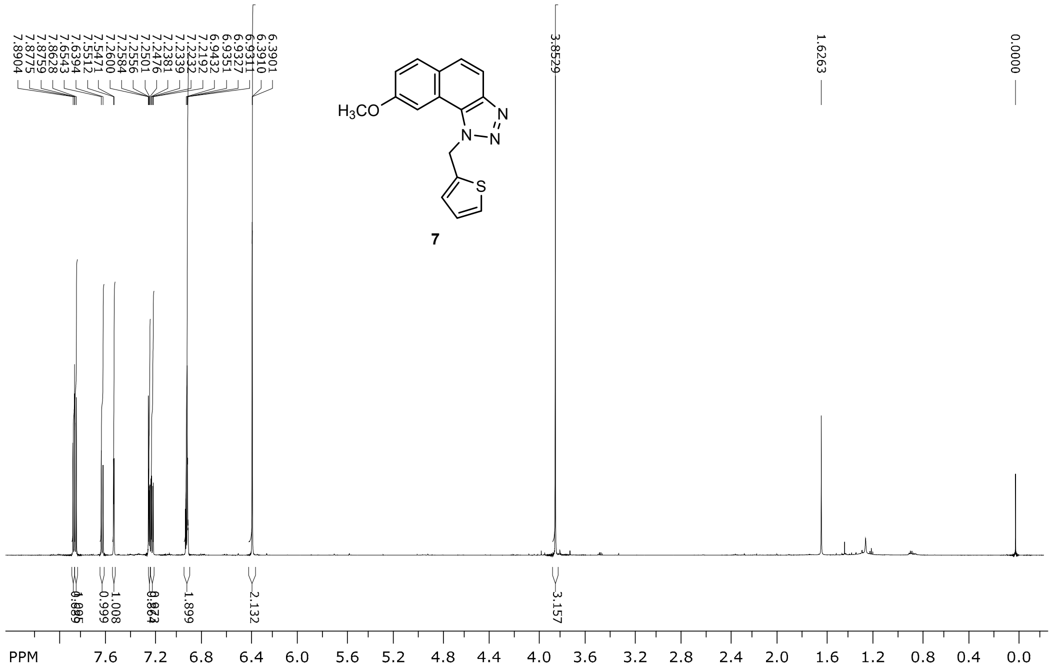

file: ...22-2023\NMR KK\KK-17(1-5)(1-2)\fid expt: <zg30>
transmitter freq.: 600.135401 MHz
time domain size: 32768 points
width: 12019.23 Hz = 20.0275 ppm = 0.366798 Hz/pt
number of scans: 64

freq. of 0 ppm: 600.130011 MHz
processed size: 32768 complex points
LB: 0.000 GF: 0.0000
Hz/cm: 208.731 ppm/cm: 0.34781

Figure S25. ^{1}H NMR ($CDCl_3$) spectrum of **7**.

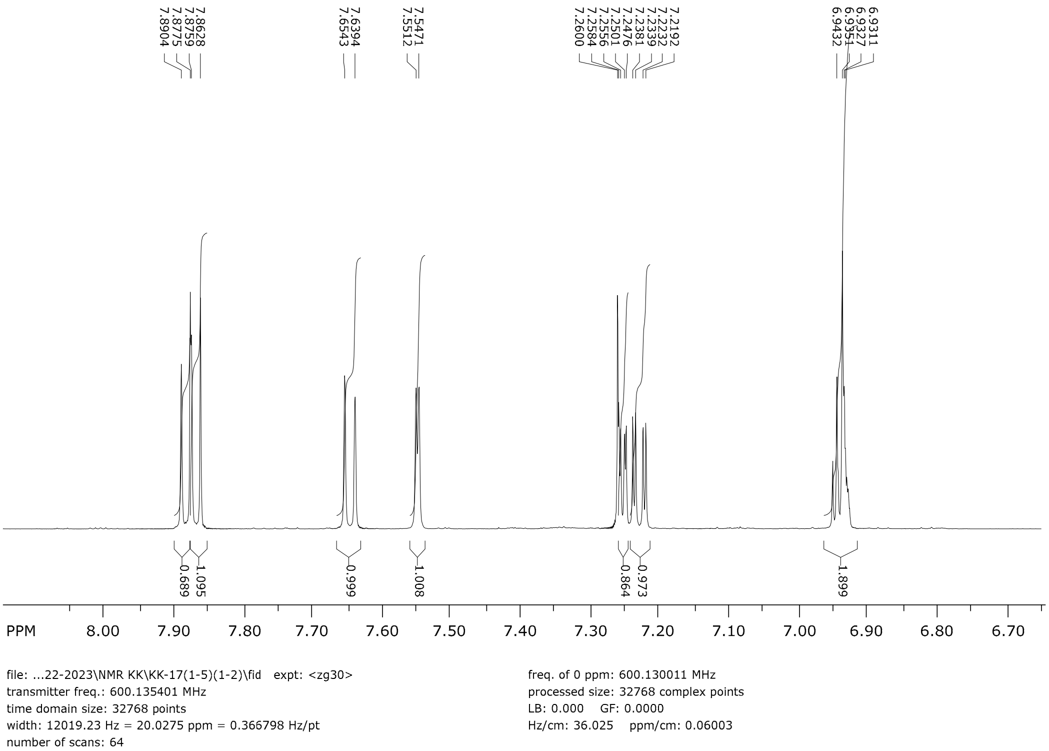

Figure S26. ^{1}H NMR ($CDCl_3$) spectrum of aromatic part of **7**.

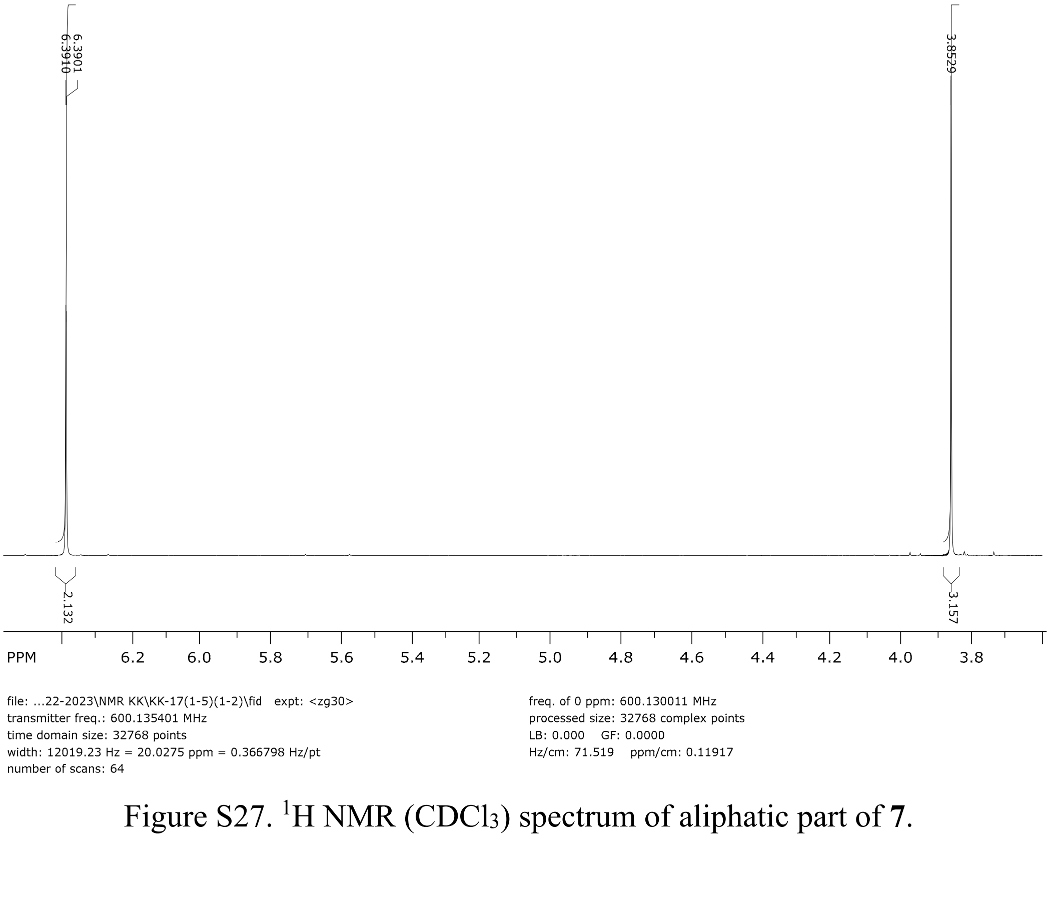

Figure S27. ^{1}H NMR ($CDCl_3$) spectrum of aliphatic part of **7**.

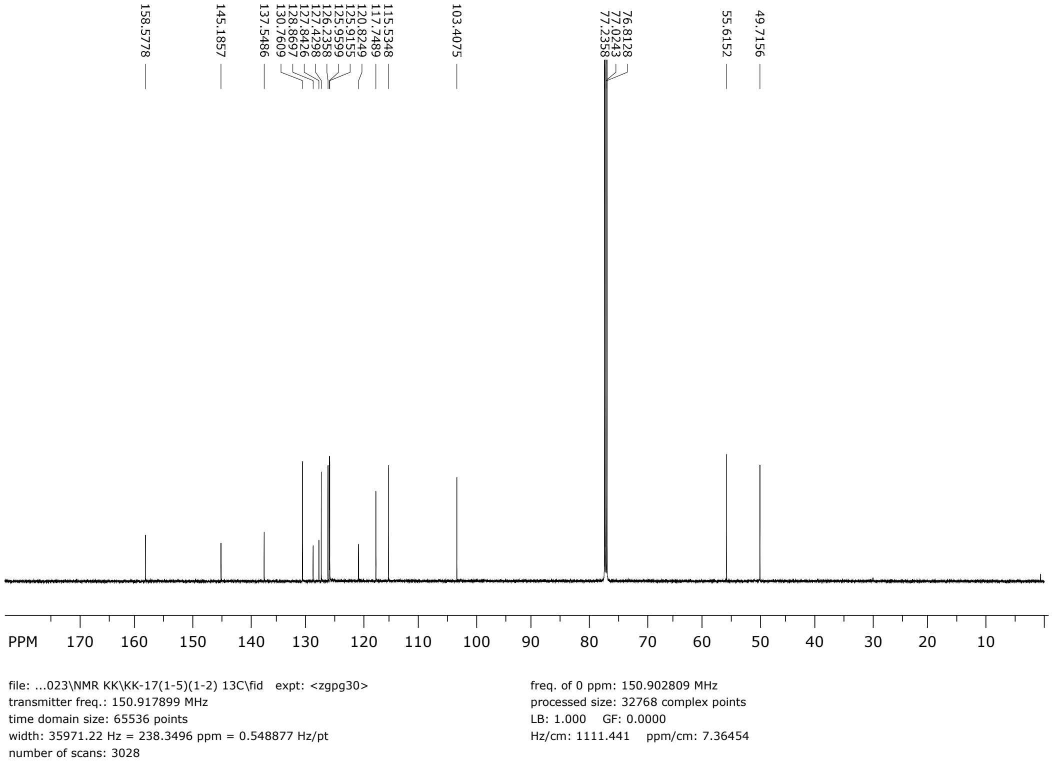

Figure S28. ^{13}C NMR (CDCl$_3$) spectrum of **7**.

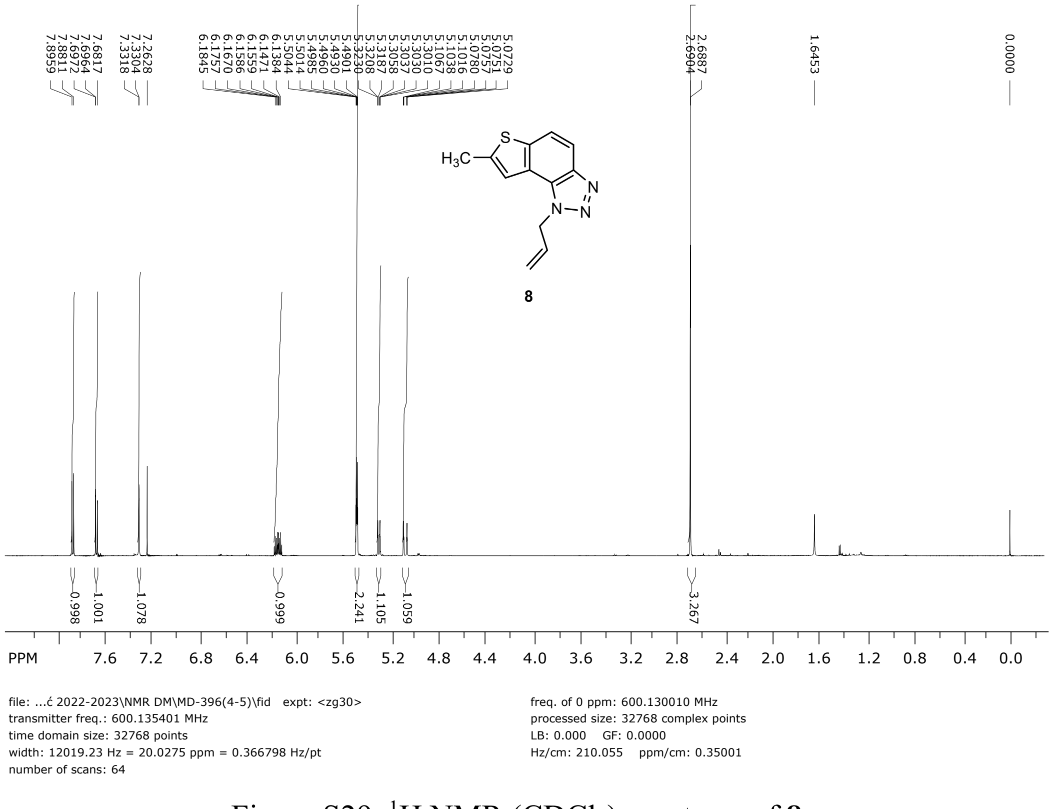

Figure S29. ^{1}H NMR (CDCl$_3$) spectrum of **8**.

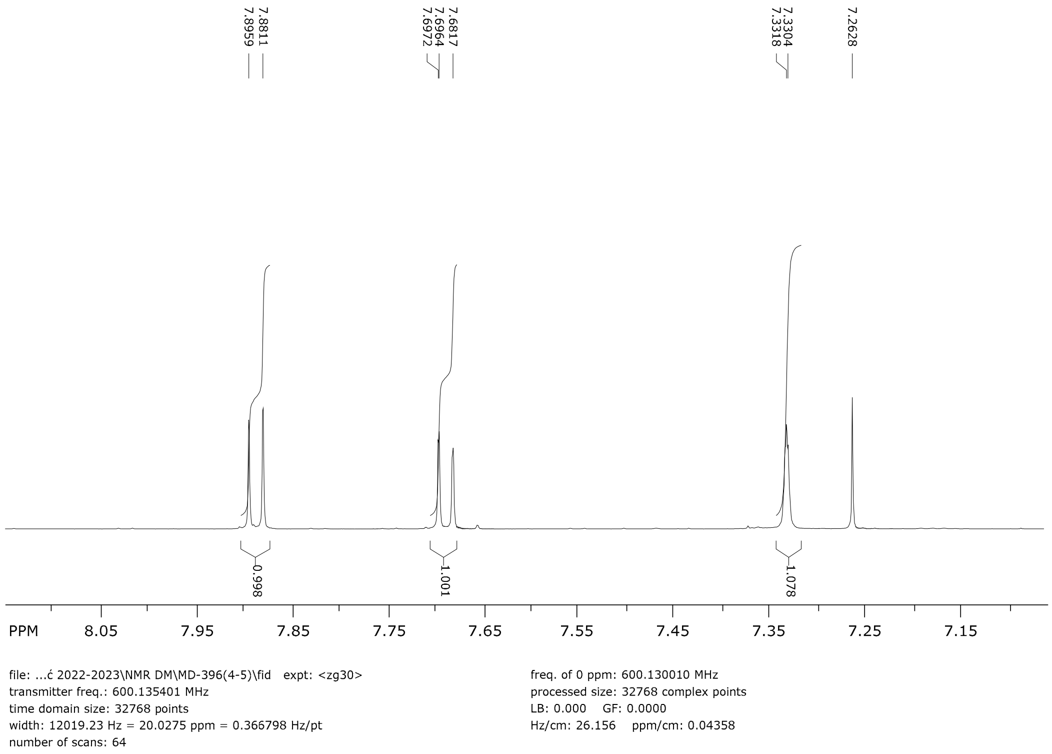

Figure S30. ^{1}H NMR ($CDCl_3$) spectrum of aromatic part of **8**.

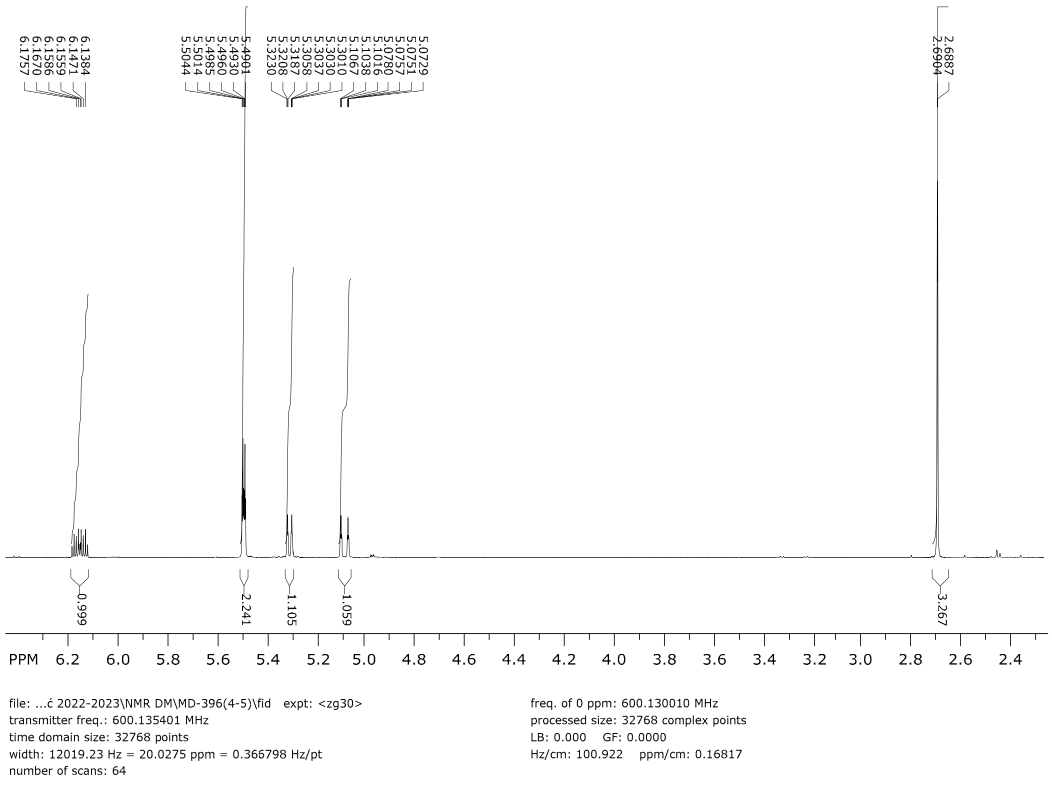

Figure S31. ^{1}H NMR ($CDCl_3$) spectrum of aliphatic part of **8**.

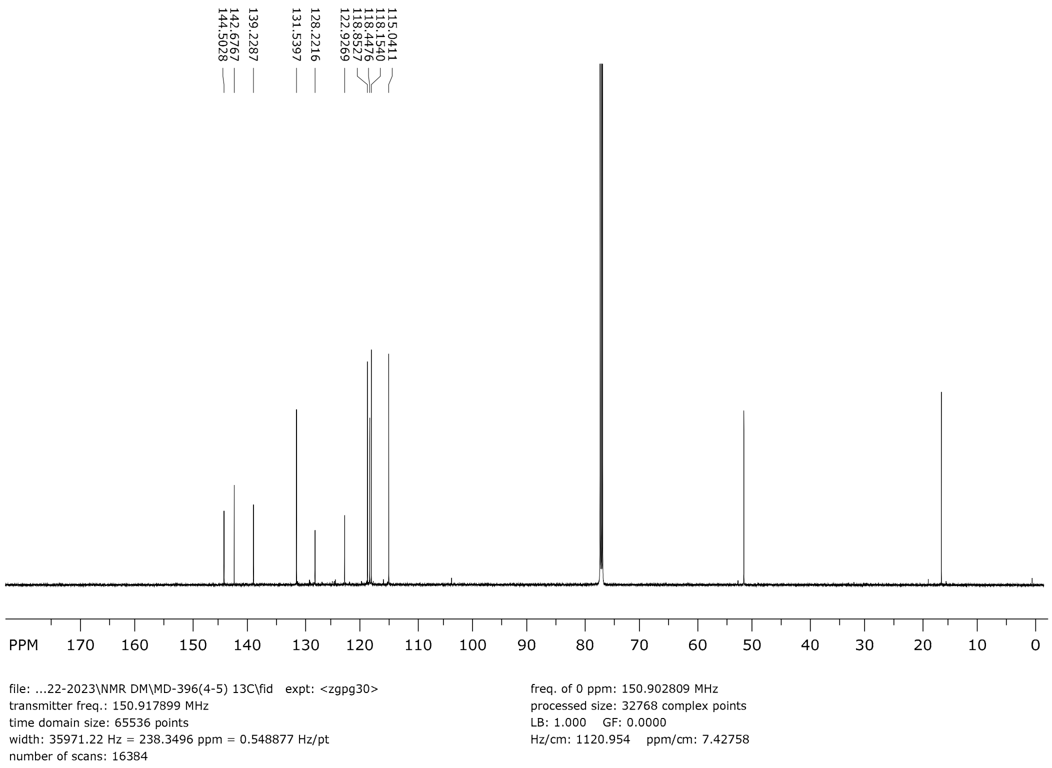

Figure S32. ^{13}C NMR ($CDCl_3$) spectrum of **8**.

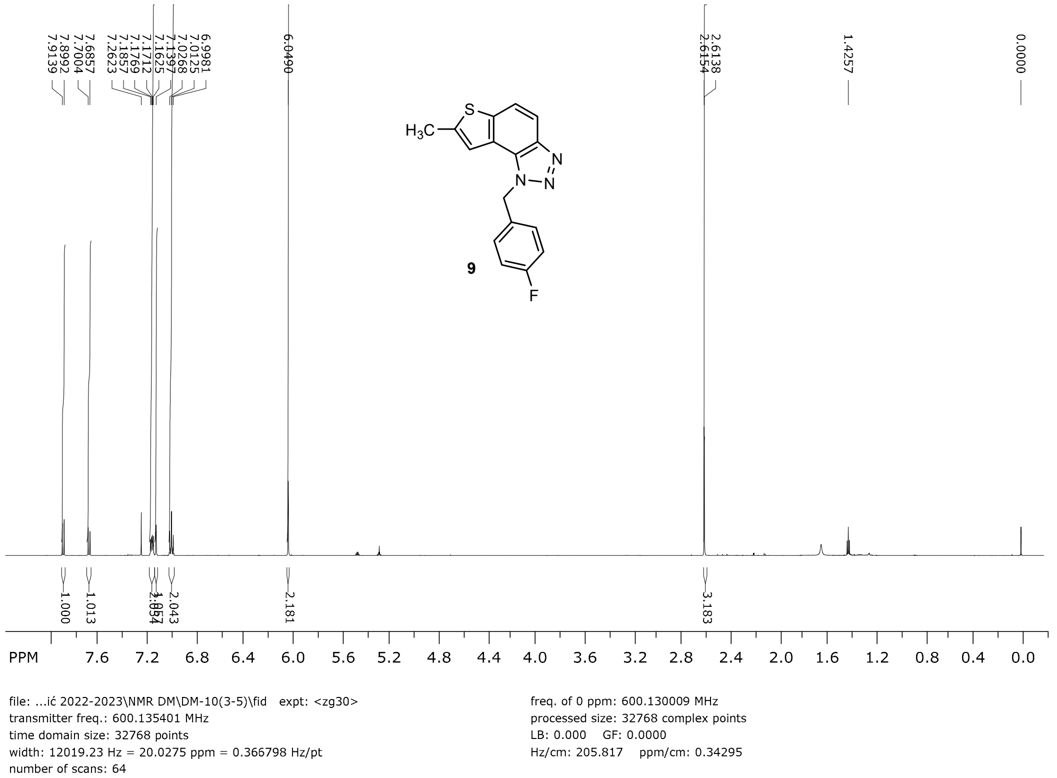

Figure S33. ^{1}H NMR ($CDCl_3$) spectrum of **9**.

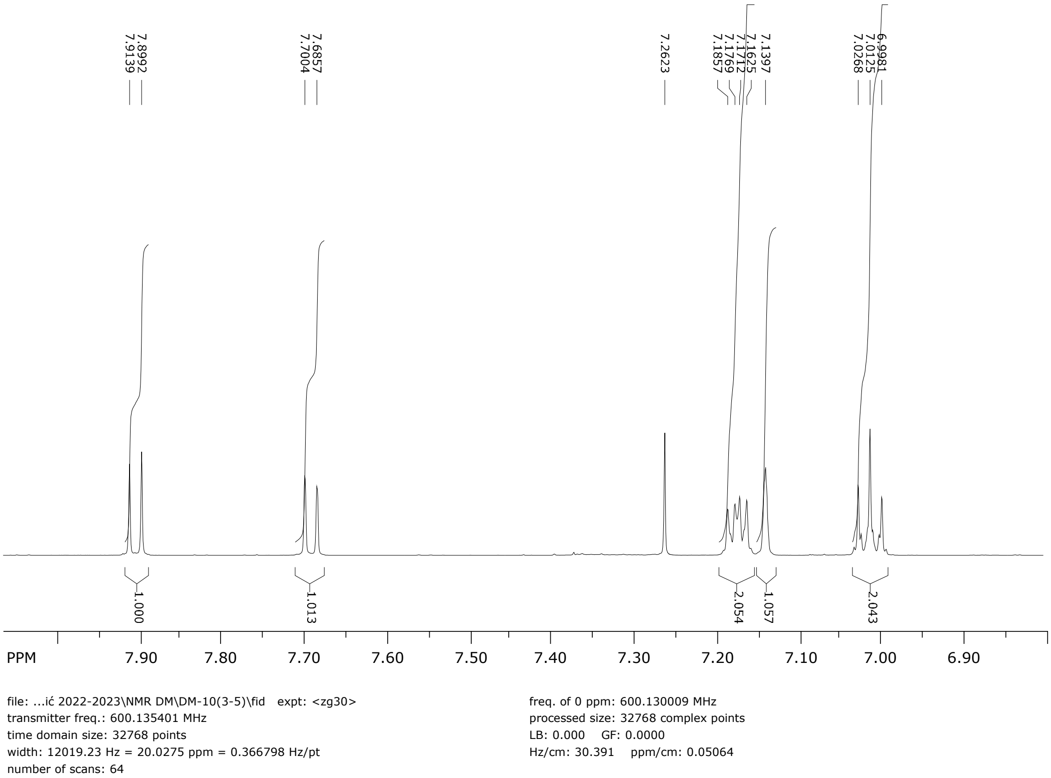

Figure S34. ^{1}H NMR (CDCl$_3$) spectrum of aromatic part of **9**.

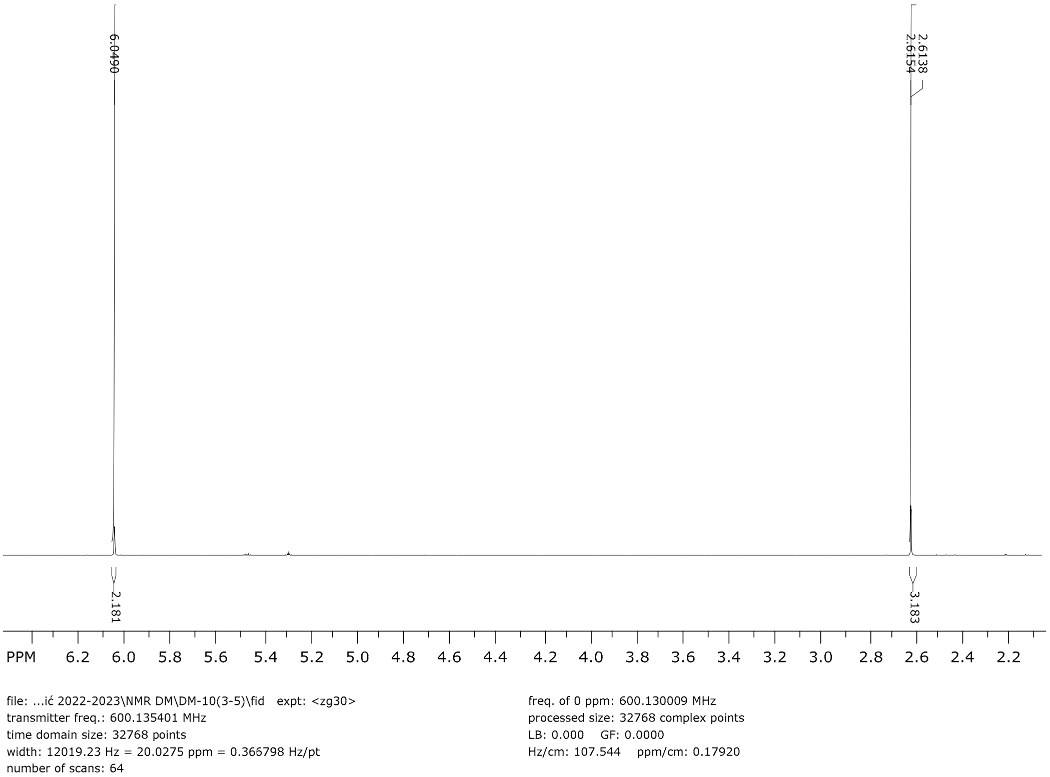

Figure S35. ^{1}H NMR (CDCl$_3$) spectrum of aliphatic part of **9**.

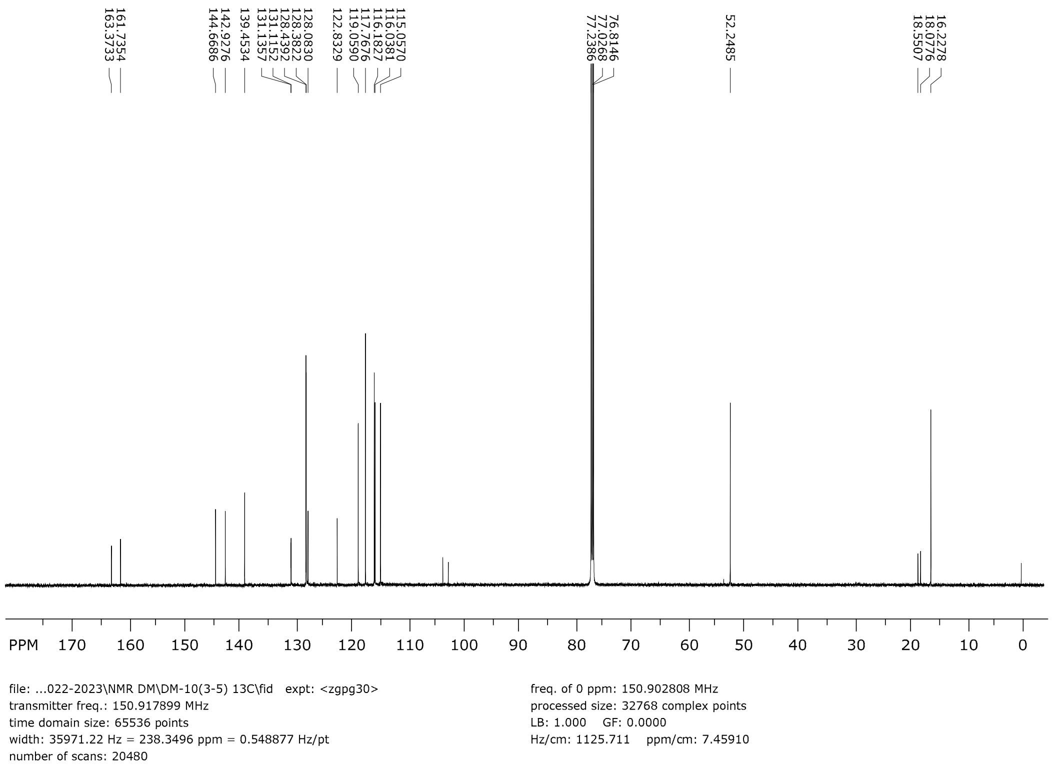

Figure S36. ^{13}C NMR (CDCl$_3$) spectrum of **9**.

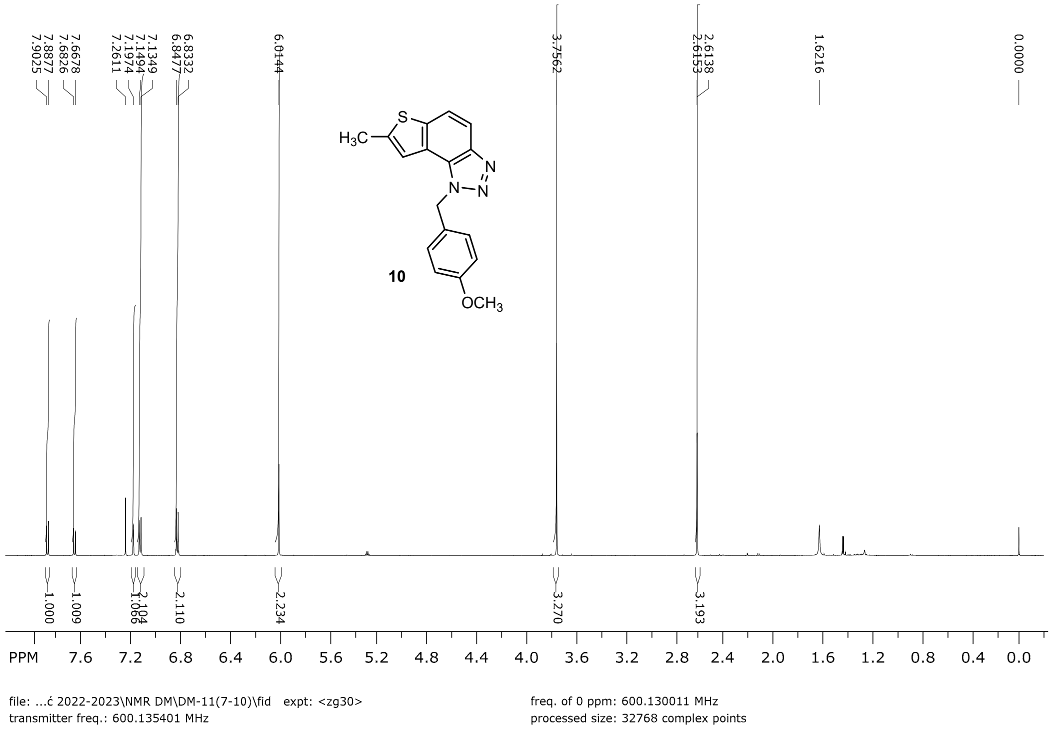

Figure S37. ^{1}H NMR ($CDCl_3$) spectrum of **10**.

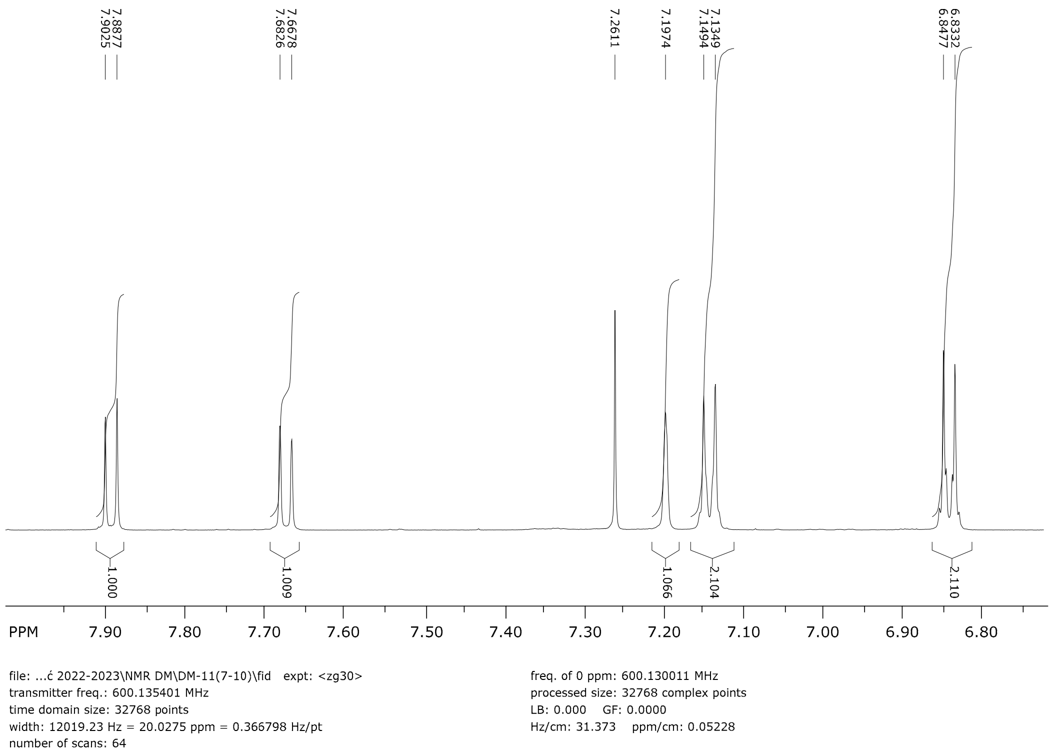

Figure S38. ^{1}H NMR ($CDCl_3$) spectrum of aromatic part of **10**.

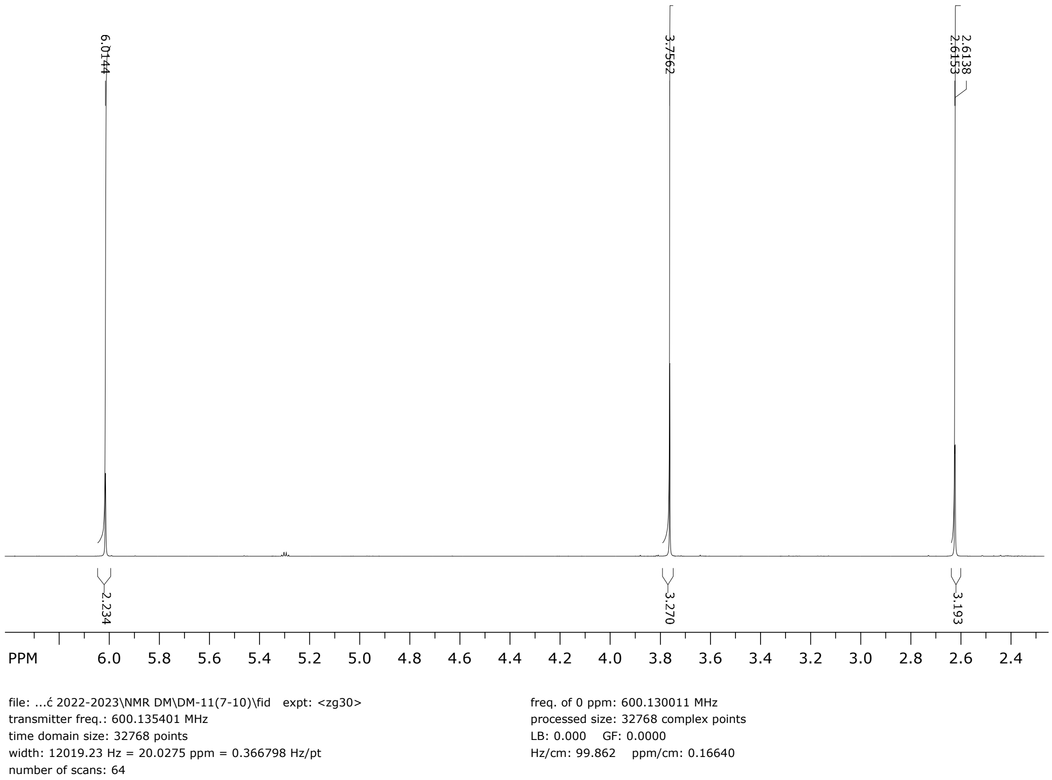

Figure S39. ^{1}H NMR ($CDCl_3$) spectrum of aliphatic part of **10**.

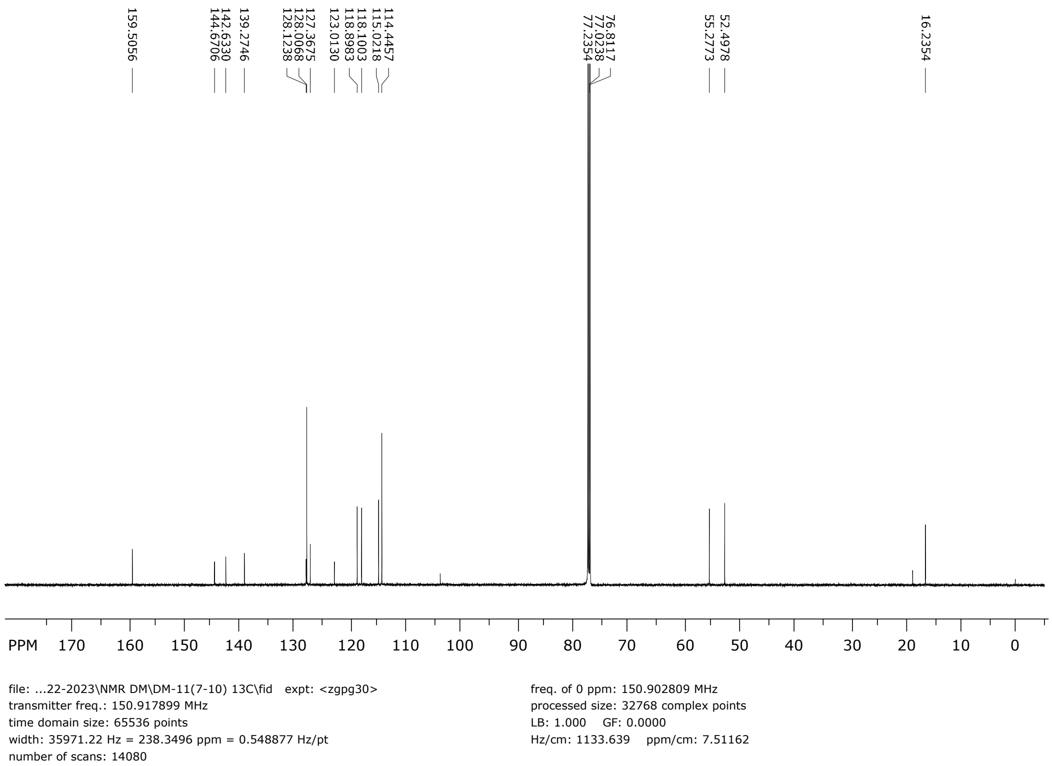

Figure S40. ^{13}C NMR ($CDCl_3$) spectrum of **10**.

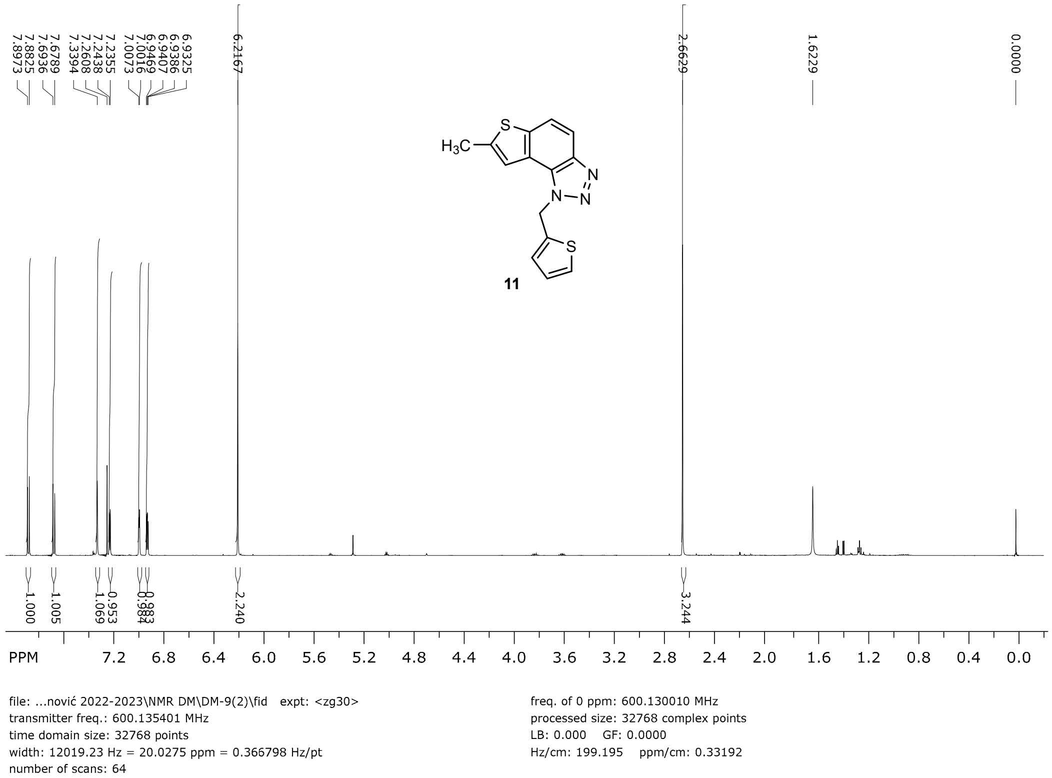

Figure S41. ^{1}H NMR ($CDCl_3$) spectrum of **11**.

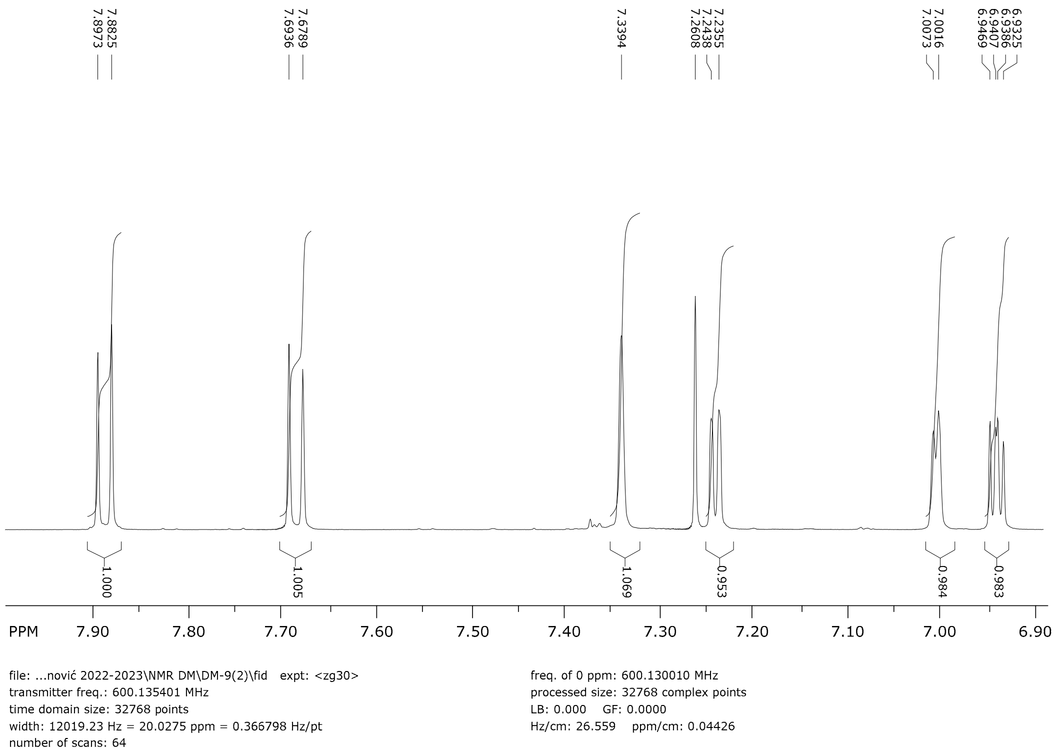

Figure S42. ^{1}H NMR ($CDCl_3$) spectrum of aromatic part of **11**.

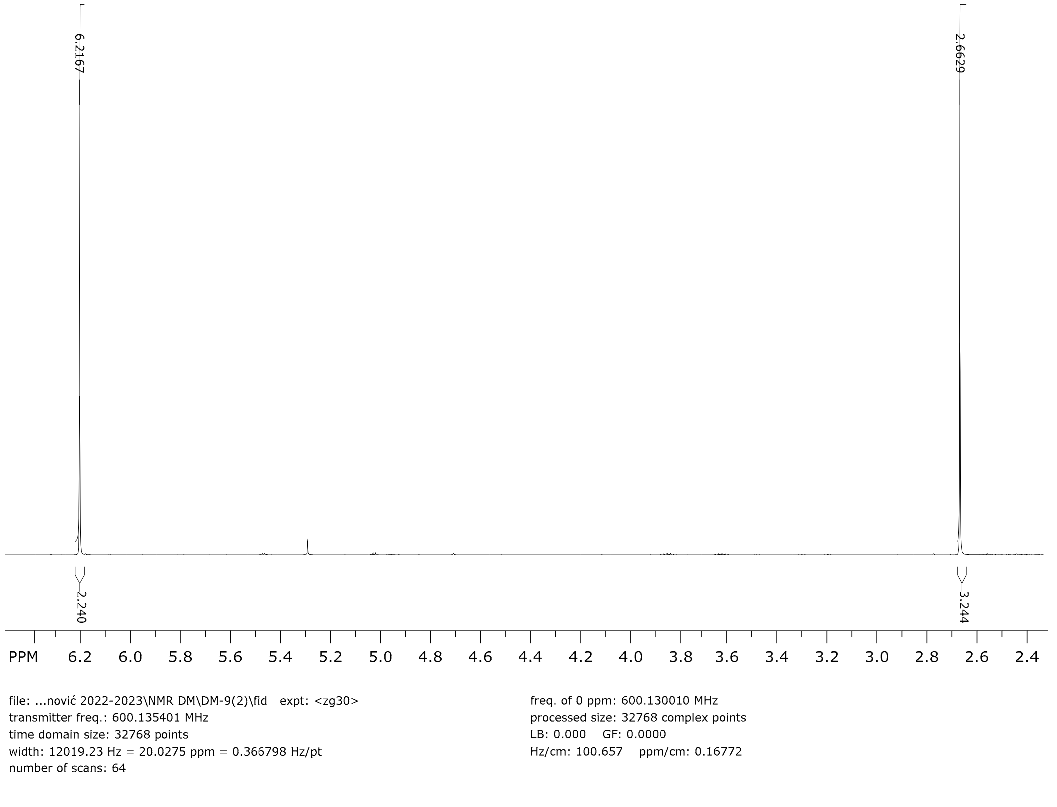

Figure S43. ^{1}H NMR (CDCl3) spectrum of aliphatic part of **11**.

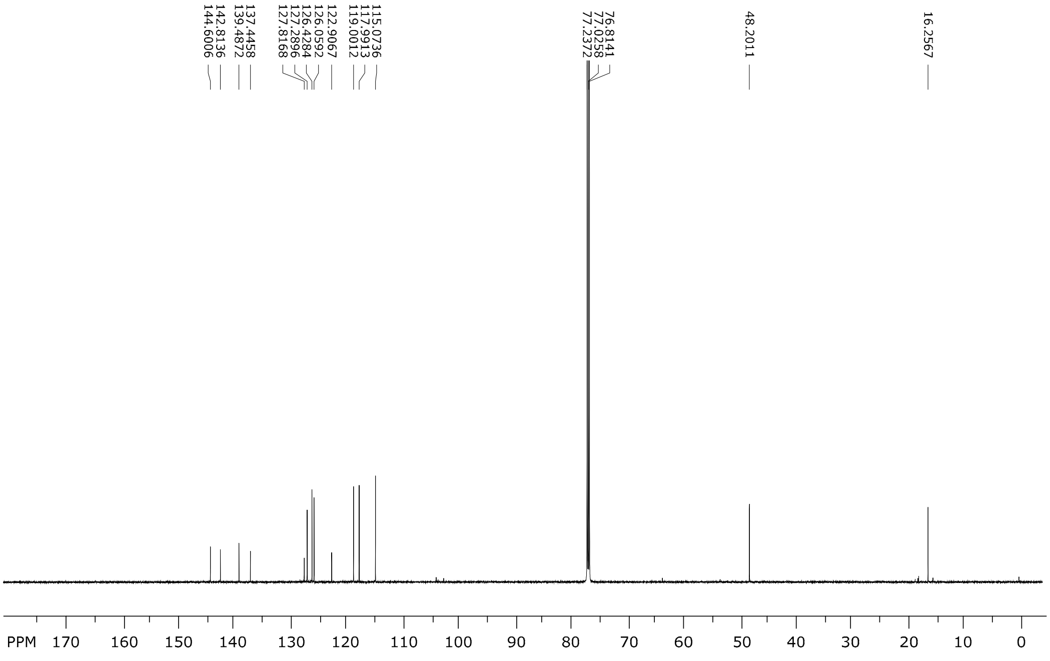

file: ...ć 2022-2023\NMR DM\DM-9(2) 13C\fid expt: <zgpg30>
transmitter freq.: 150.917899 MHz
time domain size: 65536 points
width: 35971.22 Hz = 238.3496 ppm = 0.548877 Hz/pt
number of scans: 20480

freq. of 0 ppm: 150.902808 MHz
processed size: 32768 complex points
LB: 1.000 GF: 0.0000
Hz/cm: 1121.747 ppm/cm: 7.43283

Figure S44. ^{13}C NMR (CDCl3) spectrum of **11**.

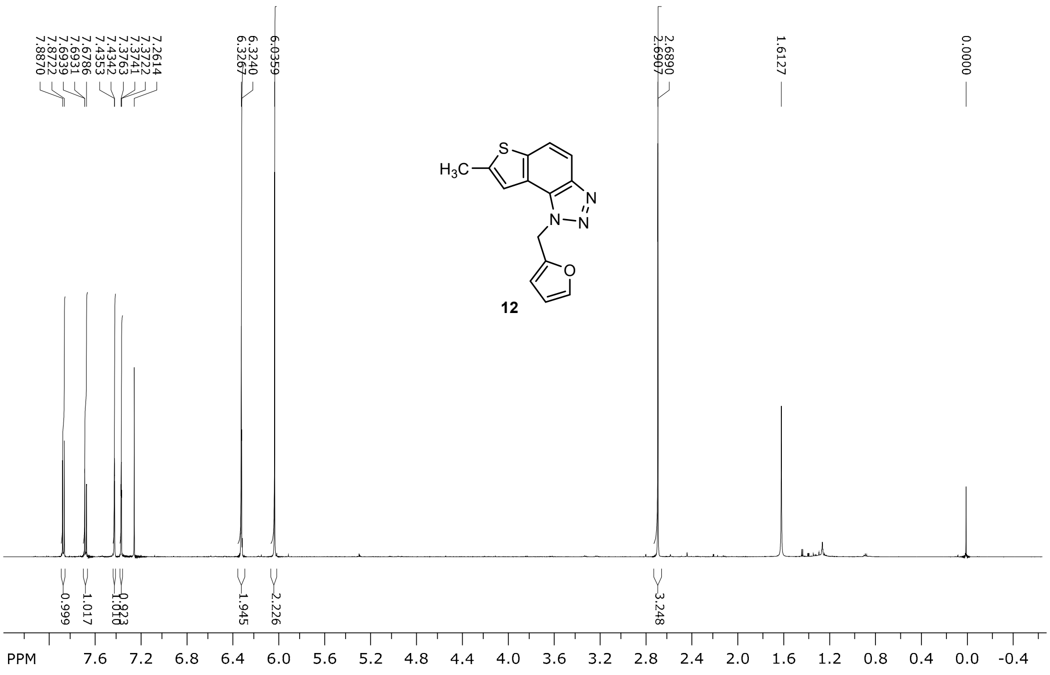

file: ...ć 2022-2023\NMR DM\DM-12(9-11)\fid expt: <zg30>
transmitter freq.: 600.135401 MHz
time domain size: 32768 points
width: 12019.23 Hz = 20.0275 ppm = 0.366798 Hz/pt
number of scans: 64

freq. of 0 ppm: 600.130010 MHz
processed size: 32768 complex points
LB: 0.000 GF: 0.0000
Hz/cm: 217.737 ppm/cm: 0.36281

Figure S45. ^{1}H NMR ($CDCl_3$) spectrum of **12**.

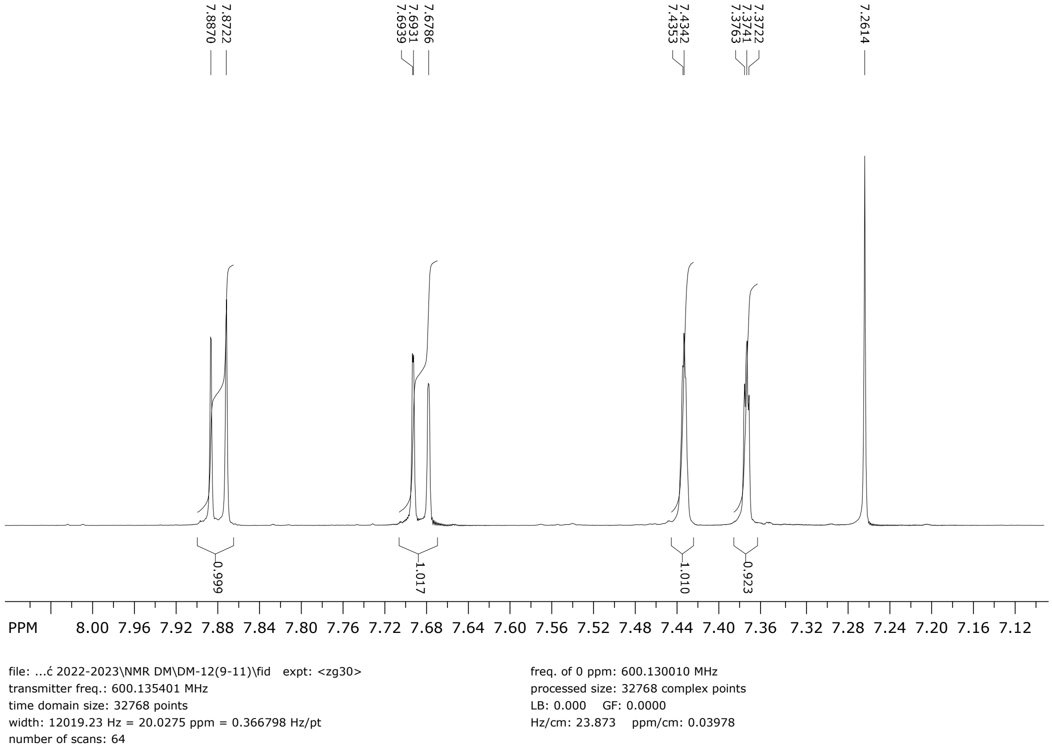

Figure S46. ^{1}H NMR ($CDCl_3$) spectrum of aromatic part of **12**.

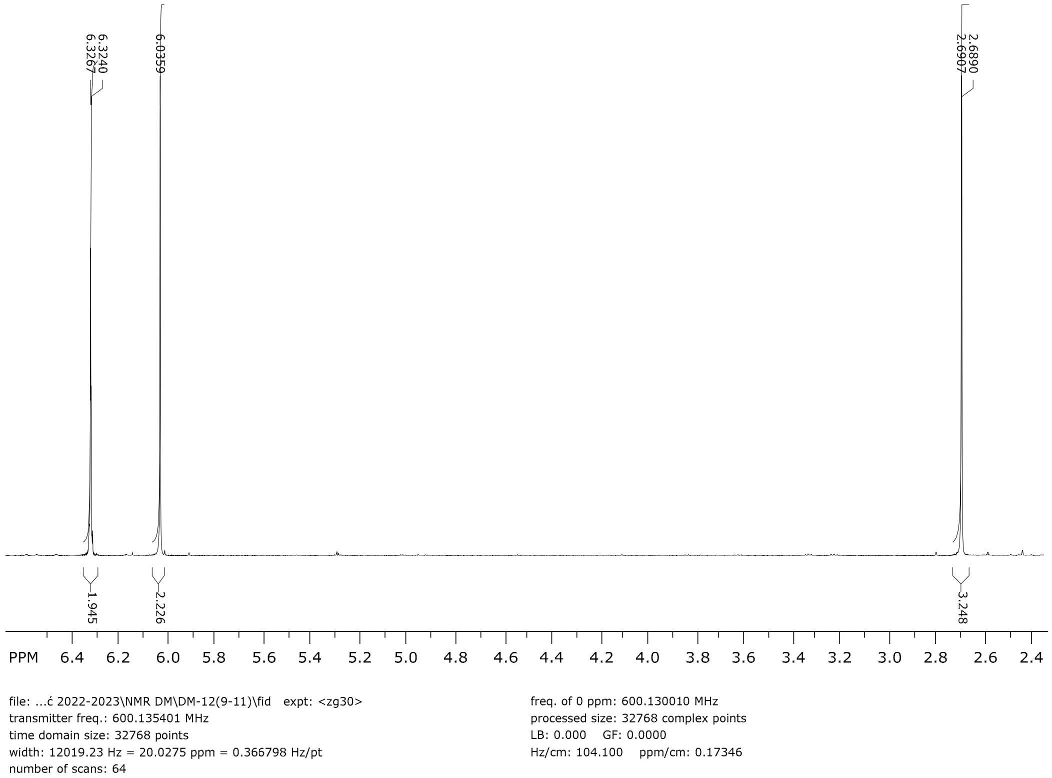

Figure S47. ^{1}H NMR (CDCl$_3$) spectrum of aliphatic part of **12**.

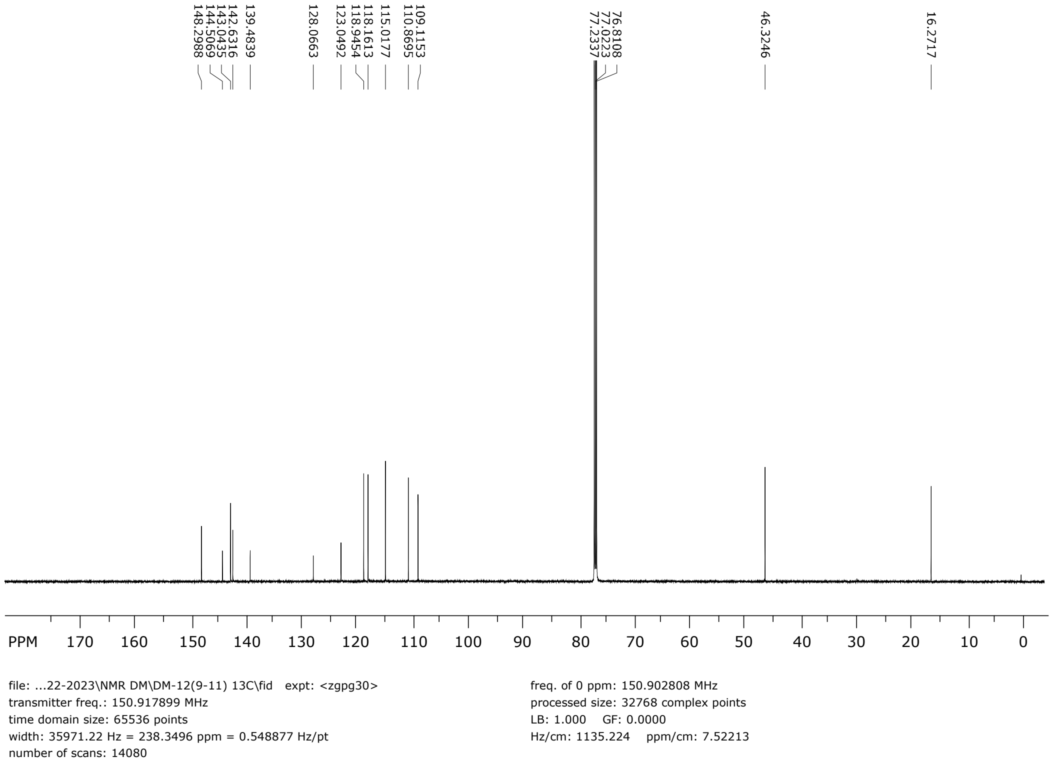

Figure S48. ^{13}C NMR (CDCl$_3$) spectrum of **12**.

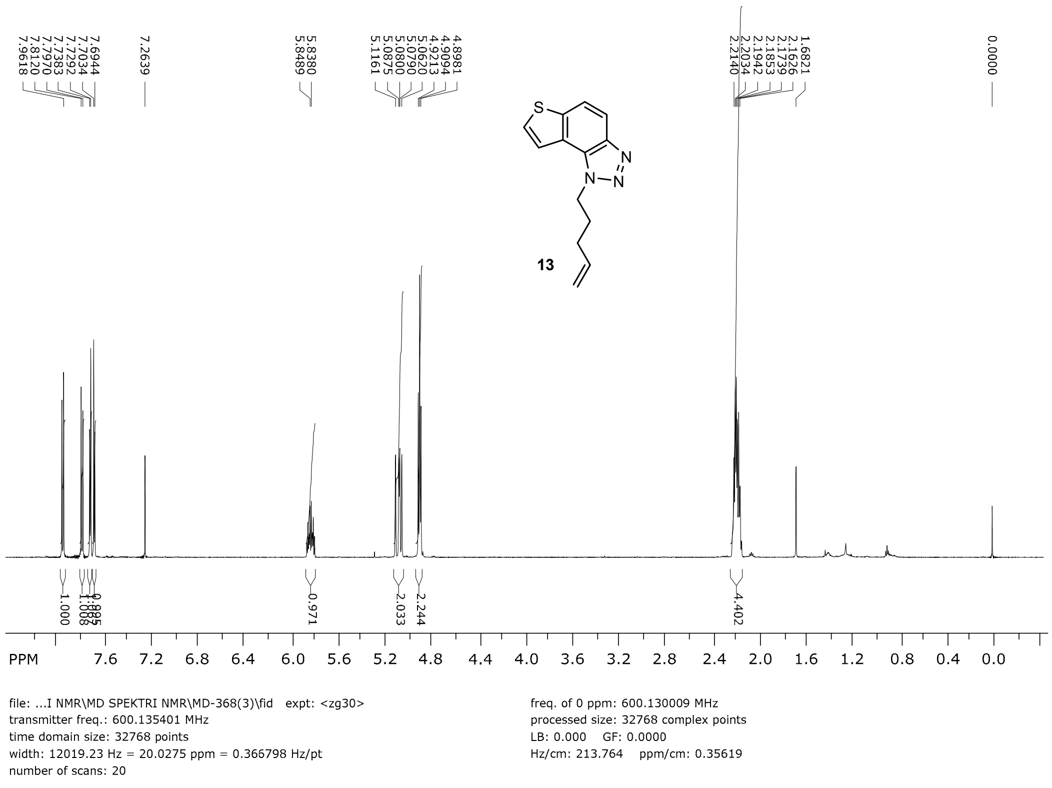

Figure S49. ^{1}H NMR ($CDCl_3$) spectrum of **13**.

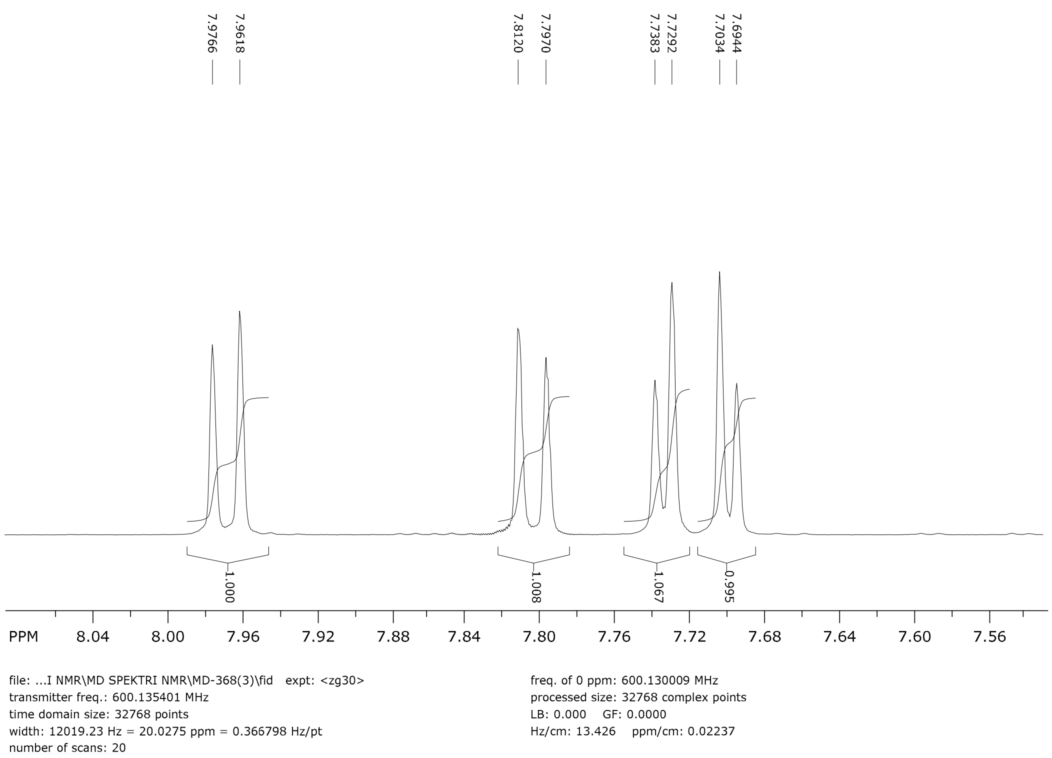

Figure S50. ^{1}H NMR ($CDCl_3$) spectrum of aromatic part of **13**.

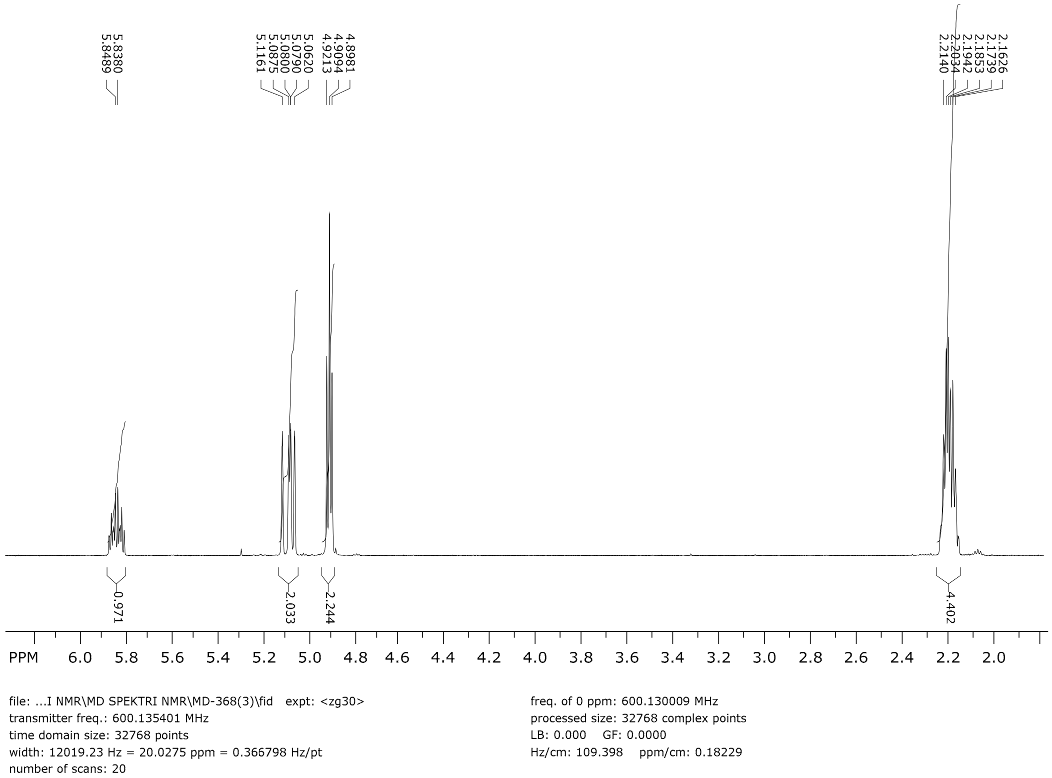

Figure S51. ^{1}H NMR ($CDCl_3$) spectrum of aliphatic part of **13**.

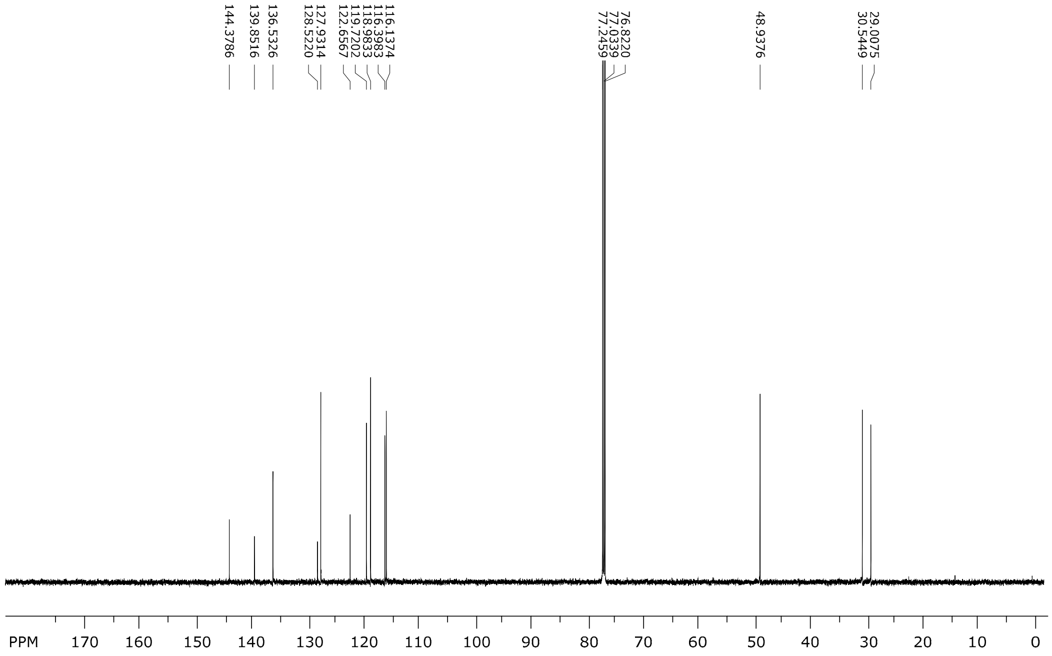

file: ...R\MD SPEKTRI NMR\MD-368(3) 13C\fid expt: <zgpg30>
transmitter freq.: 150.917899 MHz
time domain size: 65536 points
width: 35971.22 Hz = 238.3496 ppm = 0.548877 Hz/pt
number of scans: 573

freq. of 0 ppm: 150.902809 MHz
processed size: 32768 complex points
LB: 1.000 GF: 0.0000
Hz/cm: 1127.297 ppm/cm: 7.46960

Figure S52. ^{13}C NMR ($CDCl_3$) spectrum of **13**.

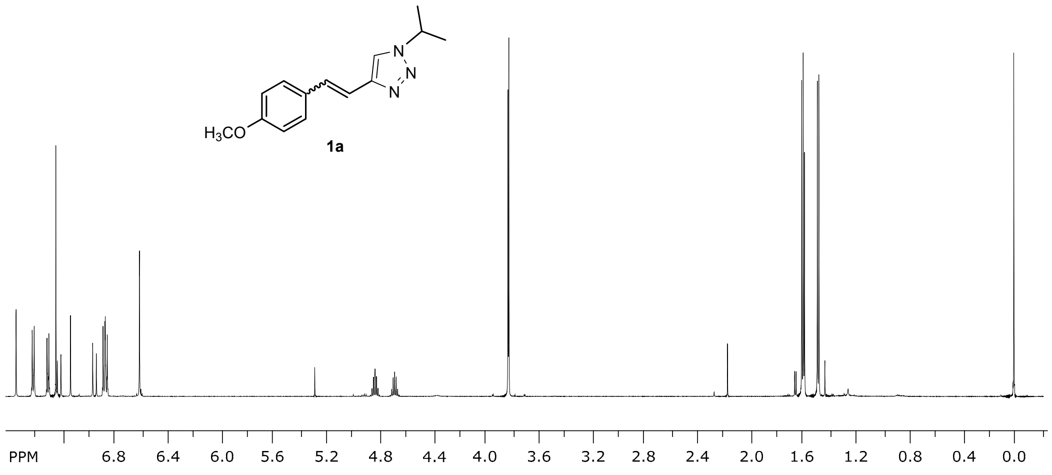

file: ...ža 2022-2023\NMR KK\KK-13(4-7)\fid expt: <zg30>
transmitter freq.: 600.135401 MHz
time domain size: 32768 points
width: 12019.23 Hz = 20.0275 ppm = 0.366798 Hz/pt
number of scans: 64

freq. of 0 ppm: 600.130010 MHz
processed size: 32768 complex points
LB: 0.000 GF: 0.0000
Hz/cm: 188.864 ppm/cm: 0.31470

Figure S53. ^{1}H NMR ($CDCl_3$) spectrum of **1a** (mixture of *cis*/*trans*-isomers).

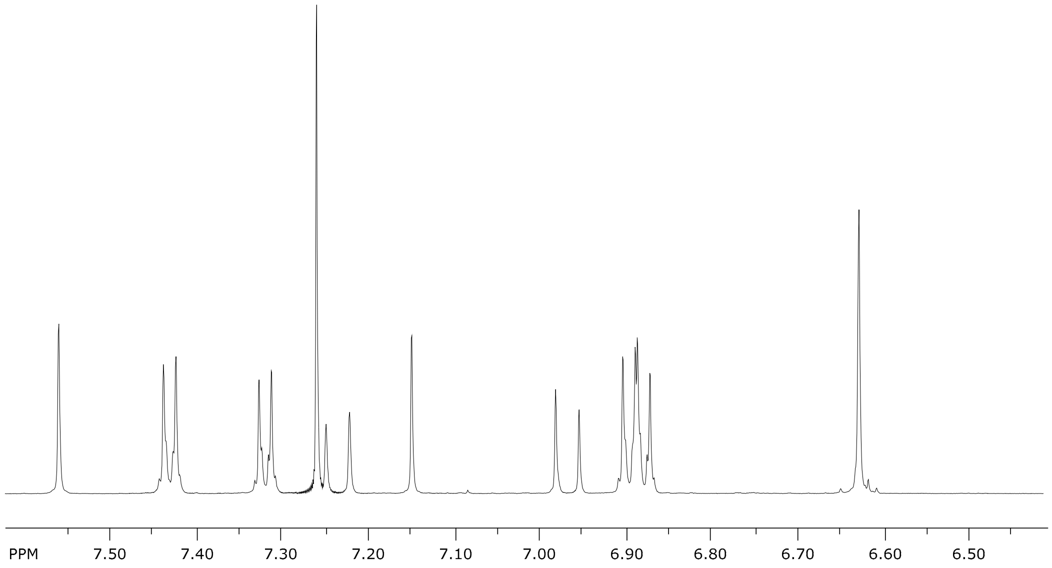

file: ...ža 2022-2023\NMR KK\KK-13(4-7)\fid expt: <zg30>
transmitter freq.: 600.135401 MHz
time domain size: 32768 points
width: 12019.23 Hz = 20.0275 ppm = 0.366798 Hz/pt
number of scans: 64

freq. of 0 ppm: 600.130010 MHz
processed size: 32768 complex points
LB: 0.000 GF: 0.0000
Hz/cm: 29.136 ppm/cm: 0.04855

Figure S54. ^{1}H NMR ($CDCl_3$) spectrum of aromatic part (mixture of *cis*/*trans*-isomers) of **1a**.

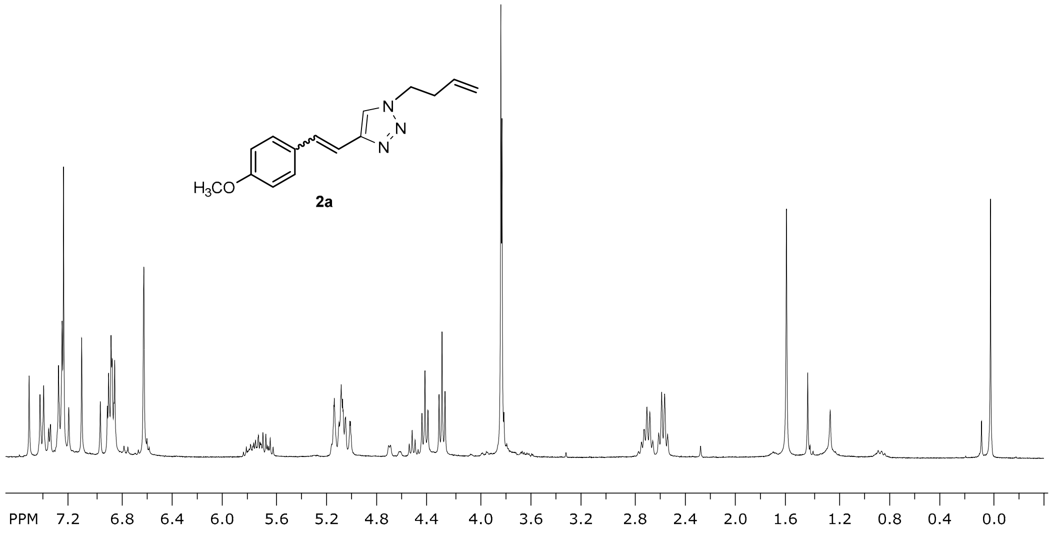

file: ...a 2022-2023\NMR KK\KK-4(10-12)\fid expt: <zg30>
transmitter freq.: 300.132701 MHz
time domain size: 32768 points
width: 6172.84 Hz = 20.5670 ppm = 0.188380 Hz/pt
number of scans: 64

freq. of 0 ppm: 300.130006 MHz
processed size: 32768 complex points
LB: 0.300 GF: 0.0000
Hz/cm: 97.677 ppm/cm: 0.32545

Figure S55. ^{1}H NMR ($CDCl_3$) spectrum of **2a** (mixture of *cis*/*trans*-isomers).

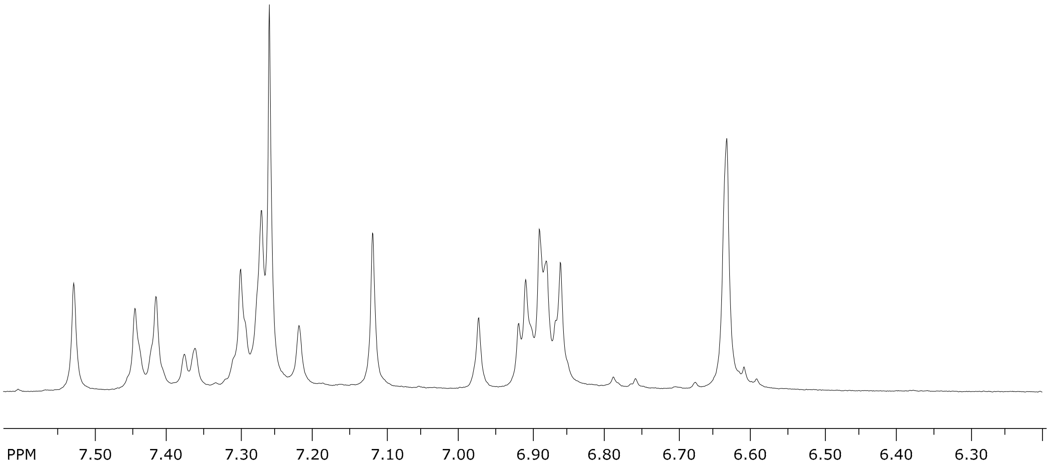

file: ...a 2022-2023\NMR KK\KK-4(10-12)\fid expt: <zg30>
transmitter freq.: 300.132701 MHz
time domain size: 32768 points
width: 6172.84 Hz = 20.5670 ppm = 0.188380 Hz/pt
number of scans: 64

freq. of 0 ppm: 300.130006 MHz
processed size: 32768 complex points
LB: 0.300 GF: 0.0000
Hz/cm: 17.167 ppm/cm: 0.05720

Figure S56. ^{1}H NMR ($CDCl_3$) spectrum of aromatic part (mixture of *cis*/*trans*-isomers) of **2a**.

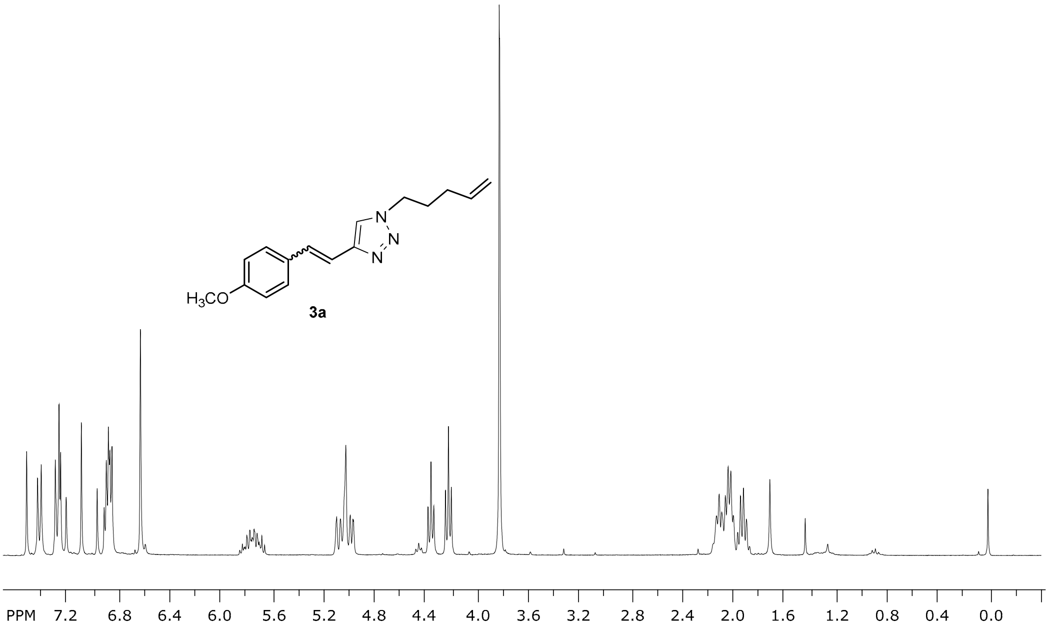

file: ...adža 2022-2023\NMR KK\KK-5(10)\fid expt: <zg30>
transmitter freq.: 300.132701 MHz
time domain size: 32768 points
width: 6172.84 Hz = 20.5670 ppm = 0.188380 Hz/pt
number of scans: 32

freq. of 0 ppm: 300.130005 MHz
processed size: 32768 complex points
LB: 0.300 GF: 0.0000
Hz/cm: 97.677 ppm/cm: 0.32545

Figure S57. ^{1}H NMR ($CDCl_3$) spectrum of **3a** (mixture of *cis*/*trans*-isomers).

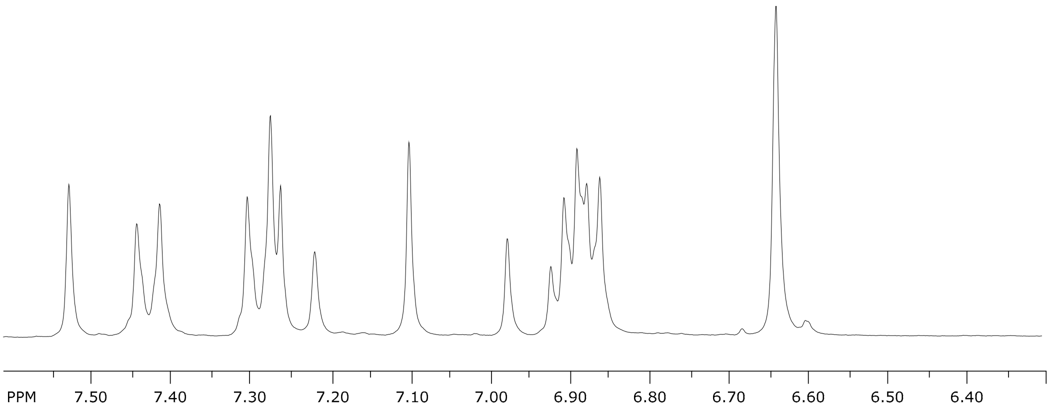

file: ...adža 2022-2023\NMR KK\KK-5(10)\fid expt: <zg30>
transmitter freq.: 300.132701 MHz
time domain size: 32768 points
width: 6172.84 Hz = 20.5670 ppm = 0.188380 Hz/pt
number of scans: 32

freq. of 0 ppm: 300.130005 MHz
processed size: 32768 complex points
LB: 0.300 GF: 0.0000
Hz/cm: 15.714 ppm/cm: 0.05236

Figure S58. ^{1}H NMR ($CDCl_3$) spectrum of aromatic part (mixture of *cis*/*trans*-isomers) of **3a**.

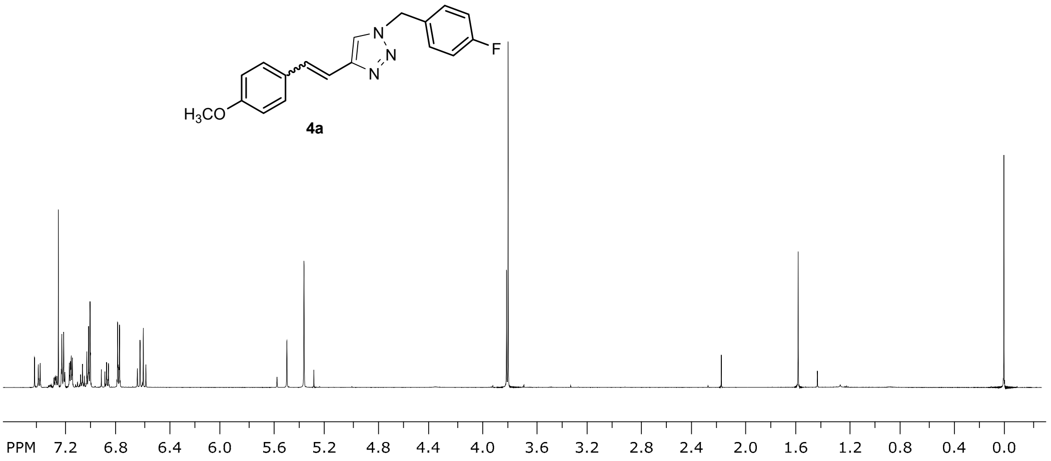

file: ...ža 2022-2023\NMR KK\KK-12(4-5)\fid expt: <zg30>
transmitter freq.: 600.135401 MHz
time domain size: 32768 points
width: 12019.23 Hz = 20.0275 ppm = 0.366798 Hz/pt
number of scans: 64

freq. of 0 ppm: 600.130011 MHz
processed size: 32768 complex points
LB: 0.000 GF: 0.0000
Hz/cm: 191.513 ppm/cm: 0.31912

Figure S59. ^{1}H NMR ($CDCl_3$) spectrum of **4a** (mixture of *cis*/*trans*-isomers).

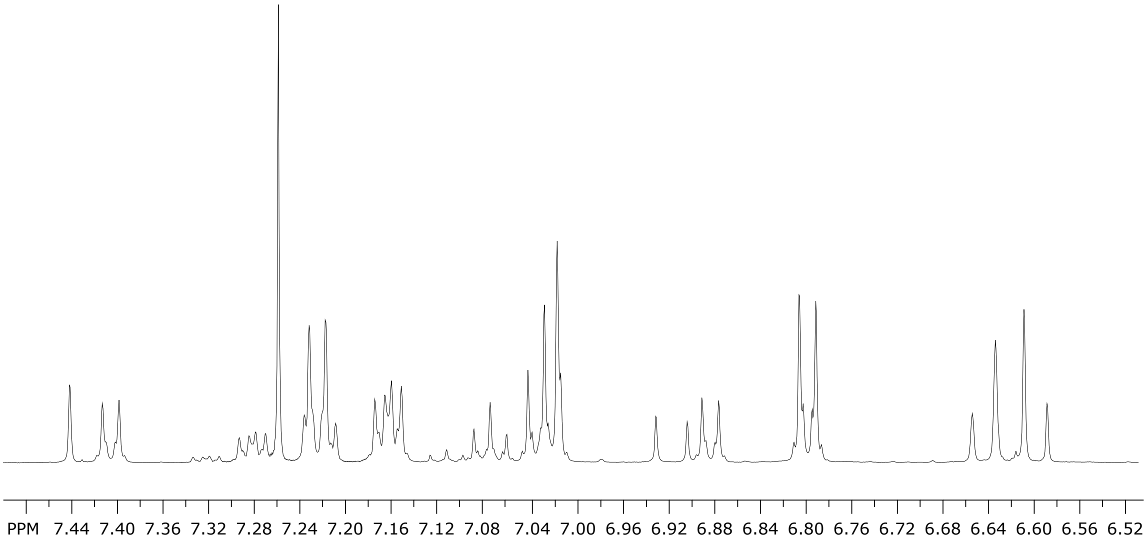

file: ...ža 2022-2023\NMR KK\KK-12(4-5)\fid expt: <zg30>
transmitter freq.: 600.135401 MHz
time domain size: 32768 points
width: 12019.23 Hz = 20.0275 ppm = 0.366798 Hz/pt
number of scans: 64

freq. of 0 ppm: 600.130011 MHz
processed size: 32768 complex points
LB: 0.000 GF: 0.0000
Hz/cm: 23.847 ppm/cm: 0.03974

Figure S60. ^{1}H NMR ($CDCl_3$) spectrum of aromatic part (mixture of *cis*/*trans*-isomers) of **4a**.

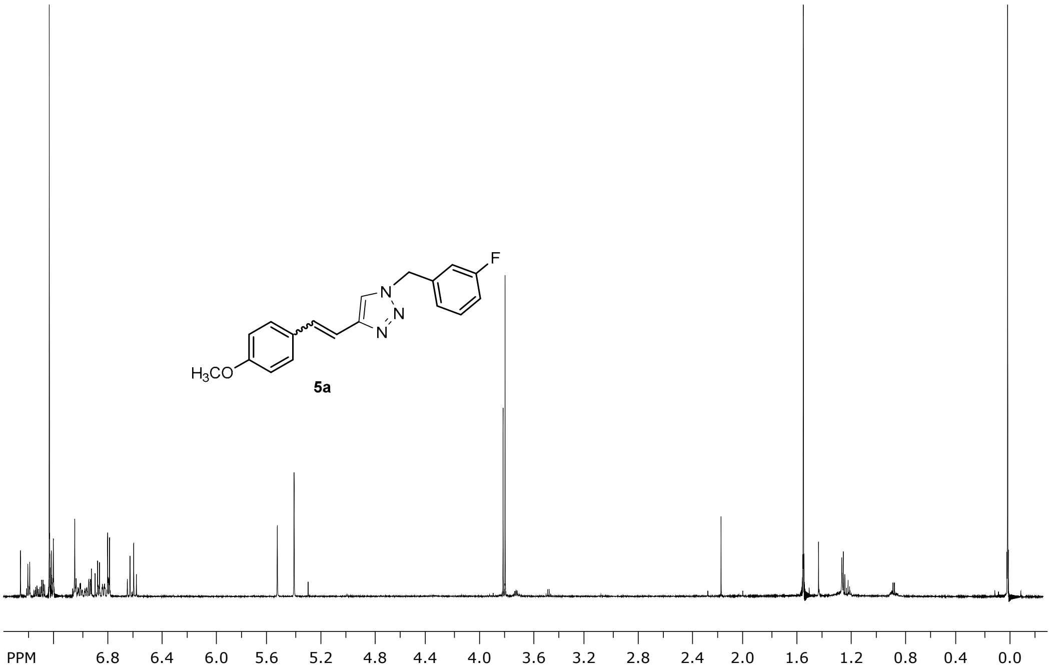

file: ...NMR\MD SPEKTRI NMR\MD-382(2-3)\fid expt: <zg30>
transmitter freq.: 600.135401 MHz
time domain size: 32768 points
width: 12019.23 Hz = 20.0275 ppm = 0.366798 Hz/pt
number of scans: 64

freq. of 0 ppm: 600.130011 MHz
processed size: 32768 complex points
LB: 0.000 GF: 0.0000
Hz/cm: 189.129 ppm/cm: 0.31514

Figure S61. ^{1}H NMR ($CDCl_3$) spectrum of **5a** (mixture of *cis*/*trans*-isomers).

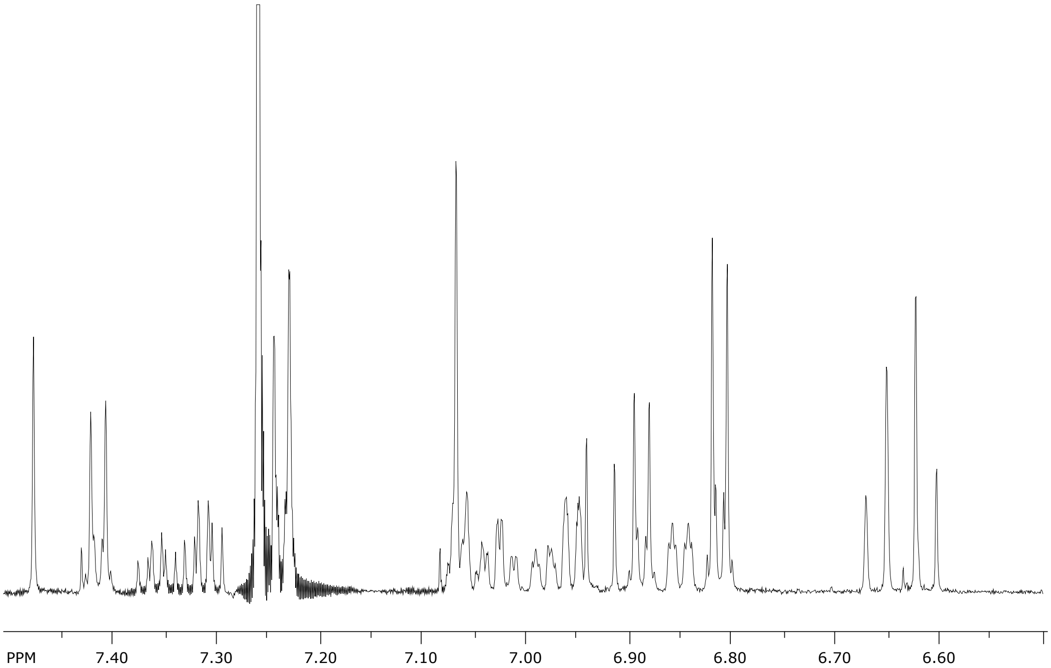

Figure S62. ^{1}H NMR ($CDCl_3$) spectrum of aromatic part (mixture of *cis*/*trans*-isomers) of **5a**.

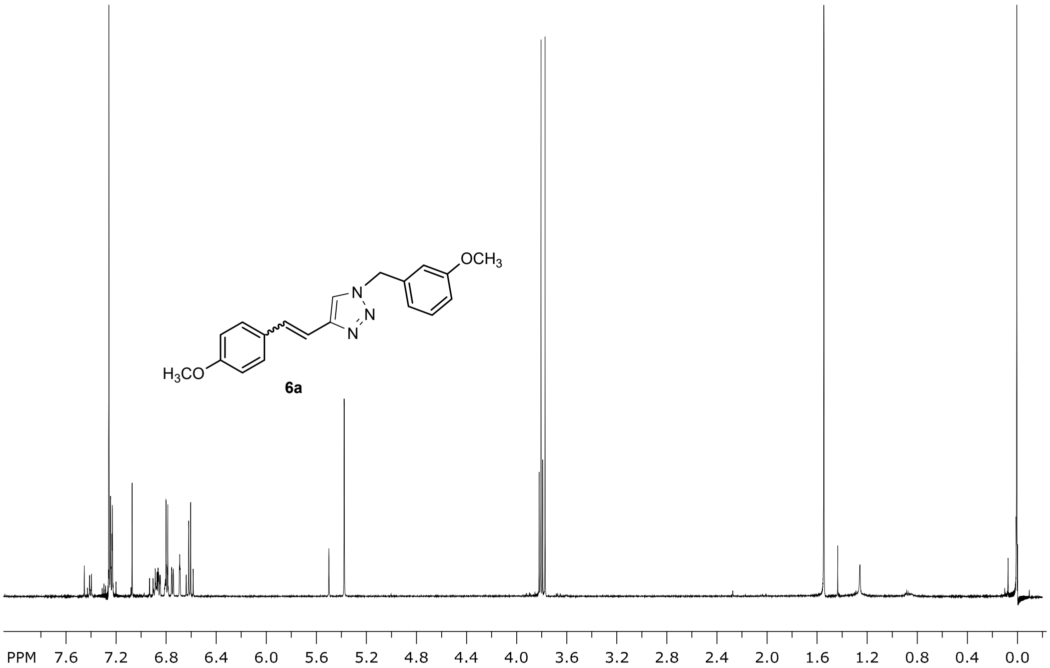

Figure S63. ^{1}H NMR ($CDCl_3$) spectrum of **6a** (mixture of *cis*/*trans*-isomers).

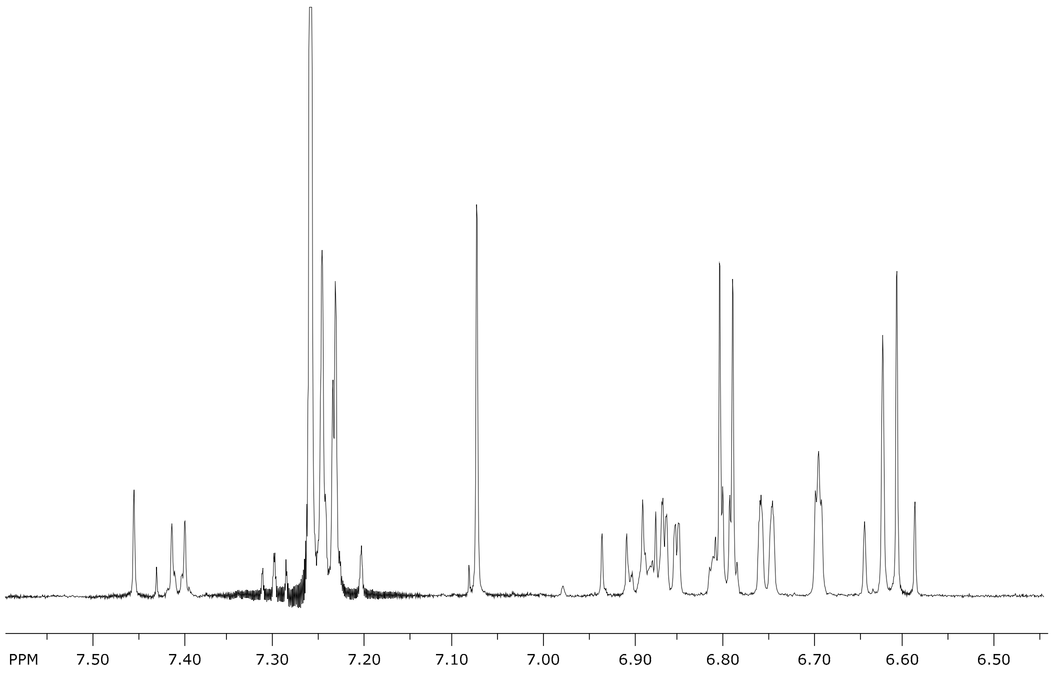

Figure S64. ^{1}H NMR ($CDCl_3$) spectrum of aromatic part (mixture of *cis*/*trans*-isomers) of **6a**.

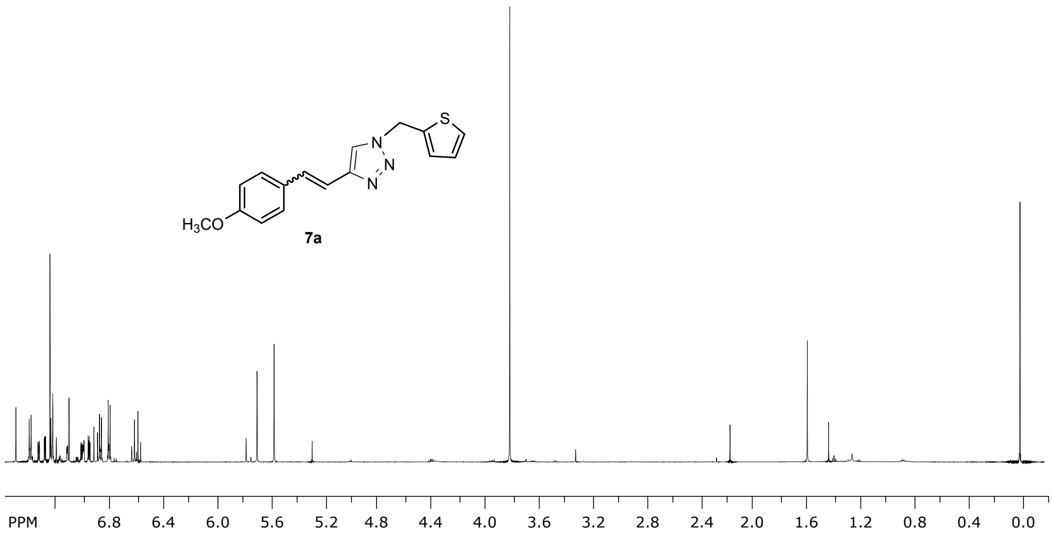

Figure S65. ^{1}H NMR ($CDCl_3$) spectrum of **7a** (mixture of *cis*/*trans*-isomers).

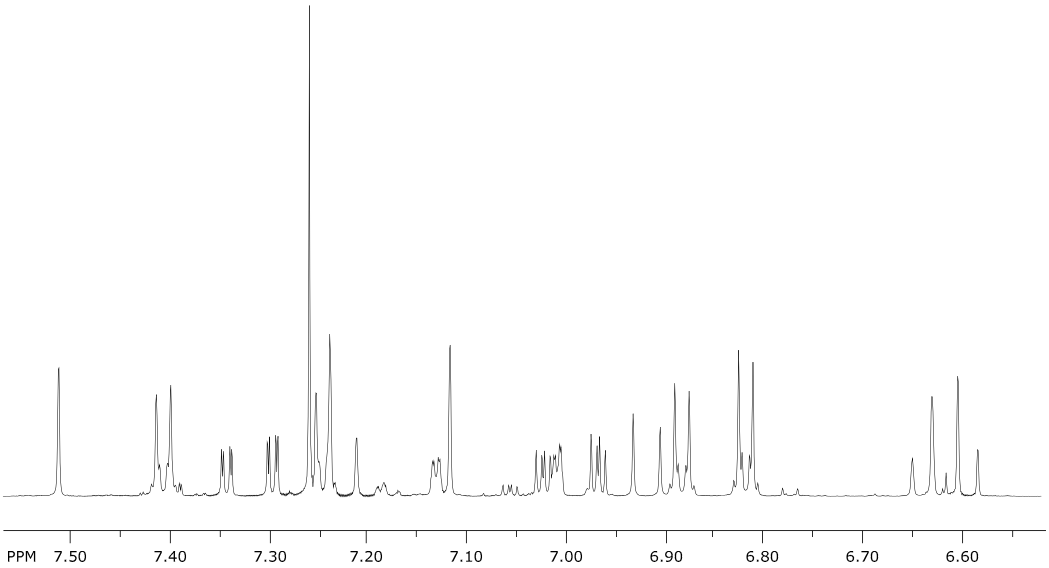

file: ...ža 2022-2023\NMR KK\KK-14(6-8)\fid expt: <zg30>
transmitter freq.: 600.135401 MHz
time domain size: 32768 points
width: 12019.23 Hz = 20.0275 ppm = 0.366798 Hz/pt
number of scans: 64

freq. of 0 ppm: 600.130011 MHz
processed size: 32768 complex points
LB: 0.000 GF: 0.0000
Hz/cm: 25.311 ppm/cm: 0.04218

Figure S66. ^{1}H NMR ($CDCl_3$) spectrum of aromatic part (mixture of *cis*/*trans*-isomers) of **7a**.

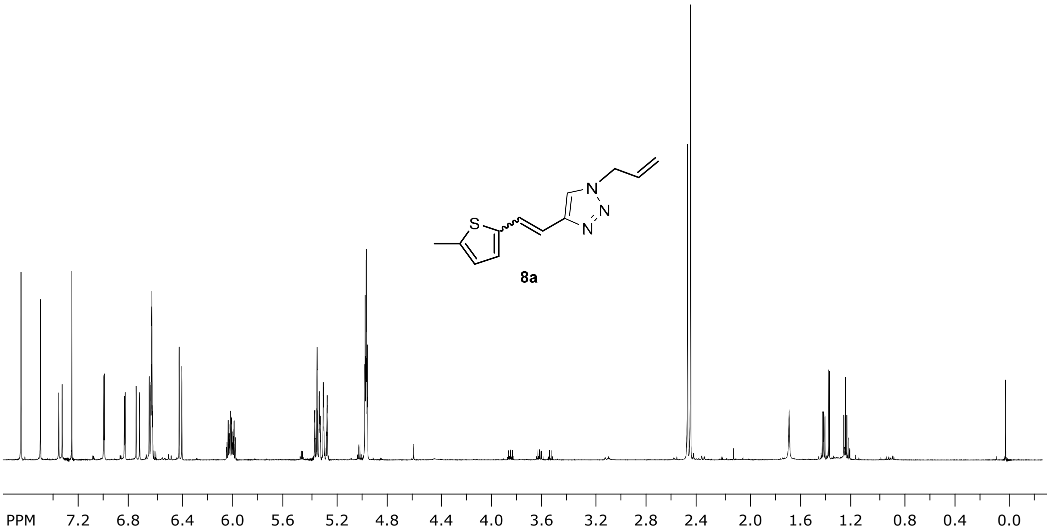

file: ...I NMR\MD SPEKTRI NMR\MD-396(3)\fid expt: <zg30>
transmitter freq.: 600.135401 MHz
time domain size: 32768 points
width: 12019.23 Hz = 20.0275 ppm = 0.366798 Hz/pt
number of scans: 32

freq. of 0 ppm: 600.130009 MHz
processed size: 32768 complex points
LB: 0.000 GF: 0.0000
Hz/cm: 194.162 ppm/cm: 0.32353

Figure S67. ^{1}H NMR ($CDCl_3$) spectrum of **8a** (mixture of *cis*/*trans*-isomers).

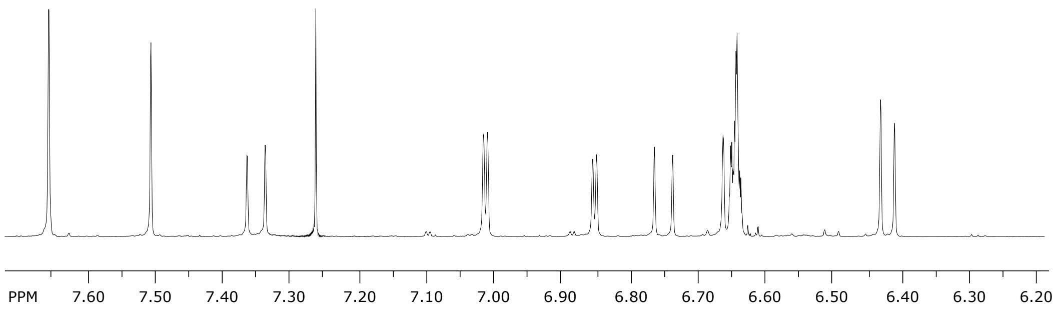

file: ...I NMR\MD SPEKTRI NMR\MD-396(3)\fid expt: <zg30>
transmitter freq.: 600.135401 MHz
time domain size: 32768 points
width: 12019.23 Hz = 20.0275 ppm = 0.366798 Hz/pt
number of scans: 32

freq. of 0 ppm: 600.130009 MHz
processed size: 32768 complex points
LB: 0.000 GF: 0.0000
Hz/cm: 36.907 ppm/cm: 0.06150

Figure S68. ^{1}H NMR (CDCl$_3$) spectrum of aromatic part (mixture of *cis*/*trans*-isomers) of **8a**.

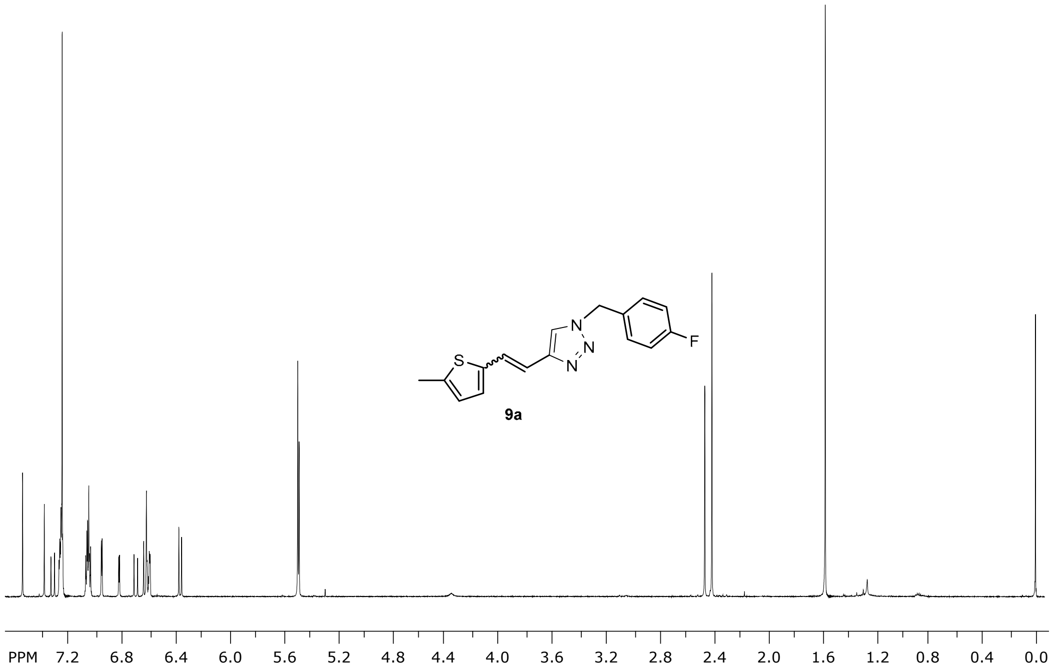

file: ...ić 2022-2023\NMR DM\DM-6(9-13)\fid expt: <zg30>
transmitter freq.: 600.135401 MHz
time domain size: 32768 points
width: 12019.23 Hz = 20.0275 ppm = 0.366798 Hz/pt
number of scans: 64

freq. of 0 ppm: 600.130010 MHz
processed size: 32768 complex points
LB: 0.000 GF: 0.0000
Hz/cm: 186.215 ppm/cm: 0.31029

Figure S69. ^{1}H NMR (CDCl$_3$) spectrum of **9a** (mixture of *cis*/*trans*-isomers).

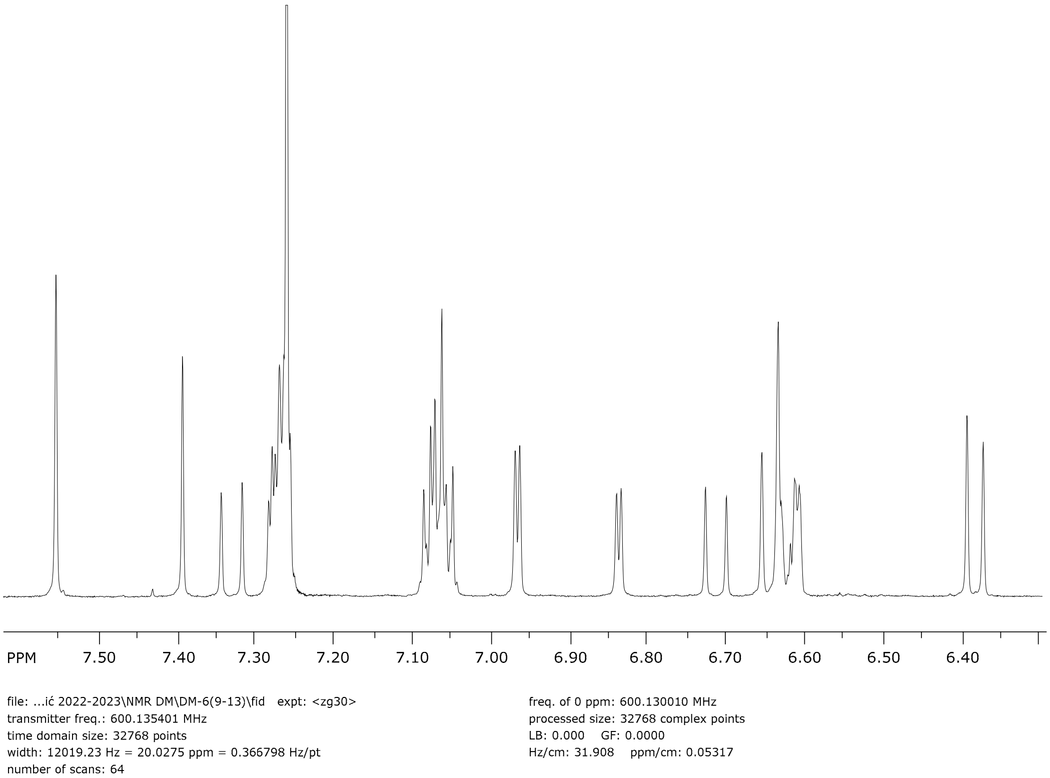

Figure S70. ^{1}H NMR (CDCl$_3$) spectrum of aromatic part (mixture of *cis*/*trans*-isomers) of **9a**.

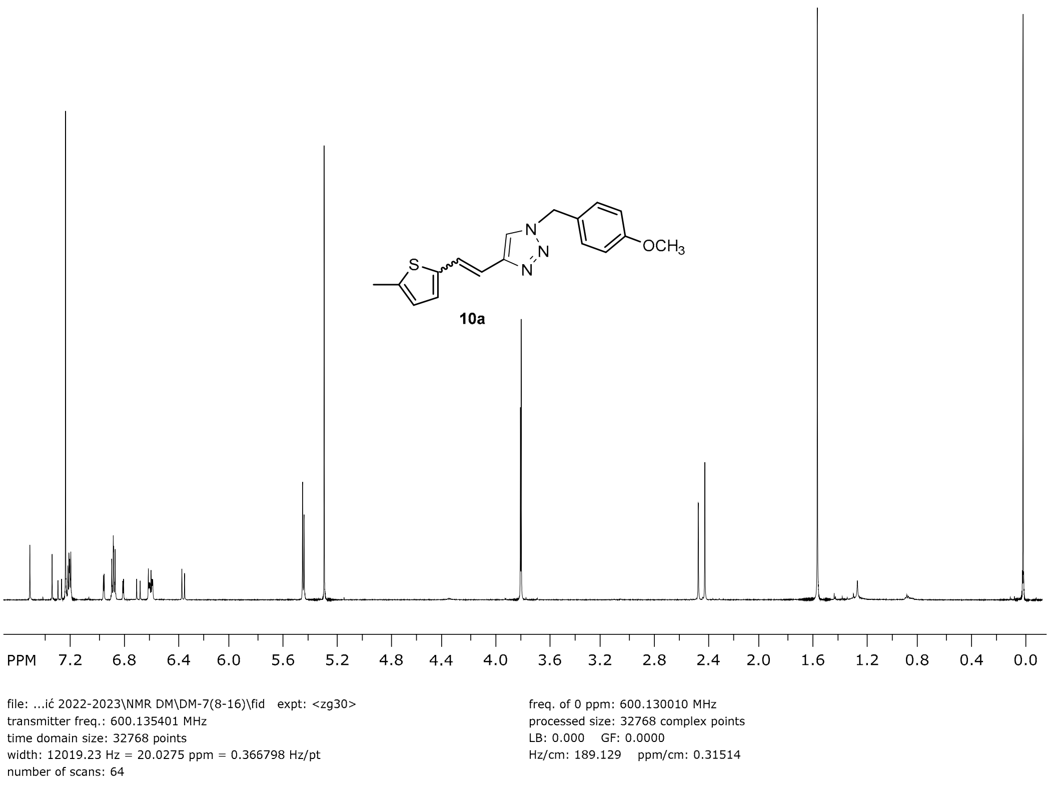

Figure S71. ^{1}H NMR (CDCl$_3$) spectrum of **10a** (mixture of *cis*/*trans*-isomers).

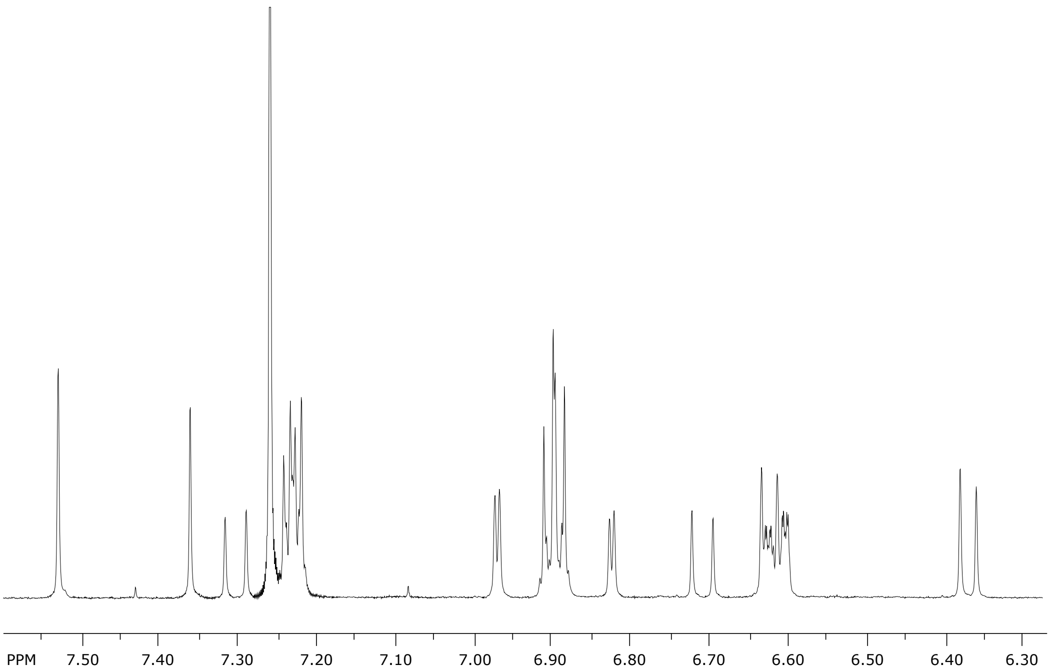

file: ...ić 2022-2023\NMR DM\DM-7(8-16)\fid expt: <zg30>
transmitter freq.: 600.135401 MHz
time domain size: 32768 points
width: 12019.23 Hz = 20.0275 ppm = 0.366798 Hz/pt
number of scans: 64

freq. of 0 ppm: 600.130010 MHz
processed size: 32768 complex points
LB: 0.000 GF: 0.0000
Hz/cm: 31.886 ppm/cm: 0.05313

Figure S72. ^{1}H NMR ($CDCl_3$) spectrum of aromatic part (mixture of *cis*/*trans*-isomers) of **10a**.

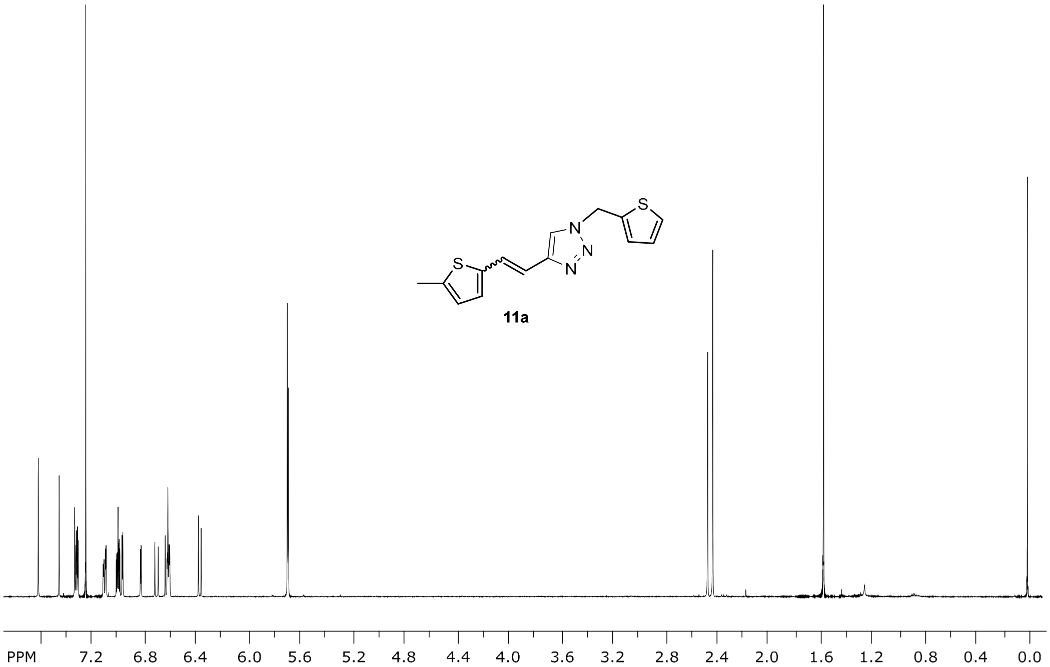

file: ...vić 2022-2023\NMR DM\DM-5(7-9)\fid expt: <zg30>
transmitter freq.: 600.135401 MHz
time domain size: 32768 points
width: 12019.23 Hz = 20.0275 ppm = 0.366798 Hz/pt
number of scans: 64

freq. of 0 ppm: 600.130011 MHz
processed size: 32768 complex points
LB: 0.000 GF: 0.0000
Hz/cm: 192.308 ppm/cm: 0.32044

Figure S73. ^{1}H NMR ($CDCl_3$) spectrum of **11a** (mixture of *cis*/*trans*-isomers).

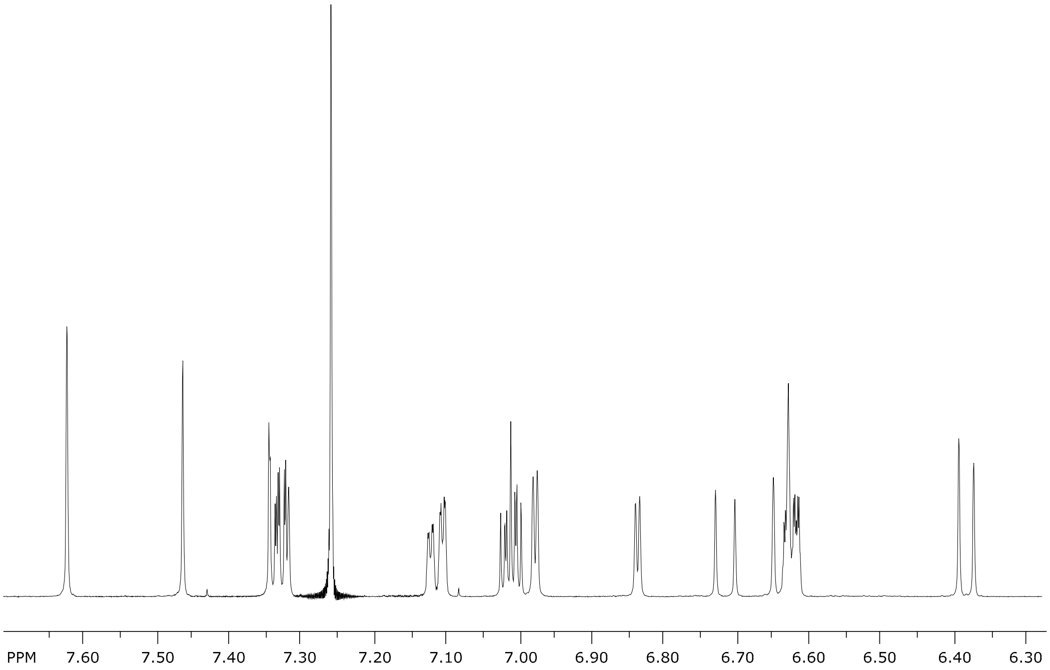

file: ...vić 2022-2023\NMR DM\DM-5(7-9)\fid expt: <zg30>
transmitter freq.: 600.135401 MHz
time domain size: 32768 points
width: 12019.23 Hz = 20.0275 ppm = 0.366798 Hz/pt
number of scans: 64

freq. of 0 ppm: 600.130011 MHz
processed size: 32768 complex points
LB: 0.000 GF: 0.0000
Hz/cm: 34.541 ppm/cm: 0.05756

Figure S74. ^{1}H NMR ($CDCl_3$) spectrum of aromatic part (mixture of *cis*/*trans*-isomers) of **11a**.

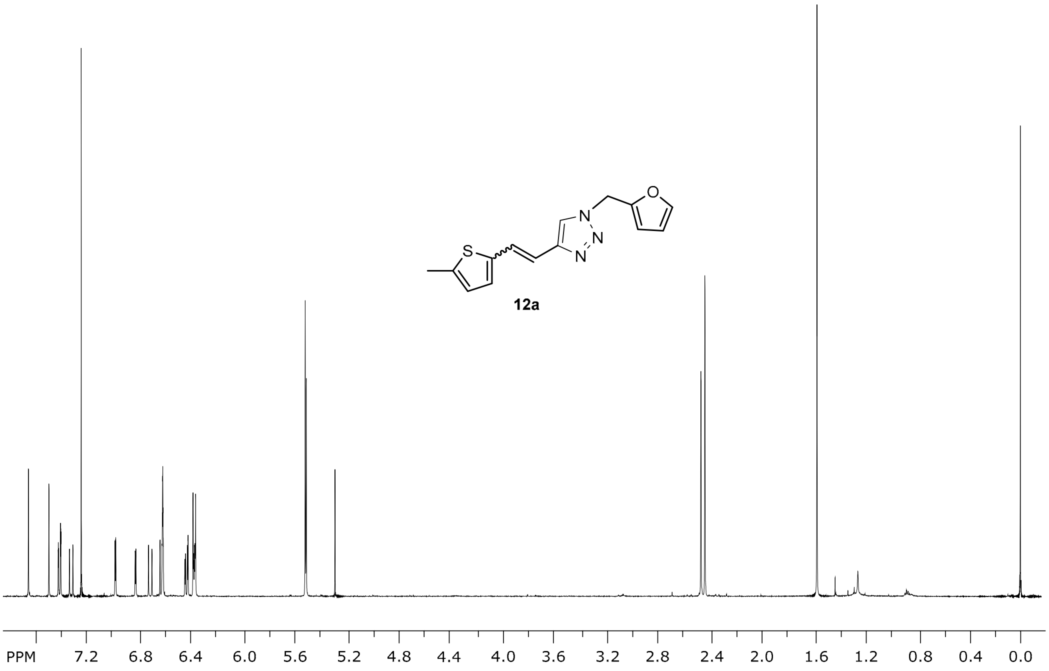

file: ...vić 2022-2023\NMR DM\DM-8(6-8)\fid expt: <zg30>
transmitter freq.: 600.135401 MHz
time domain size: 32768 points
width: 12019.23 Hz = 20.0275 ppm = 0.366798 Hz/pt
number of scans: 64

freq. of 0 ppm: 600.130009 MHz
processed size: 32768 complex points
LB: 0.000 GF: 0.0000
Hz/cm: 192.755 ppm/cm: 0.32119

Figure S75. ^{1}H NMR ($CDCl_3$) spectrum of **12a** (mixture of *cis*/*trans*-isomers).

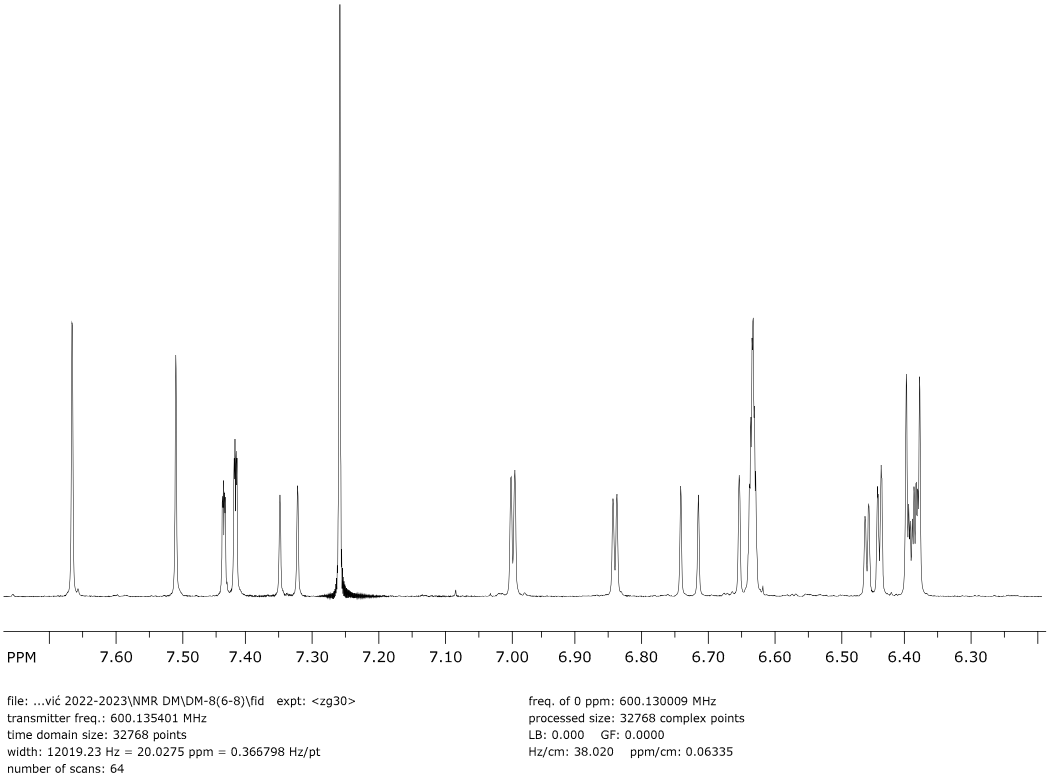

Figure S76. ^{1}H NMR ($CDCl_3$) spectrum of aromatic part (mixture of *cis*/*trans*-isomers) of **12a**.

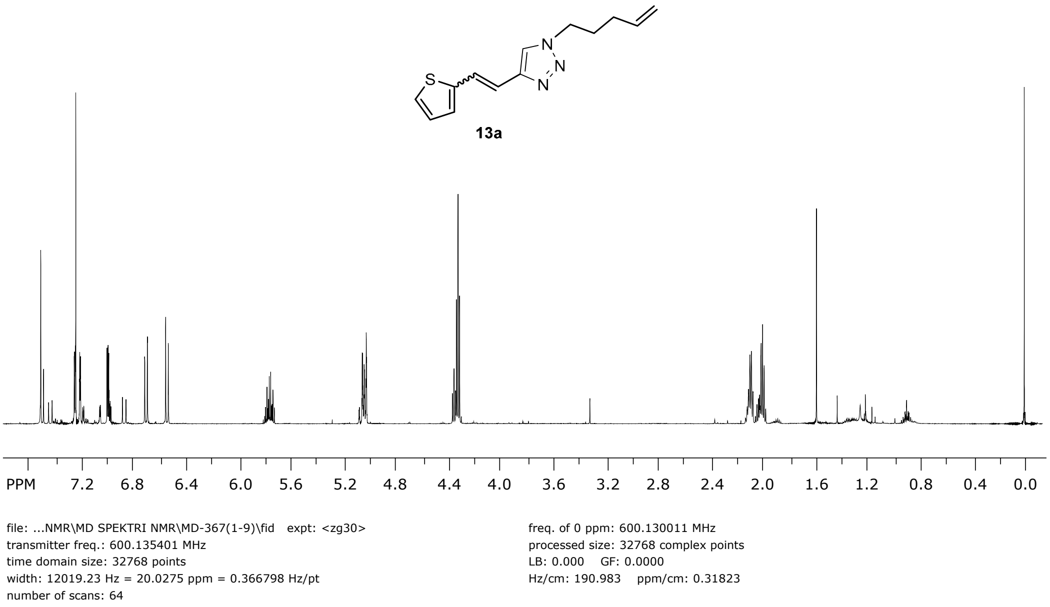

Figure S77. ^{1}H NMR ($CDCl_3$) spectrum of **13a** (mixture of *cis*/*trans*-isomers).

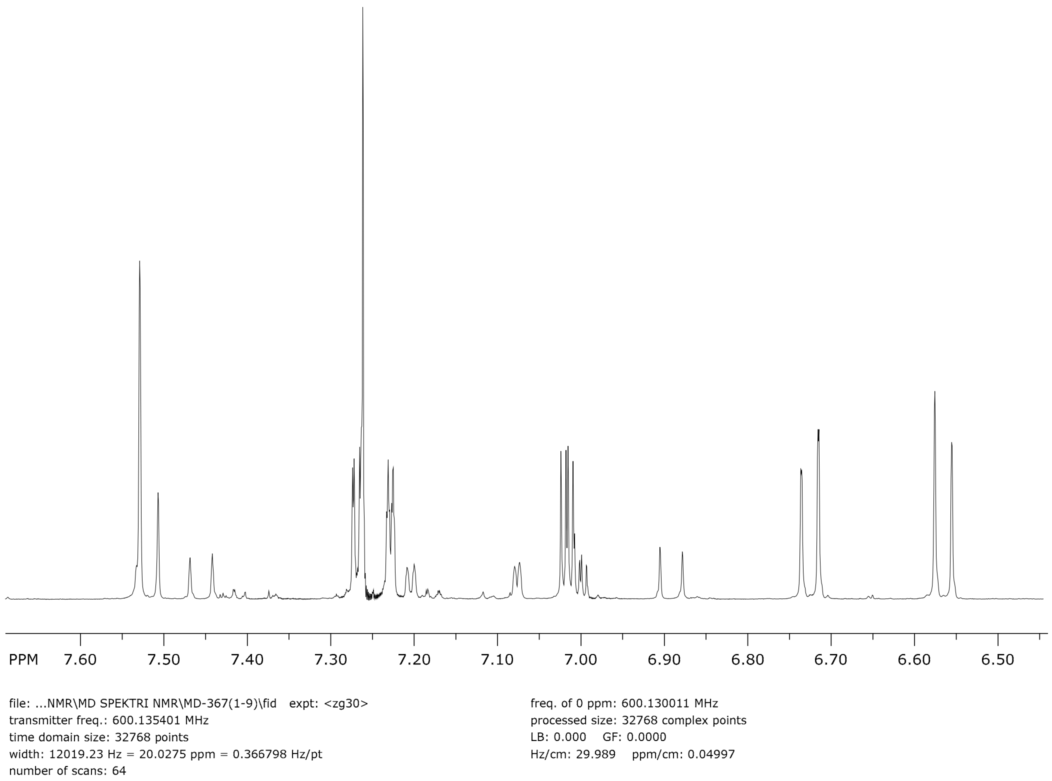

Figure S78. ^{1}H NMR ($CDCl_3$) spectrum of aromatic part (mixture of *cis*/*trans*-isomers) of **13a**.

2. MS spectra and HRMS analyses of naphtho- and thienobenzo-triazoles

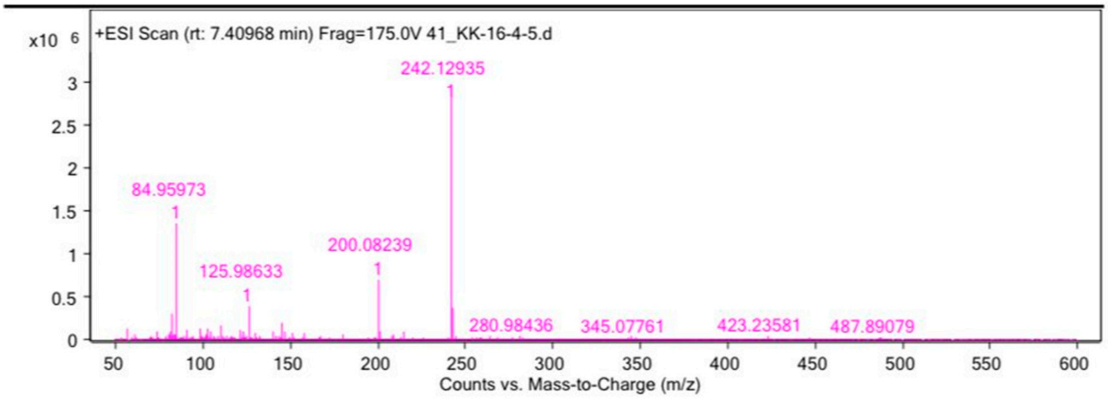

Formula Calculator Results

Formula	Best	Mass	Tgt Mass	Diff (ppm)	Ion Species	Score
C14 H15 N3 O	True	241.12213	241.12151	-2.54	C14 H16 N3 O	93.87

Figure S79. MS spectrum and HRMS analysis of **1**.

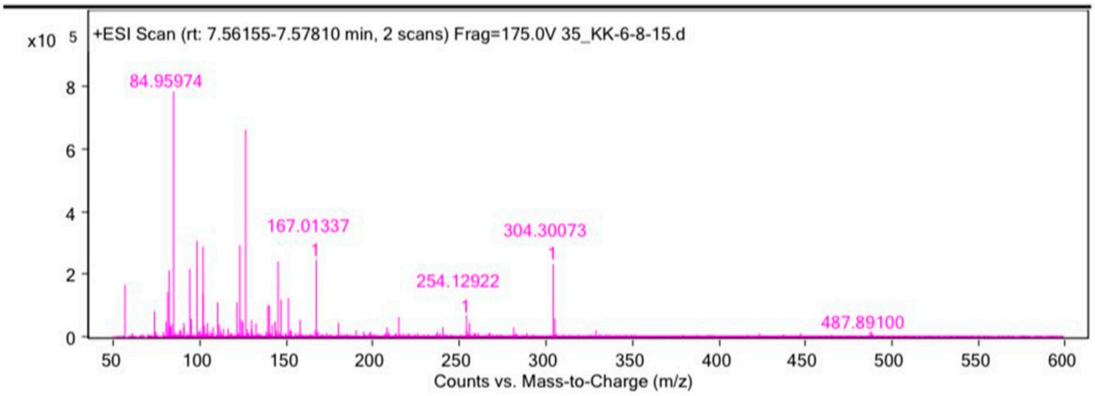

Formula Calculator Results

Formula	Best	Mass	Tgt Mass	Diff (ppm)	Ion Species	Score
C15 H15 N3 O	True	253.12216	253.12151	-2.58	C15 H16 N3 O	94.3

Figure S80. MS spectrum and HRMS analysis of **2**.

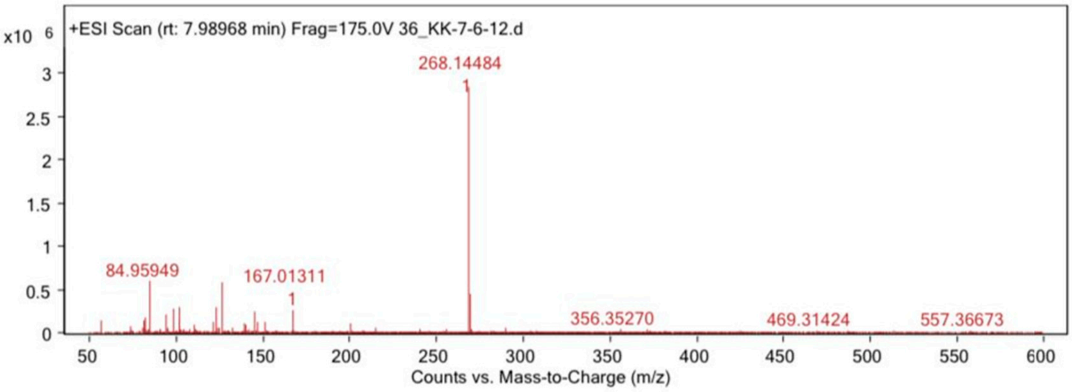

Formula Calculator Results

Formula	Best	Mass	Tgt Mass	Diff (ppm)	Ion Species	Score
C16 H17 N3 O	True	267.13763	267.13716	-1.74	C16 H18 N3 O	96.33

Figure S81. MS spectrum and HRMS analysis of **3**.

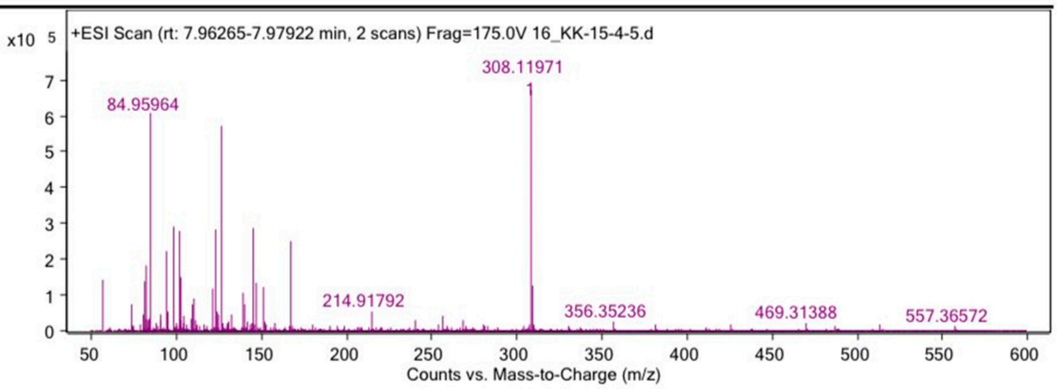

Formula Calculator Results

Formula	Best	Mass	Tgt Mass	Diff (ppm)	Ion Species	Score
C18 H14 F N3 O	True	307.11245	307.11209	-1.17	C18 H15 F N3 O	98.6

Figure S82. MS spectrum and HRMS analysis of **4**.

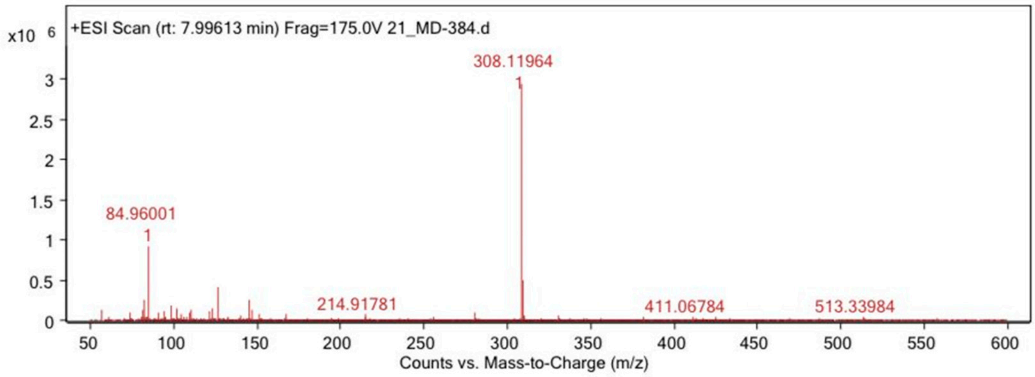

Formula Calculator Results

Formula	Best	Mass	Tgt Mass	Diff (ppm)	Ion Species	Score
C18 H14 F N3 O	True	307.11249	307.11209	-1.3	C18 H15 F N3 O	94.97

Figure S83. MS spectrum and HRMS analysis of **5**.

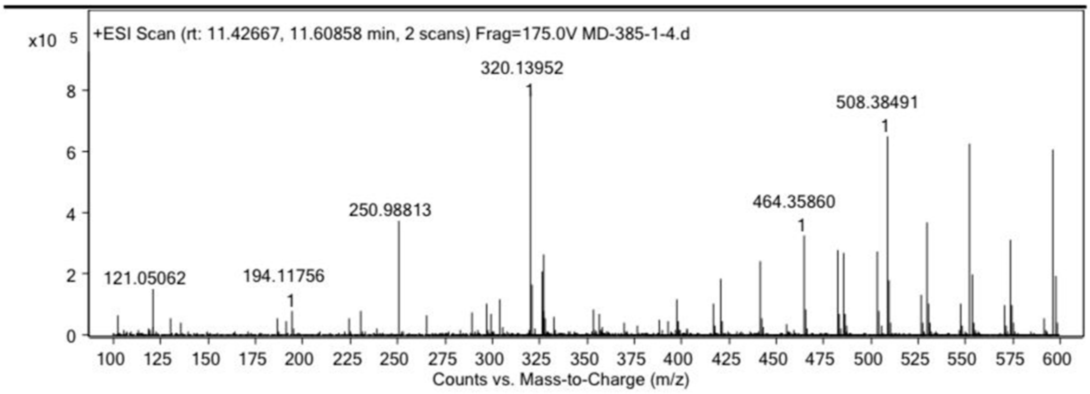

Formula Calculator Results

Formula	Best	Mass	Tgt Mass	Diff (ppm)	Ion Species	Score
C19 H17 N3 O2	True	319.13227	319.13208	-0.59	C19 H18 N3 O2	99.38

Figure S84. MS spectrum and HRMS analysis of **6**.

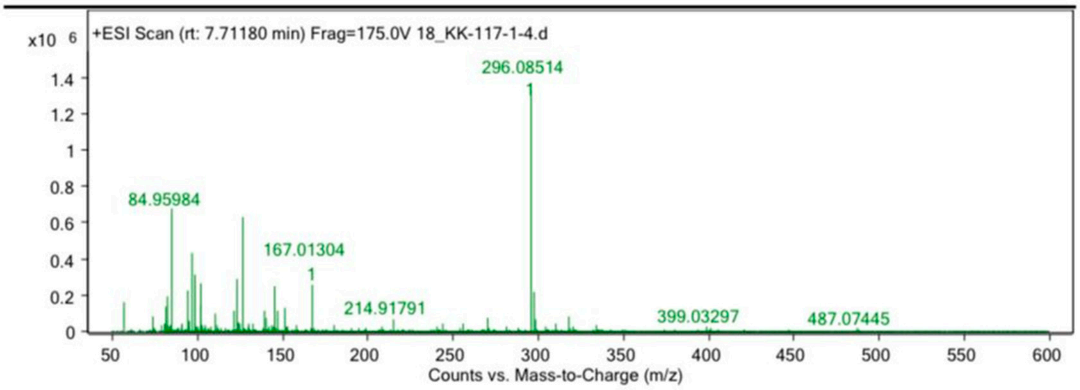

Formula Calculator Results

Formula	Best	Mass	Tgt Mass	Diff (ppm)	Ion Species	Score
C16 H13 N3 O S	True	295.07812	295.07793	-0.64	C16 H14 N3 O S	93.67

Figure S85. MS spectrum and HRMS analysis of **7**.

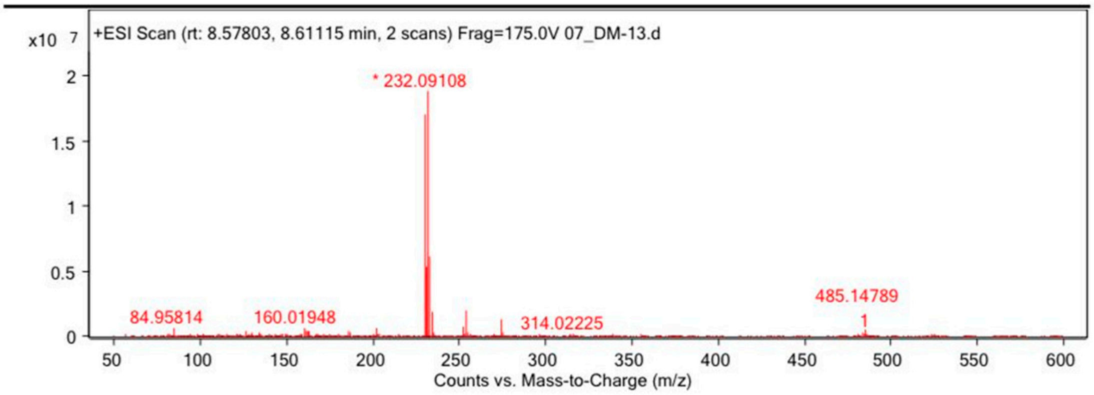

Formula Calculator Results

Formula	Best	Mass	Tgt Mass	Diff (ppm)	Ion Species	Score
C12 H11 N3 S	True	229.0679	229.06737	-2.33	C12 H12 N3 S	46.38

Figure S86. MS spectrum and HRMS analysis of **8**.

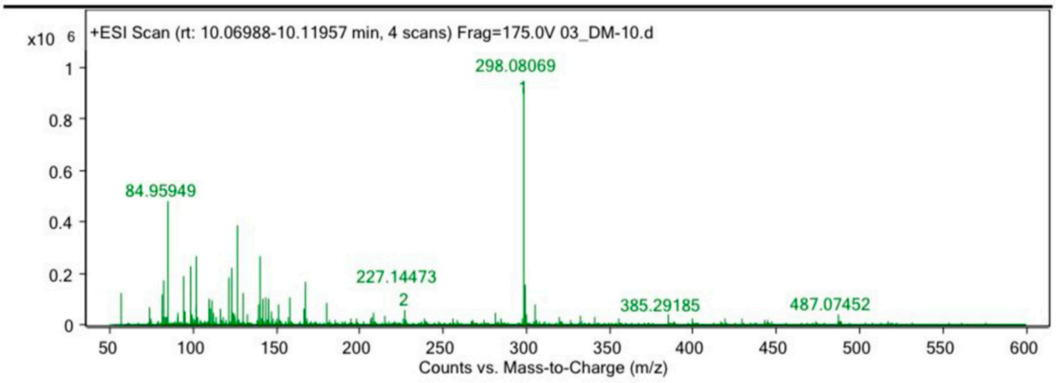

Formula Calculator Results

Formula	Best	Mass	Tgt Mass	Diff (ppm)	Ion Species	Score
C16 H12 F N3 S	True	297.07351	297.0736	0.29	C16 H13 F N3 S	97.42

Figure S87. MS spectrum and HRMS analysis of **9**.

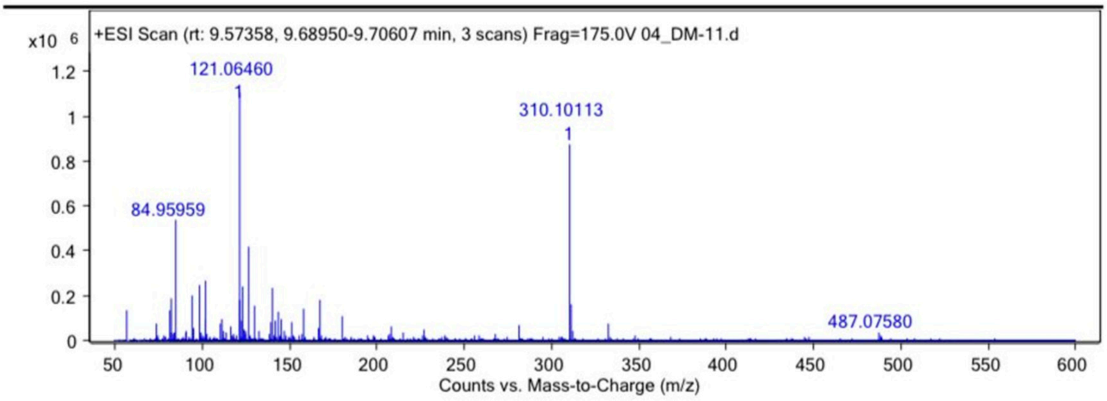

Formula Calculator Results

Formula	Best	Mass	Tgt Mass	Diff (ppm)	Ion Species	Score
C17 H15 N3 O S	True	309.09399	309.09358	-1.33	C17 H16 N3 O S	96.47

Figure S88. MS spectrum and HRMS analysis of **10**.

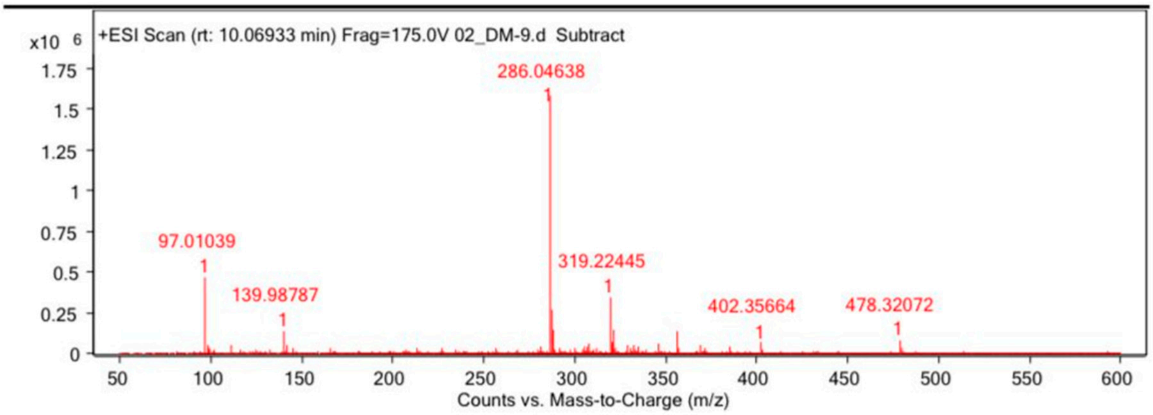

Formula Calculator Results

Formula	Best	Mass	Tgt Mass	Diff (ppm)	Ion Species	Score
C14 H11 N3 S2	True	285.0392	285.03944	0.84	C14 H12 N3 S2	97.91

Figure S89. MS spectrum and HRMS analysis of **11**.

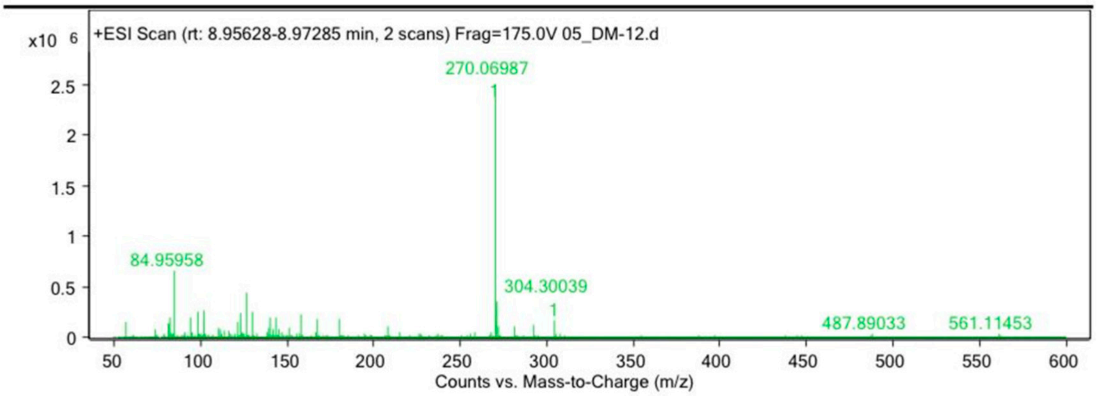

Formula Calculator Results

Formula	Best	Mass	Tgt Mass	Diff (ppm)	Ion Species	Score
C14 H11 N3 O S	True	269.06269	269.06228	-1.5	C14 H12 N3 O S	96.45

Figure S90. MS spectrum and HRMS analysis of **12**.

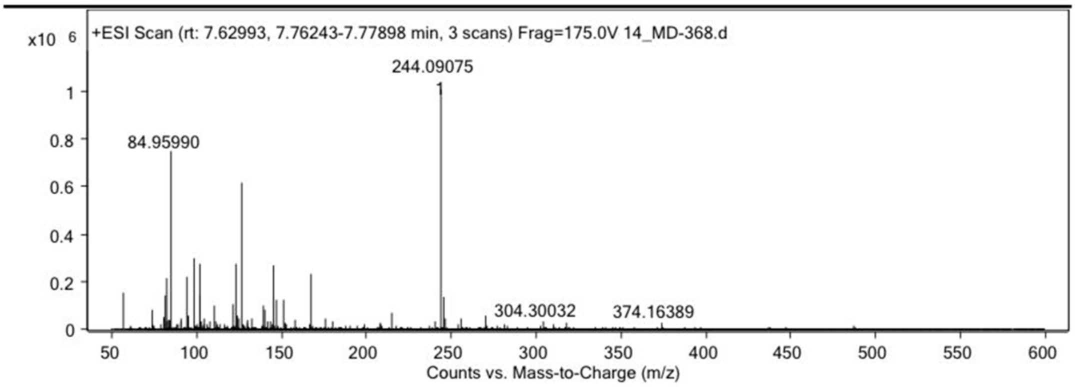

Formula Calculator Results

Formula	Best	Mass	Tgt Mass	Diff (ppm)	Ion Species	Score
C13 H13 N3 S	True	243.0835	243.08302	-1.97	C13 H14 N3 S	96.6

Figure S91. MS spectrum and HRMS analysis of **13**.

Table S1. Free energies of binding, ΔG_{bind} obtained by molecular docking of listed molecules into the active site of AchE (4EY7.pdb), along with the number of conformational clusters and distribution of conformations.

Ligand	ΔG_{bind}/kcal mol^{-1}		Number of distinctive conformational clusters	Distribution of conformations within clusters with $n > 1$ (n = cluster population)	Experiment IC$_{50}$/μM
	lowest	highest			
**3**	−8.91	−8.14	4	4, 3, 17	51.3
**5**	−9.53	−9.05	4	17, 3, 4	55.5
Donepezil	−11.37	−10.75	4	22	-
Galantamine	−10.11	−10.10	1	25	0.15

Table S2. Free energies of binding, ΔG_{bind} obtained by molecular docking of listed molecules into the active site of BchE (1P0I.pdb), along with the number of conformational clusters and distribution of conformations.

Ligand	ΔG_{bind}/kcal mol^{-1}		Number of distinctive conformational clusters	Distribution of conformations within clusters with $n > 1$ (n = cluster population)	Experiment IC$_{50}$/μM
	lowest	highest			
**3**	−7.56	−6.96	6	10, 3, 9	53.5
**13**	−6.81	−6.02	6	16, 2, 4	40.7
Donepezil	−9.58	−8.29	3	9, 8, 4	-
Galantamine	−7.49	−7.44	1	25	7.9

Table S3. Root-mean-square displacement (RMSD), RMS fluctuations of alpha carbons of the protein backbone, and radius of gyration for complexes of AChE and ligands listed in the table, derived by molecular dynamics simulation of 30 ns.

Ligand	RMSD/Å		RMSF/Å		Rg/Å	
	average	min, max	average	min, max	average	min, max
**3**	1.69	0.78, 2.01	0.66	0.34, 1.75	22.77	22.57, 22.93
**5**	1.74	0.77, 2.19	0.70	0.32, 2.13	22.84	22.60, 23.08

Table S4. Root-mean-square displacement (RMSD), RMS fluctuations of alpha carbons of the protein backbone, and radius of gyration for complexes of BChE and ligands listed in the table, derived by molecular dynamics simulation of 30 ns.

Ligand	RMSD/Å		RMSF/Å		Rg/Å	
	average	min, max	average	min, max	average	min, max
**3**	1.97	0.85, 2.43	0.73	0.33, 2.82	22.86	22.66, 22.08
**13**	1.74	0.75, 2.17	0.77	0.34, 2.47	22.99	22.74, 23.22
